# Sequence tolerance of immunoglobulin variable domain framework regions to noncanonical intradomain disulfide linkages

**DOI:** 10.1016/j.jbc.2023.105278

**Published:** 2023-09-22

**Authors:** Dae Young Kim, Hiba Kandalaft, Michael J. Lowden, Qingling Yang, Martin A. Rossotti, Anna Robotham, John F. Kelly, Greg Hussack, Joseph D. Schrag, Kevin A. Henry, Jamshid Tanha

**Affiliations:** 1 Life Sciences Division, Human Health Therapeutics Research Centre, National Research Council Canada, Ottawa, Ontario, Canada; 2 Life Sciences Division, Human Health Therapeutics Research Centre, National Research Council Canada, Montréal, Quebec, Canada; 3 Department of Biochemistry, Microbiology and Immunology, Faculty of Medicine, University of Ottawa, Ottawa, Ontario, Canada

**Keywords:** disulfide, protein engineering, protein stability, immunoglobulin variable domain, single-domain antibody (sdAb, nanobody), phage display

## Abstract

Most immunoglobulin (Ig) domains bear only a single highly conserved canonical intradomain, inter-β-sheet disulfide linkage formed between Cys23-Cys104, and incorporation of rare noncanonical disulfide linkages at other locations can enhance Ig domain stability. Here, we exhaustively surveyed the sequence tolerance of Ig variable (V) domain framework regions (FRs) to noncanonical disulfide linkages. Starting from a destabilized V_H_ domain lacking a Cys23-Cys104 disulfide linkage, we generated and screened phage-displayed libraries of engineered V_H_s, bearing all possible pairwise combinations of Cys residues in neighboring β-strands of the Ig fold FRs. This approach identified seven novel Cys pairs in V_H_ FRs (Cys4-Cys25, Cys4-Cys118, Cys5-Cys120, Cys6-Cys119, Cys22-Cys88, Cys24-Cys86, and Cys45-Cys100; the international ImMunoGeneTics information system numbering), whose presence rescued domain folding and stability. Introduction of a subset of these noncanonical disulfide linkages (three intra-β-sheet: Cys4-Cys25, Cys22-Cys88, and Cys24-Cys86, and one inter-β-sheet: Cys6-Cys119) into a diverse panel of V_H_, V_L_, and V_H_H domains enhanced their thermostability and protease resistance without significantly impacting expression, solubility, or binding to cognate antigens. None of the noncanonical disulfide linkages identified were present in the natural human V_H_ repertoire. These data reveal an unexpected permissiveness of Ig V domains to noncanonical disulfide linkages at diverse locations in FRs, absent in the human repertoire, whose presence is compatible with antigen recognition and improves domain stability. Our work represents the most complete assessment to date of the role of engineered noncanonical disulfide bonding within FRs in Ig V domain structure and function.

Intradomain and interdomain disulfide linkages play critical roles in the folding and stability of antibodies and other large secreted macromolecules (1). Depending on isotype, human immunoglobulin (Ig) molecules contain between 14 and 23 disulfide linkages, including a highly conserved intradomain disulfide linkage connecting β-strands B and F of the two opposing β-sheets at the core of each Ig domain (2). Incorporation of exogenous disulfide linkages into proteins can enhance their thermodynamic and kinetic stability (3), which, for therapeutic antibodies, is associated with improved manufacturability, safety, and efficacy (4).

Incorporation of engineered disulfide linkages spanning β-strands A and G (Cys4-Cys119 and Cys6-Cys119; the international ImMunoGeneTics information system (IMGT) numbering used throughout) improved the thermostability of a human second Ig constant (C) domain of the IgG heavy chain (C_H_2) (5). In Ig variable (V) domains, which form the antigen-binding sites of antibodies, the canonical β-strand B–F intradomain disulfide linkage is formed between Cys23 and Cys104. Rarely, Ig V domains contain additional noncanonical disulfide linkages formed between Cys pairs located in framework regions (FRs): naturally occurring noncanonical linkages have been identified spanning Cys54-Cys78 (β-strands C'-D; (6, 7)) and Cys40-Cys55 (β-strands C-C'; (8, 9)), and a noncanonical linkage was successfully engineered between Cys39 and Cys87 (β-strands C-E) based on structural modeling (7). The Ig V domains of some vertebrates (*e.g.*, camelids; chickens; cows; sharks) contain noncanonical disulfide linkages at much higher frequencies (10), but these almost exclusively involve at least one Cys located in a complementarity-determining region (CDR). Incorporation of one or more noncanonical disulfide linkages into the FRs of single or multiple Ig domains can additively improve the thermostability of both the individual domains and the whole IgG molecules containing them (11, 12). Similar stabilizing effects have been observed for noncanonical disulfide linkages formed between positions within, or structurally analogous to, the CDR loops (13, 14), but these cannot be tolerated in all Ig V domains without compromising antigen binding.

Based on the serendipitous discovery of noncanonical disulfide linkages within Ig V domains in the past and an increasing acknowledgement of their importance in structural diversification of Ig repertoires (15, 16), we hypothesized that formation of such linkages at additional locations within Ig V domain FRs (Fig. 1*A*) would be compatible with antigen binding. To investigate this possibility, we applied an unbiased library approach to identify Ig V domain FR positions permissive for noncanonical disulfide linkage formation. To our surprise, we discovered that diverse intra- and inter-β-sheet disulfide linkages that are absent in the natural human repertoire could be accommodated within Ig V domain FRs, improving the stability of these domains without compromising antigen binding.

**Figure 1:**
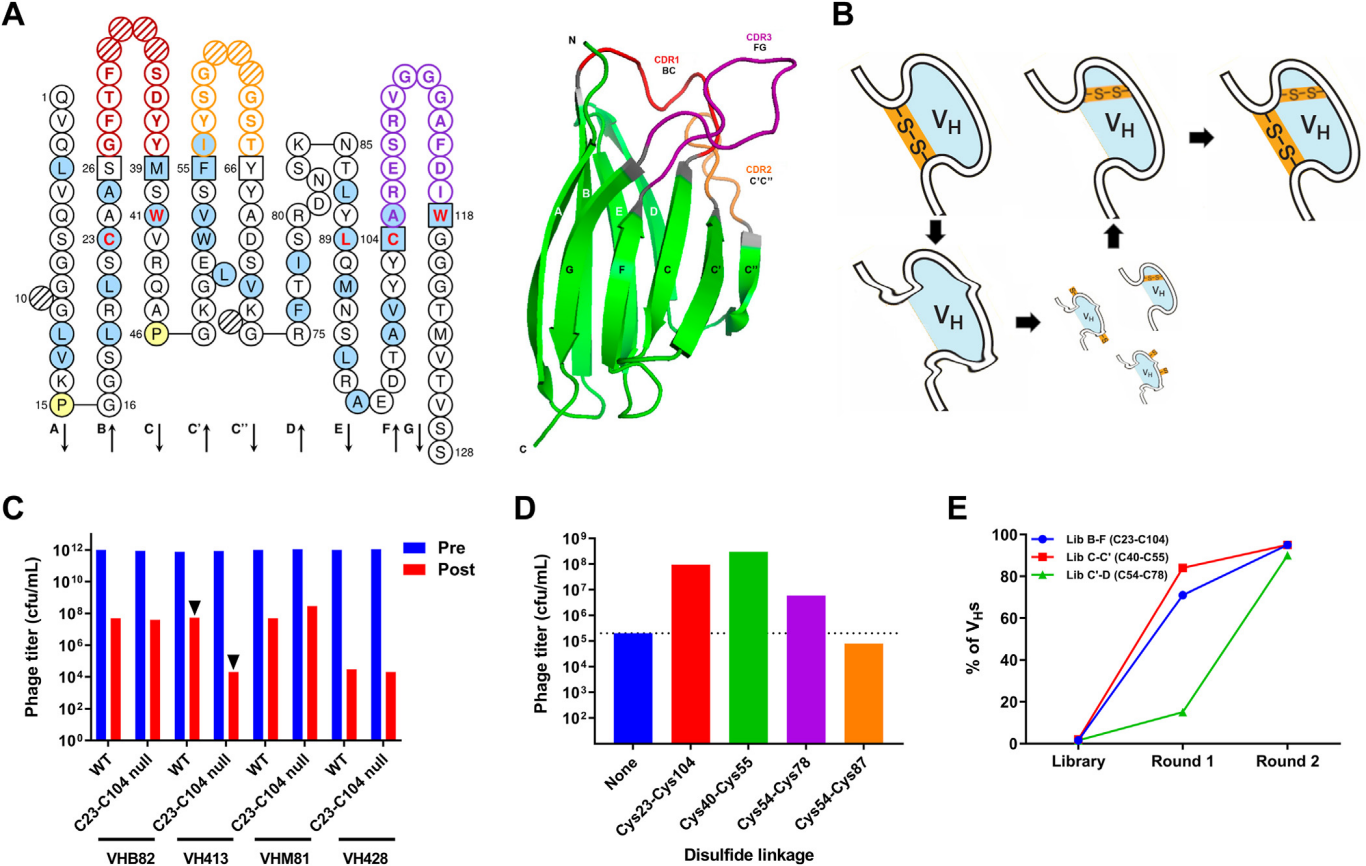
**Development of a model system for identification of stabilizing noncanonical disulfide linkages in Ig V domains.***A*, secondary and tertiary structure of immunoglobulin V domains. *Blue shading*, hydrophobic residue in ≥50% of V domains; *red bold*, highly conserved residues in V domains; *square*, complementarity-determining region (CDR) anchor position; *hashed*, gaps according to IMGT numbering; *yellow*, proline. CDR1, CDR2, and CDR3 are shown in *red*, *orange*, and *purple*, respectively. β-strands of the two opposing β-sheets are labeled in the tertiary structure in *black* and *white*. *Left*, figure produced using the sequence of VH413 with IMGT/Collier-de-Perles. *Right*, V_H_ domain of murine anti-lipoprotein A antibody 2E8 (PDB ID: 12E8). *B*, cartoon representation showing rescue of a destabilized Cys23-Cys104 null immunoglobulin V_H_ domain by Cys pair scanning of FRs, followed by enrichment of phage-displayed V_H_s bearing novel stabilizing intradomain noncanonical disulfide linkages from phage libraries using protein A selection. *C*, panning of fd phage displaying V_H_s (WT) or their Cys23-Cys104 null derivatives against protein A. Only VH413^C23-C104 null^ phage showed a significant difference in postselection output phage titer compared with the WT V_H_–displaying phage (*arrow heads*). Pre, preselection; Post, postselection. *D*, effect of introducing known canonical (Cys23-Cys104) and noncanonical (Cys40-Cys55 and Cys54-Cys78) disulfide linkages into VH413^C23-C104 null^ fd phage on output phage titer following protein A selection. “None” refers to unmodified VH413^C23-C104 null^ displaying fd phage (*blue bar* and *dotted line*). The effects of a negative control Cys pair (Cys54-Cys87) were also assessed. *E*, panning of test VH413^C23-C104 null^ Cys pair scan libraries (β-strands B–F, C–C', and C'–D) on protein A. The percent frequencies of known noncanonical stabilizing disulfide linkages formed between these β-strands is indicated. Four rounds of panning were performed. The pannings in panels *C*, *D*, and *E* were performed at room temperature with no heating step. “Library” denotes the unpanned libraries. cfu, colony-forming units; FR, framework region; V_H_, variable heavy chain; WT, wild-type.

## Results

### Selection of V_H_ domain for Cys pair scanning

To identify novel noncanonical disulfide linkages within Ig V domains, we first needed to develop a model system in which domain folding was perturbed by abrogation of the canonical Cys23-Cys104 disulfide linkage (Cys23-Cys104 null) and could be rescued by the stabilizing effects of noncanonical disulfide linkage formation elsewhere in the domain (Fig. 1*B*). Importantly, restoration of domain stability must be associated with a phenotype selectable by panning of phage-displayed libraries of Ig V domains such as reactivity with bacterial Ig-binding proteins (*e.g.*, protein A). Variable heavy chain (V_H_) domains were deemed suitable for this purpose as protein A recognizes a conformational epitope on V_H_ domains formed from at least four different β-strands in FR1 and FR3 (17).

We chose four different V_H_s (VHB82, VH413, VHM81, and VH428), representing a variety of germline VDJ rearrangements, from a set of well-characterized V_H_s we had at our disposal (18, 19). We then generated fd phage displaying each V_H_ and its Cys23-Cys104 null derivative (*i.e.*, Cys23Ala/Cys104Ala mutant). Following selection for binding to protein A at room temperature, phage displaying the Cys23-Cys104 null variants of two of the V_H_s (VHB82 and VHM81) had equivalent titers to those displaying the wild-type (WT) V_H_s, indicating no loss of protein A binding in the destabilized V_H_s. Surprisingly, both the WT and Cys23-Cys104 null variant of VH428 was inefficiently selected by panning on protein A. However, the output titers of VH413-displaying phage were 3 to 4 logs higher than those of VH413^C23-C104 null^-displaying phage following protein A selection (Fig. 1*C*), reflecting a loss of stability and folding in the destabilized V_H_. Introduction of the well-characterized Cys54-Cys87 and Cys40-Cys55 noncanonical disulfide linkages, but not a negative control Cys pair at locations nonpermissive to disulfide linkage formation (Cys54 and Cys78), into VH413^C23-C104 null^ restored the titers of phage displaying this V_H_ eluted from protein A selections to near-WT levels (Fig. 1*D*).

As proof-of-concept that VH413^C23-C104 null^ could be used to identify novel noncanonical disulfide linkages in Ig V domains, we first constructed three test Cys pair scan libraries of phage-displayed VH413^C23-C104 null^ domains bearing all possible combinations of Cys pairs spanning β-strands B–F, C–C', and C'–D. Panning of the libraries on protein A at room temperature rapidly enriched for VH413^C23-C104 null^ domains bearing canonical and previously identified stabilizing noncanonical disulfide linkages spanning Cys23-Cys104 (B–F), Cys40-Cys55 (C–C'), and Cys54-Cys78 (C'–D) (Fig. 1*E*). Efficient selection of stabilized V_H_s from the test libraries confirmed that introduction of both canonical and noncanonical disulfide linkages could rescue folding of the VH413^C23-C104 null^ domain and validated this strategy for the discovery of novel noncanonical disulfide linkages.

### Design and screening of V_H_ Cys pair scan libraries

**Figure 2:**
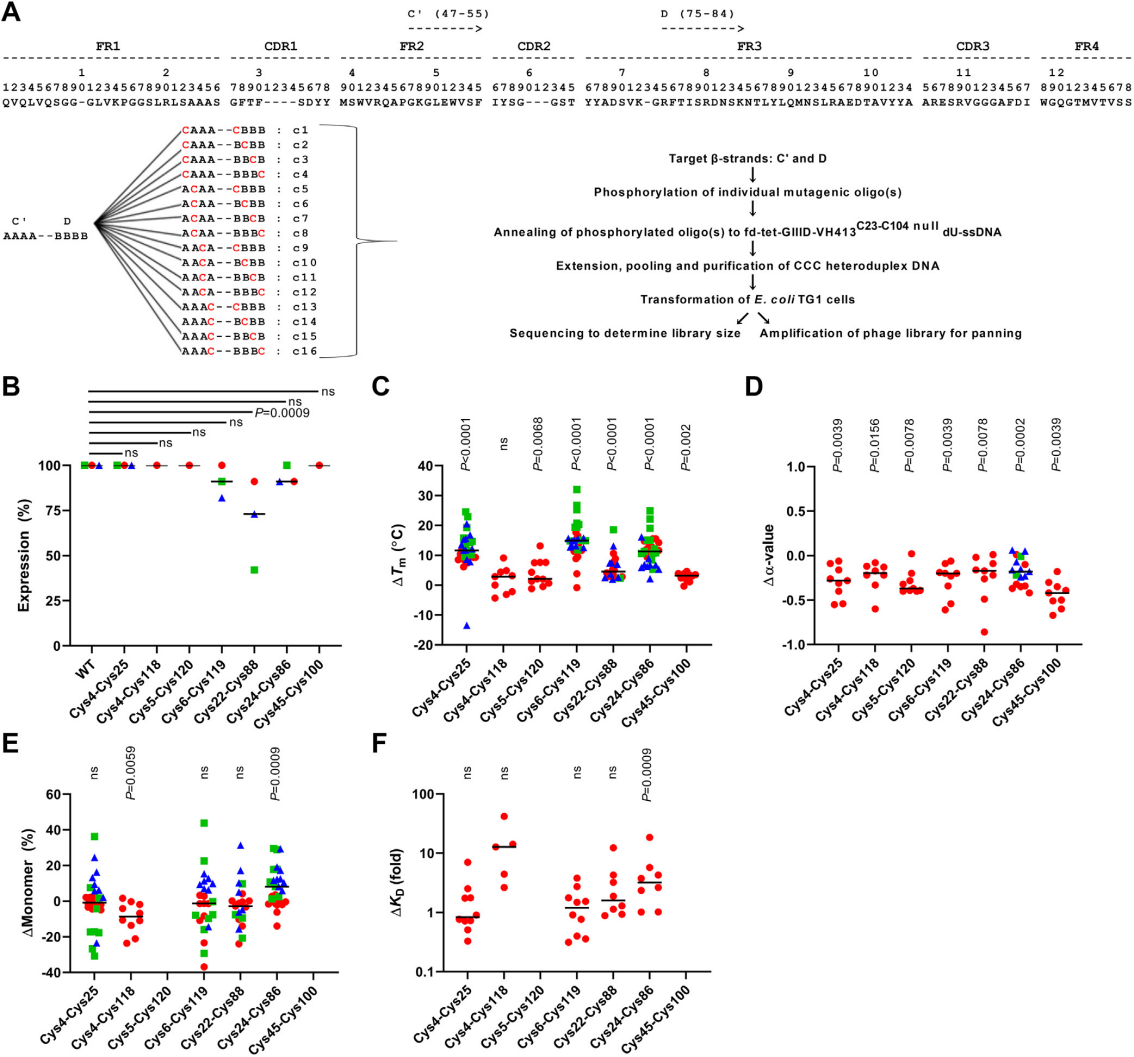
**Strategy for generation of Cys pair scan V_H_ libraries and effects of novel noncanonical disulfide linkages on Ig V domain biophysical properties.***A*, schematic of phage-displayed VH413^C23-C104 null^ Cys pair scan library construction. The example illustrates the construction of the C'–D library (only 16 representative Cys pair combinations, c1-c16, are pictured from the full set of 90 theoretical pairs). The *letter A* denotes amino acid positions in β-strand C', and the *letter B* denotes amino acid positions in β-strand D', while *red letter C* denotes positions mutated to Cys. Mutagenic oligonucleotides targeting two positions on two different β-strands were phosphorylated, annealed (either two oligonucleotides targeting separate β-strands or a single oligonucleotide spanning two contiguous β-strands in the primary amino acid sequence) to fd-tet GIIID-VH413^C23-C104 null^ dU-ssDNA, and extended. Heteroduplex DNA for all mutagenesis reactions targeting β-strands C'–D was pooled and used to transform *Escherichia coli* TG1 cells. The same steps were used for the construction of the remaining 16 Cys pair scan libraries, except that different mutagenic oligonucleotide pairs were used. *B*–*F*, effect of introducing seven Cys pairs forming putative noncanonical FR disulfide linkages on Ig V domain thermostability, other biophysical properties and antigen recognition. V_H_Hs are shown in *red circles*, V_H_s in *green squares*, and V_L_s in *blue triangles*. *B*, expression yields of Ig V domains bearing Cys pairs forming putative noncanonical FR disulfide linkages. Expression (%) refers to the percentage of sdAbs tested that expressed in adequate yields for subsequent experiments. Changes in (*C*) sdAb *T*_m_ and (*D*) fraction refolded (α-value) following introduction of FR Cys pairs were measured by CD. *E*, changes in sdAb monomericity following introduction of FR Cys pairs were measured by SEC-MALS. *F*, changes in the *K*_D_s of antigen-specific V_H_Hs following introduction of FR Cys pairs were assessed by SPR. For changes (Δ) in biophysical properties, values above the null (ΔMonomer 0%, Δ*T*_m_ 0 °C, and Δα-value 0) indicate improvement, while for changes in binding affinity, values above the null (Δ*K*_D_ 1) reflect weaker binding. Median values are indicated by *black lines*. *p*-values are from Fisher’s exact test (*B*) or Wilcoxon matched–pairs signed-rank test (*C*–*F*) comparing WT to Cys-engineered sdAbs. CD, circular dichroism; dU-ssdDNA, dUTP-containing fd-tetGIIID-VH413^C23-C104 null^ ssDNA; FR, framework region; Ig, immunoglobulin; ns, not significant; sdAb, single-domain antibody; SEC-MALS, size-exclusion chromatography-multiangle light scattering; SPR, surface plasmon resonance; V, variable; V_H_, variable heavy chain; V_H_H, variable heavy chain of camelid heavy chain–only antibody; V_L_, variable light chain; WT, wild-type.

To identify novel Ig V domain disulfide linkages without *a priori* assumptions of their locations, we constructed an additional 14 phage–displayed VH413^C23-C104 null^ libraries (distinct from the three test libraries described above; 17 libraries in total), each comprising mutant V_H_s bearing all possible combinations of Cys pairs in adjoining or opposing β-strands (Fig. 2*A*). In seven libraries the V_H_s bore Cys pairs on adjoining β-strands that were putatively capable of forming intra-β-sheet disulfide linkages, and in ten libraries the V_H_s bore Cys pairs on opposing β-strands that were putatively capable of forming inter-β-sheet disulfide linkages (Table 1). Sequencing of the libraries revealed that an average of 70% of clones displayed V_H_s mutagenized as intended, while 14% displayed V_H_s bearing a single unpaired Cys and 16% displayed unmodified VH413^C23-C104 null^ (Fig. S1, *A* and *B*). Comparison of library size with theoretical diversity limits indicated that each Cys pair was expected to be present multiple times in all 17 libraries (Table 1). Cys pairs in the libraries were generally evenly distributed across all targeted positions (*i.e.*, all mutagenesis reactions appeared to occur with similar efficiency), except for library C''–D, in which Cys residues at the N-terminus of β-strand C'' and the C-terminus of β-strand D predominated (Fig. S1, *C* and *D*).

**Table 1:** Diversity of 17 Cys pair scan phage-displayed V_H_ libraries

Inter-β-sheet library	IMGT positions targeted	Theoretical diversity^a^	Library size^b^	Intra-β-sheet library	IMGT positions targeted	Theoretical diversity^a^	Library size^b^
A–F	1–9, 11–15 (A)	112	5.1 × 10^3^	A–B	1–9, 11–15 (A)	154	1.9 × 10^4^
	97–104 (F)				16–26 (B)		
A–G	1–9, 11–15 (A)	154	4.5 × 10^3^	B–E	16–26 (B)	132	1.8 × 10^4^
	118–128 (G)				85–96 (E)		
B–C	16–26 (B)	88	6.3 × 10^3^	C–C'	39–46 (C)	72	1.9 × 10^4^
	39–46 (C)				47–55 (C')		
B–F	16–26 (B)	88	1.9 × 10^4^	C–F	39–46 (C)	64	4.6 × 10^3^
	97–104 (F)				97–104 (F)		
C–D	39–46 (C)	80	1.8 × 10^4^	C'–C"	47–55 (C')	72	6.4 × 10^3^
	75–84 (D)				66–72, 74 (C'')		
C–E	39–46 (C)	96	8.3 × 10^3^	D–E	75–84 (D)	120	1.6 × 10^4^
	85–96 (E)				85–96 (E)		
C'–D	47–55 (C')	90	3.2 × 10^3^	F–G	97–104 (F)	88	8.3 × 10^3^
	75–84 (D)				118–128 (G)		
C'–E	47–55 (C')	108	9.5 × 10^3^				
	85–96 (E)						
C''–D	66–72, 74 (C'')	80	3.9 × 10^3^				
	75–84 (D)						
E–F	85–96 (E)	96	8.5 × 10^3^				
	97–104 (F)						

Abbreviation: V_H_, variable heavy chain.

a Total number of Cys pair combinations calculated based on the number of residues present on the β-strands.

b Calculated as colony-forming units × % of clones correctly mutagenized.

The three test libraries described above (B–F, C–C', and C'–D) were not included in library pannings to prevent dominant selection of V_H_s bearing the canonical Cys23-Cys104 disulfide linkage and the previously identified Cys54-Cys78 and Cys40-Cys55 stabilizing noncanonical disulfide linkages. The remaining six intra-β-sheet libraries were pooled, as were the eight inter-β-sheet libraries, and panned separately on protein A. Panning of the six intra-β-sheet libraries was conducted both at room temperature and with a 30 min phage incubation at 50 °C at the beginning of each round, while panning of the eight inter-β-sheet libraries was conducted only with the additional heating step. Sequencing of clones identified from the second, third, and fourth rounds of both pannings revealed enrichment of V_H_s bearing five putative intra-β-sheet disulfide linkages (Cys4-Cys25, Cys19-Cys91, Cys22-Cys88, Cys24-Cys86, and Cys45-Cys100) and three putative inter-β-sheet disulfide linkages (Cys4-Cys118, Cys5-Cys120, and Cys6-Cys119). In early panning rounds, V_H_s bearing Cys pairs at other positions were identified at low frequencies, but in many cases disulfide linkage formation was structurally implausible (Tables S1 and S2).

### Effects of novel disulfide linkages on Ig V domain biophysical properties

As an initial screen, we first assessed the effects of introducing the eight putative noncanonical disulfide linkages, along with several negative control Cys pairs predicted not to form disulfide linkages, on the biophysical properties of two V_H_s (VH413 and VH428) and their Cys23-Cys104 null variants as well as two variable heavy chains of camelid heavy chain–only antibody (V_H_Hs) (A4.2 and A26.8; 20). VH413^C23-C104 null^ and VH428^C23-C104 null^ could not be expressed in significant quantities, but introduction of Cys24-Cys86 and, surprisingly, the negative control Cys pair Cys47-Cys72, resulted in low expression of V_H_s with melting temperatures (*T*_m_s) between 40 and 50 °C (data not shown). In this initial screen, introduction of seven of eight putative noncanonical disulfide linkages into V_H_s and V_H_Hs bearing the canonical Cys23-Cys104 disulfide linkage resulted in *T*_m_ increases ranging from 1.5 to 28 °C by differential scanning fluorimetry (Fig. S2). Exceptions included Cys19-Cys91, which resulted in no expression or decreased *T*_m_ in all V_H_s and V_H_Hs, and Cys5-Cys120, which resulted in decreased *T*_m_ for one V_H_. As expected, negative control Cys pairs generally resulted in loss of V_H_/V_H_H expression or decreased *T*_m_.

Based on these results, seven putative noncanonical disulfide linkages (Cys4-Cys25, Cys4-Cys118, Cys5-Cys120, Cys6-Cys119, Cys22-Cys88, Cys24-Cys86, and Cys45-Cys100) were introduced into a larger set of 12 V_H_s, 11 variable light chains (V_L_s), and/or 11 V_H_Hs. Three of the putative disulfide linkages (Cys4-Cys118, Cys5-Cys120, and Cys45-Cys100) were introduced into only the set of 11 V_H_Hs, while the other four putative linkages were introduced into all of the domains. The V_H_s and V_L_s were nonantigen-specific (18, 19, 21), while the V_H_Hs targeted *Clostridioides difficile* toxins A and B (20, 22), epidermal growth factor receptor (EGFR) (23), and insulin-like growth factor 1 receptor (IGF1R) (24). Introduction of all seven putative noncanonical disulfide linkages was generally well tolerated by most Ig V domains, except for Cys22-Cys88 which was successfully introduced into 10/11 V_H_Hs but only 8/11 V_L_s and 5/12 V_H_s with adequate expression yields for further experiments (Fig. 2*B* and Table S3). Three of the putative noncanonical disulfide linkages had only minor effects on *T*_m_ (Cys4-Cys118, Cys5-Cys120, Cys45-Cys100: average Δ*T*_m_ 1.5 °C, 4.2 °C, and 2.7 °C, respectively by circular dichroism (CD); Fig. 2*C* and Table S3), in line with thermostability changes associated with FR mutation alone rather than disulfide linkage formation (25), and were not investigated further. By contrast, introduction of putative noncanonical disulfide linkages formed between Cys4-Cys25, Cys6-Cys119, and Cys24-Cys86 resulted in average increases in Ig V domain *T*_m_ of 12 to 15 °C by CD, while introduction of the putative Cys22-Cys88 noncanonical disulfide linkage conferred an intermediate phenotype, increasing Ig V domain *T*_m_ by an average of 6.1 °C with high variability (Fig. 2*C* and Table S3). All four of these putative disulfide linkages generally conferred larger thermostability enhancements on V_H_s than V_H_Hs or V_L_s. Increased thermostability conferred by these four putative disulfide linkages was achieved at the cost of variable but generally modest decreases in the thermal refolding efficiency (α-value; Fig. 2*D* and Table S3) and monomericity of V_H_Hs (Fig. 2*E* and Table S3), while the impacts on solubility and refolding of V_H_ and V_L_ domains were more variable and less clear. The binding affinities of most antigen-specific V_H_Hs were unaffected, or only slightly weakened (Δ*K*_D_ generally <5-fold), by introduction of the four putative noncanonical disulfide linkages (Fig. 2*F*, Table S3 and Fig. S3); Cys24-Cys86 appeared to have the largest detrimental impact on binding affinity (median Δ*K*_D_: 4-fold). Introduction of the putative noncanonical disulfide linkages did not appear to negatively impact protein A binding (for V_H_Hs and V_H_s) or protein L binding (for V_L_s; data not shown).

### Effects of novel disulfide linkages on Ig V domain protease resistance

**Figure 3:**
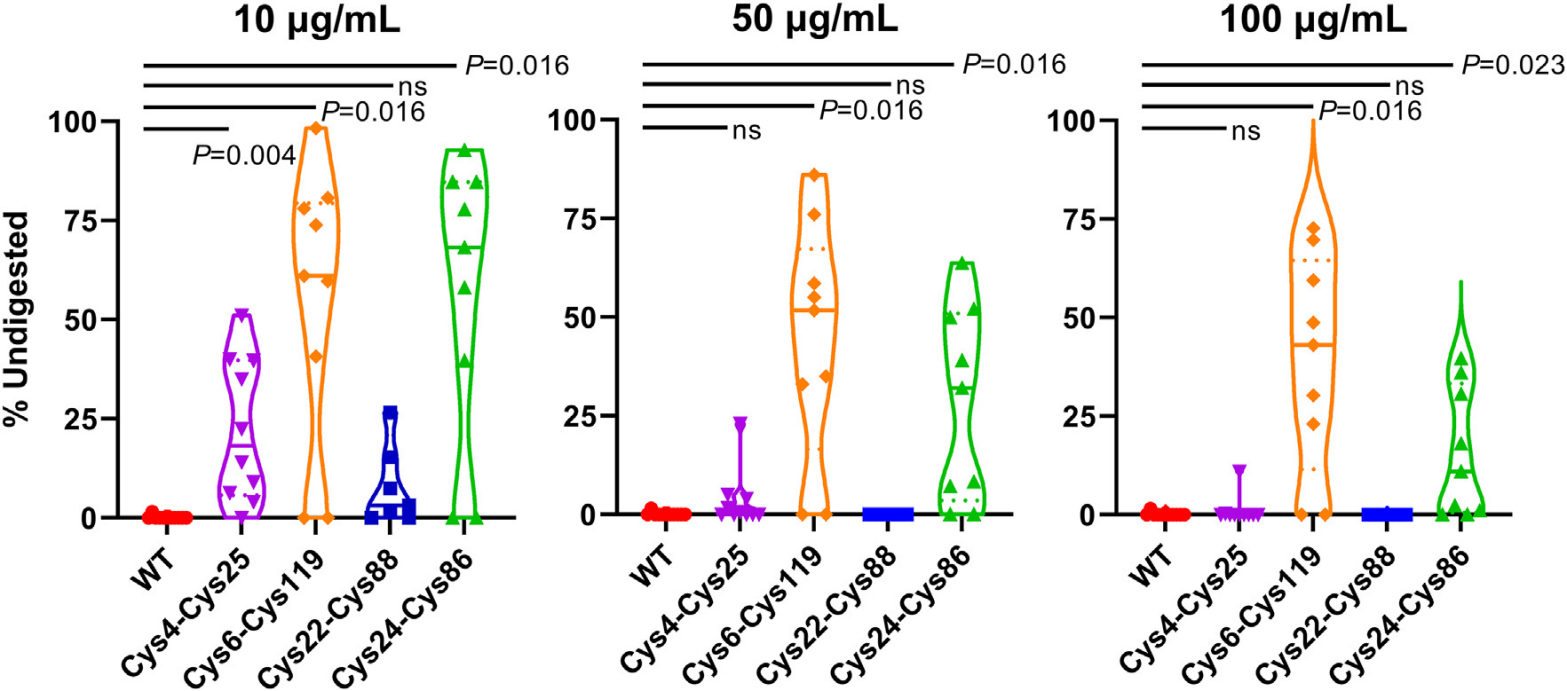
**Effect of introducing Cys pairs forming putative noncanonical FR disulfide linkages on V**_**H**_**H pepsin resistance.** The three Cys pairs conferring the highest increase in sdAb *T*_m_ (Cys4-Cys25, Cys6-Cys119, and Cys24-Cys86) were assessed as well as one Cys pair (Cys22-Cys88) that had an intermediate effect on *T*_m_. The V_H_Hs were digested with three concentrations of pepsin (10, 50 and 100 µg/ml) for 1 h at 37 °C, and then the remaining undigested V_H_H was quantitated by SDS-PAGE and band densitometry. *Solid lines* indicate medians and *dashed lines* indicate quartiles. The contours of the *violin plots* show probability density. *p*-values are from Wilcoxon matched–pairs signed-rank test comparing WT to Cys-engineered V_H_Hs. FR, framework region; ns, not significant; sdAb, single-domain antibody; V_H_H, variable heavy chain of camelid heavy chain–only antibody; WT, wild-type.

We previously found that introduction of a noncanonical disulfide linkage formed between Cys54-Cys78 increased the pepsin resistance of V_H_Hs (26) and V_L_s (21). To assess whether any of the putative noncanonical disulfide linkages identified in this study had similar effects, we tested the pepsin, trypsin, and chymotrypsin resistance of WT V_H_Hs and engineered variants bearing noncanonical disulfide linkages formed between Cys4-Cys25, Cys6-Cys119, Cys22-Cys88, and Cys24-Cys86. As observed previously for the Cys54-Cys78 linkage, the Cys6-Cys119 and Cys24-Cys86 noncanonical disulfide linkages significantly increased the pepsin resistance of the majority of V_H_Hs tested (Fig. 3 and Table S4). By contrast, the putative Cys4-Cys25 and Cys22-Cys88 linkages only modestly increased resistance to low concentrations of pepsin. None of the four noncanonical disulfide linkages had any effect on trypsin or chymotrypsin resistance (data not shown).

### Confirmation of the presence of noncanonical disulfide linkages by mass spectrometry

**Figure 4:**
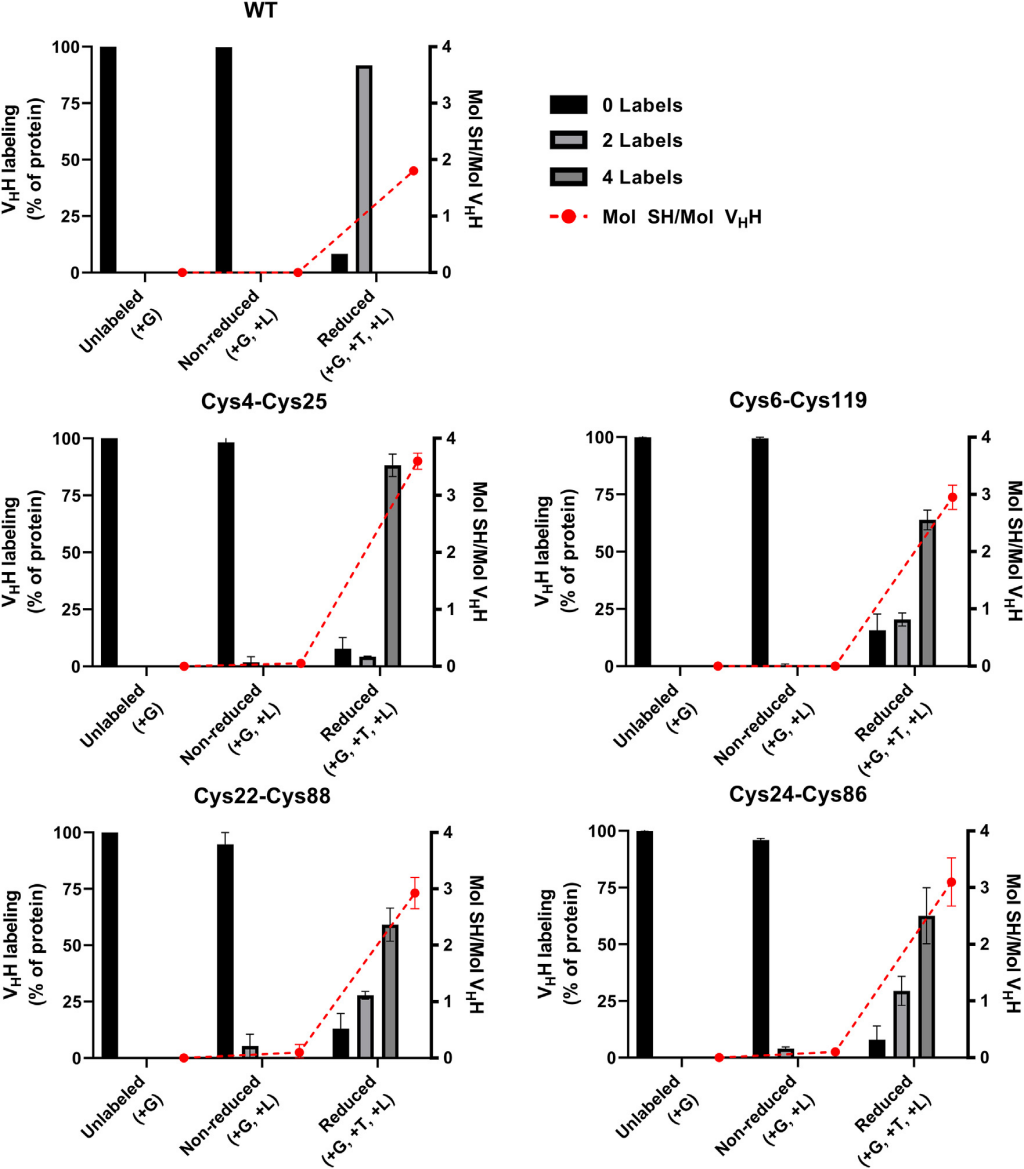
**LC-MS analysis of free sulfhydryl abundance in WT and Cys-engineered V**_**H**_**Hs.** For each category of V_H_H (WT V_H_Hs or V_H_Hs bearing the indicated engineered Cys pair), the percentage of unlabeled, reduced, and nonreduced protein analyzed bearing 0, 2, and 4 maleimide-PEG2-biotin labels is indicated (*left y*-axis; sum: 100%). The molar ratio of free sulfhydryl (SH) groups to total V_H_H protein is shown in *red* (*right y*-axis). Bars represent means and error bars represent SEMs. G, GdnHCl, guanidine hydrochloride; L, label (maleimide-PEG2-biotin); LC-MS, liquid chromatography-mass spectrometry; T, TCEP, tris(2-carboxyethyl)phosphine; V_H_H, variable heavy chain of camelid heavy chain–only antibody; WT, wild-type.

Disulfide linkage formation in Cys-engineered Ig V domains was indirectly evaluated by measuring the abundance of free sulfhydryl groups with liquid chromatography-mass spectrometry (LC-MS). WT and Cys-engineered V_H_Hs bearing noncanonical disulfide linkages formed between Cys4-Cys25, Cys6-Cys119, Cys22-Cys88, and Cys24-Cys86 were denatured in 6 M guanidine hydrochloride (GdnHCl), reduced with 5 mM tris(2-carboxyethyl)phosphine (TCEP) and then labeled with a 150-fold molar excess of maleimide-PEG2-biotin (mPEG2-biotin). Nonreduced (GdnHCl + mPEG2-biotin) and unlabeled (GdnHCl only) proteins served as controls. Intact LC-MS analysis confirmed the presence of all four noncanonical disulfide linkages in Ig V domains, as shown by incorporation of only two mPEG2-biotin labels in a WT V_H_H bearing only the canonical Cys23-Cys104 disulfide linkage and up to four labels in all of the Cys-engineered V_H_Hs following TCEP reduction (Fig. 4 and Table S5). As observed previously for the Cys40-Cys55 and Cys54-Cys78 noncanonical disulfide linkages, there was some evidence that the four novel noncanonical disulfide linkages were formed heterogeneously following introduction into V_H_Hs bearing the canonical Cys23-Cys104 linkage. Unlike formation of the canonical Cys23-Cys104 linkage in a WT V_H_H or formation of the Cys4-Cys25 and Cys6-Cys119 noncanonical disulfide linkages, minor proportions of unreduced V_H_Hs bearing the Cys22-Cys88 and Cys24-Cys86 linkages (1.0–12.7%) bore two free sulfhydryl groups, indicating the presence of unpaired Cys residues. Moreover, based on the molar ratio of free sulfhydryl groups to total Ig V domain protein, reduction of the canonical Cys23-Cys104 disulfide linkage in a WT V_H_H, as well as reduction of the noncanonical Cys4-Cys25 disulfide linkage in Cys-engineered V_H_Hs, was ∼90% efficient, whereas the Cys6-Cys119, Cys22-Cys88, and Cys24-Cys86 noncanonical disulfide linkages were not fully reduced in a subpopulation of V_H_H molecules under the conditions tested.

### Presence of novel noncanonical disulfide linkages in human Ig V_H_ domain repertoires

**Figure 5:**
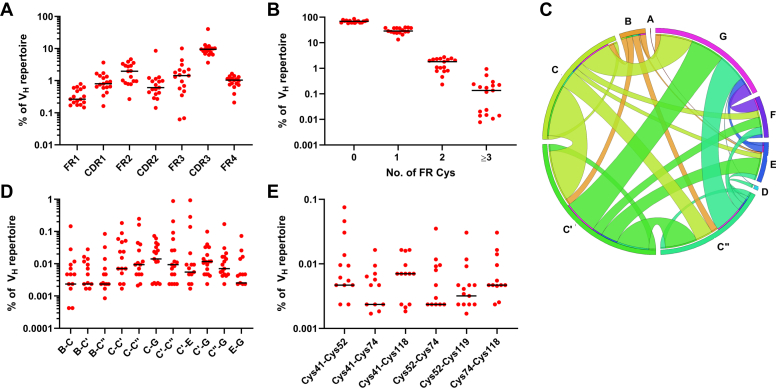
**Presence of noncanonical Cys residues in the human expressed V**_**H**_**repertoires of 17 individuals.** The canonical intradomain disulfide linkage formed by Cys residues at IMGT positions 23 and 104 was disregarded in all analyses presented. *A*, frequency of noncanonical Cys residues in V_H_ FRs and CDRs. *B*, frequency of V_H_s in the expressed repertoire bearing the indicated number of Cys residues. *C*, *Circos plot* showing the relative frequencies and β-strand locations of noncanonical Cys pairs in the subset of expressed human V_H_s bearing ≧4 FR Cys residues. The sizes of β-strands along the circumference of the *circle* reflect the frequency of noncanonical Cys, and links show the relative frequencies of the locations of the second Cys in the pair. *D*, most frequent β-strands bearing noncanonical Cys pairs among human V_H_s bearing ≧4 FR Cys residues. *E*, most frequent noncanonical Cys pairs among human V_H_s bearing ≧4 FR Cys residues. *Black lines* represent median values across 17 V_H_ repertoires. Note that *y*-axes are log-scaled and zero values are not plotted. CDR, complementarity-determining region; FR, framework region; V_H_, variable heavy chain.

To determine whether the noncanonical disulfide linkages identified in this study were present in the human antibody repertoire, we used Illumina MiSeq sequencing to interrogate the peripheral blood resting memory B-cell V_H_ repertoires of 17 individuals to an average depth of 10^5^ reads per individual (Table S6). Cys residues in Ig V_H_ domains were most common in CDR3 (Fig. 5*A*), and V_H_s bearing an additional noncanonical Cys pair located in FRs (four Cys residues in total) were rare in the repertoire, representing <1% of all rearranged V_H_s (Fig. 5*B*). No V_H_ sequences were identified bearing putative noncanonical disulfide linkages formed between Cys4-Cys25, Cys6-Cys119, Cys22-Cys88, Cys24-Cys86, or any of the other positions identified in this study. Noncanonical Cys pairs in FRs were distributed widely across various V_H_ domain β-strands in locations unlikely to enable disulfide linkage formation due to their distance in three-dimensional space (Fig. 5, *C*–*E*). These results agree with those of a recent repertoire sequencing study showing that putative noncanonical disulfide linkages formed between Cys residues in FRs were also not detected (16). Instead, most noncanonical Cys residues in the human V_H_ repertoire lie in FR2 and FR3, where they may be able to form disulfide linkages with Cys residues in CDRs.

## Discussion

The roles of intradomain disulfide linkages in Ig V domains are poorly understood. Even the canonical Cys23-Cys104 linkage, present in all V_H_ and V_L_ domains, is often dispensable for antigen binding and domain stability (27). The roles of noncanonical disulfide linkages, which typically involve one or more Cys residue(s) in CDRs, are even less clear. Such linkages are often germline encoded, with at least one Cys located in V, D, or J gene segments, and are present at relatively high frequencies in the Ig V domain repertoires of several nonhuman vertebrates, where they may play roles in expanding paratope diversity (15, 16), structuring ultralong CDR-H3 loops (28), reducing entropic penalties for antigen binding (29), and stabilizing the Ig V domain fold under harsh biological conditions (30). Of the three previously described intradomain noncanonical Ig V disulfide linkages in FRs (Cys54-Cys78, Cys40-Cys55, and Cys39-Cys87), Cys54-Cys78 was the result of somatic hypermutation in a camelid V_H_H (7), Cys40-Cys55 is encoded in the germline of several rabbit IGHV genes (8) and Cys39-Cys87 does not occur naturally (7). Thus, noncanonical disulfide linkages in FRs appear to occur naturally at lower frequencies than inter-CDR loop disulfide linkages in natural antibody repertoires even though their impacts on Ig V domain biophysical properties can be dramatic.

Here, we explored the sequence tolerance of Ig V domains to the introduction of noncanonical disulfide linkages in FRs. We used a Cys pair scanning phage display library approach to identify such linkages (Fig. 1), reasoning that noncanonical disulfide linkages within FRs would be generalizable to a broader spectrum of Ig V domains than those formed between Cys residues in CDRs. This strategy, while simple and powerful, had several limitations. First, not all noncanonical disulfide linkages will necessarily enhance the folding or stability of individual Ig V domains, and such linkages would not be expected to be identified using the phage-based protein A selection applied in our study. Second, selection of phage-displayed V_H_s bearing stabilizing noncanonical disulfide linkages depended on multiple factors, including adequate expression of V_H_-pIII fusion proteins, binding of the phage-displayed V_H_ to protein A and preserved infectivity of V_H_-displaying phage following high-pH elution. Even for well-characterized V_H_s with similar protein A binding affinities, we have observed significant variation in the results of protein A selections of V_H_-displaying phage (unpublished results), and so some stabilizing noncanonical disulfide linkages may not have been recovered from our library screens. Third, we made several concessions to simplify panning of the libraries: (i) Cys pair scan libraries for β-strands B–F, C–C' or C'–D but excluding the known Cys23-Cys104, Cys40-Cys55, and Cys54-Cys78 linkages were not prepared, and thus other putative linkages formed between these β-strands would have been missed and (ii) we pooled the intra- and inter-β-sheet libraries for panning, potentially resulting in out-competition of some noncanonical linkages with low starting frequencies in the library or modest stabilizing effects. Fourth, several of the Cys pairs identified using this approach may not result in formation of noncanonical disulfide linkages in Ig V domains, or at least not in domains bearing the canonical Cys23-Cys104 linkage, as shown by their apparent inability to enhance *T*_m_ or protease resistance. Thus, subtle modifications to the FRs of the destabilized VH413^C23-C104 null^ scaffold *via* Cys substitution may have substantially affected the properties of this protein and the associated library selections without having any apparent effects following introduction into Ig V domains bearing canonical intradomain disulfide linkages. Finally, while the model system used here for identifying noncanonical disulfide linkages (rescue of Ig V domain folding and protein A binding) was effective, alternative selection strategies such as rescue of antigen binding or physicochemical stability may be envisaged and could yield different results.

Screening of the Cys pair scan V_H_ libraries identified seven novel Cys pairs putatively forming noncanonical disulfide linkages in FRs (inter-β-sheet: Cys4-Cys25, Cys22-Cys88, Cys24-Cys86, and Cys45-Cys100; intra-β-sheet: Cys4-Cys118, Cys5-Cys120, and Cys6-Cys119). Four of these seven putative noncanonical linkages (Cys4-Cys25, Cys6-Cys119, Cys22-Cys88, and Cys24-Cys86) showed significant enhancement of *T*_m_ and/or protease resistance, when incorporated into panels of single-domain antibodies (sdAbs) bearing the canonical Cys23-Cys104 disulfide linkage (Figs. 2 and 3). Both Cys4-Cys119 and Cys6-Cys119 noncanonical linkages were previously shown to improve the thermostability of Ig C domains (5), and although Cys4-Cys119 was not identified in our screens and we did not assess its impact on Ig V domain biophysical properties, we presume that this linkage can form in Ig V domains as well. Intriguingly, we identified other Cys pairs whose introduction was tolerated nearby on β-strands A–G (Cys4-Cys118 and Cys5-Cys120); although these had only marginal effects on *T*_m_, this may reflect a hotspot for disulfide linkage formation, and other combinations of Cys residues in this region may be capable of forming noncanonical disulfide linkages. Introduction of each of the seven Cys pairs identified in our screens into sdAbs was generally well tolerated, with minimal detrimental impact on expression yields of sdAbs and on antigen binding affinity of V_H_Hs. The effect of noncanonical disulfide linkage introduction on antigen binding by V_H_ and V_L_ domains was not studied as the V_H_ and V_L_ domains used in our study were nonantigen-specific. Noncanonical disulfide linkages that appeared to potentially disrupt antigen recognition included Cys4-Cys118, consistent with the location of the Trp118→Cys substitution at the base of the CDR3 loop, as well as Cys24-Cys86. Introduction of each of the seven Cys pairs individually also resulted in modest decreases in the monomericity and thermal refolding efficiency of V_H_Hs and more variable effects on the monomericity and thermal refolding of V_H_s and V_L_s. We caution that these parameters are much more variable for sometimes labile V_H_ and V_L_ domains, and that each of the noncanonical disulfide linkages identified in this study would most likely have similar effects with similar variability (depending on the particular linkage) in all types of Ig V domains. Although not yet tested experimentally, it is possible that thermostability improvements and preserved antigenic reactivity in Ig V domains following introduction of the noncanonical disulfide linkages identified in this study will carry over to larger molecules (*e.g.*, scFv, Fab, IgG) containing the engineered V domains, as demonstrated previously for the Cys54-Cys78 noncanonical linkage (11, 12).

The overarching goal of this study was to identify noncanonical disulfide linkages that could be used in protein engineering strategies to enhance the biophysical properties of Ig V domains already containing the canonical Cys23-Cys104 disulfide linkage. Several of our results underline the high degree of complexity in the protein phenotypes associated with disulfide linkage formation in Ig V domain FRs. First, there was no direct connection between the ability of a given Cys pair to rescue folding of the VH413^C23-C104 null^ scaffold and improvements in thermostability following introduction into Ig V domains bearing the canonical Cys23-Cys104 disulfide linkage (*e.g.*, for the Cys4-Cys118, Cys5-Cys120, and Cys45-Cys100 putative noncanonical linkages). We did not confirm whether these Cys pairs formed disulfide linkages using LC-MS in either the VH413^C23-C104 null^ scaffold or in Ig V domains bearing the Cys23-Cys104 canonical linkage. Second, introduction of the Cys4-Cys25, Cys6-Cys119, Cys22-Cys88, and Cys24-Cys86 noncanonical disulfide linkages had widely differing impacts on thermostability and pepsin resistance depending on the individual Ig V domain tested. Following introduction of the Cys22-Cys88 noncanonical disulfide linkage, approximately half of Ig V domains tested showed thermostability improvements of 2 to 4 °C, well below the average for previously described noncanonical disulfide linkages (9) and potentially in line with thermostability changes associated with FR mutations alone (25). However, the Cys22-Cys88 noncanonical disulfide linkage was confirmed to be present by LC-MS, even in two V_H_Hs (FC5 and A5.1) that showed relatively small thermostability improvements following its introduction. The data were thus not explained by formation of the novel noncanonical disulfide linkages only in selected Ig V domains nor by heterogeneity in the degree of linkage formation between molecules (Fig. 4). Third, thermostability improvements following introduction of noncanonical disulfide linkages were not always correlated with pepsin resistance: the presence of the Cys4-Cys25 and Cys6-Cys119 linkages had only a modest impact on V_H_H pepsin resistance despite a similar enhancement of *T*_m_ compared with the Cys22-Cys88 and Cys24-Cys86 linkages. Thus, despite strong correlations between thermostability improvements and pepsin resistance for some noncanonical disulfide linkages, including the previously described Cys54-Cys78 linkage (especially when *T*_m_ is measured at pH 2.0; (20, 26)), it appears that the thermodynamic properties of Ig V domains at high temperatures cannot be generally extrapolated to the much lower physiologic temperatures at which endopeptidase digestion occurs. Together, the data suggest that contextual effects of disulfide linkage location within the FRs of different Ig V domains may influence domain folding, solubility, thermostability, and protease resistance. Variation in the effects of noncanonical disulfide linkage formation on Ig V domain physicochemical properties is consistent with the relatively stringent spatial (Cα-Cα distance 4–7 Å and dihedral angle near 90°; (3, 31)) and chemical (local pH > p*K*_a_ of thiol groups and oxidizing environment; (1, 2, 3)) requirements for their formation, deviation from which can result in significantly reduced bond energy (32). Structural and biochemical diversity in Ig V domain FRs (33) may partially explain the absence of a straightforward relationship between noncanonical disulfide linkage formation and protein physicochemical properties.

Interrogation of the expressed human V_H_ repertoires of 17 individuals using next-generation DNA sequencing did not detect any of the seven putative noncanonical disulfide linkages identified from our screens at any appreciable frequency (Fig. 5). Among the rare V_H_s bearing two noncanonical Cys residues in FRs (<1% of the repertoire), Cys pairs were widely distributed across β-strands and three-dimensional space, suggesting that their role did not involve disulfide linkage formation with one another. These FR Cys residues may instead form disulfide linkages with Cys in CDRs or may simply reflect somatic hypermutation of FRs. Interestingly, Cys residues were identified at relatively high frequencies at the N terminus of β-strand G (positions 118/119), supporting the hypothesis that this region may be a hotspot for Cys residues and noncanonical disulfide linkage formation within Ig V domain FRs. A caveat to our repertoire sequencing studies was that noncanonical Cys residues could not be detected in the N terminus of β-strand A or the C terminus of β-strand G because these regions were primer-forced. Nevertheless, we conclude that as with the previously described Cys39-Cys87 linkage, the novel Ig V domain noncanonical disulfide linkages described here are not present in the human repertoire despite their ability to enhance domain biophysical properties.

**Figure 6:**
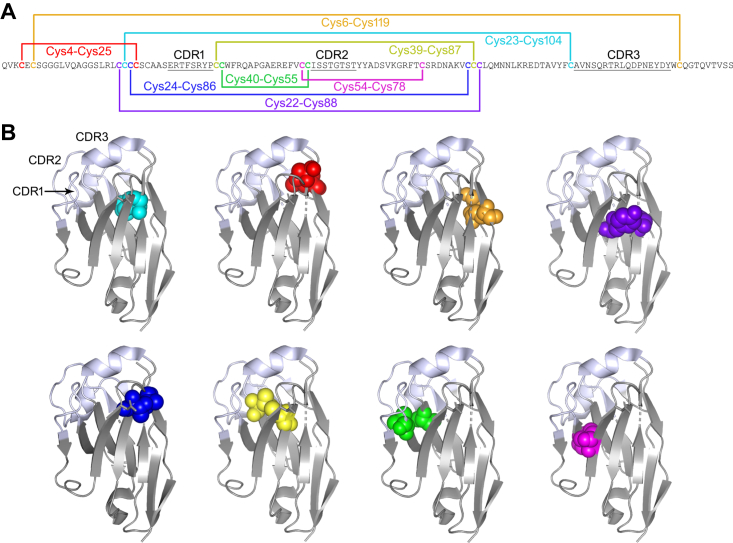
**Locations of known noncanonical intradomain disulfide linkages in Ig V domain FRs in a representative V**_**H**_**H.***A*, amino acid sequence of the *Clostridioides difficile* toxin A–specific V_H_H A26.8 (21) with the locations of the canonical Cys23-Cys104 disulfide linkage (*cyan*) and noncanonical disulfide linkages in Ig V domain FRs: Cys4-Cys25 (*red*), Cys6-Cys119 (*orange*), Cys22-Cys88 (*indigo*), Cys24-Cys86 (*blue*), Cys39-Cys87 (*yellow*), Cys40-Cys55 (*green*), and Cys54-Cys78 (*magenta*). The first four noncanonical disulfide linkages were identified in this study. CDRs are *underlined*. Note that while the positions of the noncanonical disulfide linkages are shown using IMGT numbering, IMGT gaps are not shown in the sequence of A26.8. Refer to Figure 1*A* for an example of IMGT numbering including gaps. *B*, crystal structure of A26.8 V_H_H (PDB ID: 4NBZ) with the locations of the canonical and noncanonical disulfide linkages colored as in *A*: canonical Cys23-Cys104 (*cyan*) and noncanonical Cys4-Cys25 (*red*), Cys6-Cys119 (*orange*), Cys22-Cys88 (*indigo*), Cys24-Cys86 (*blue*), Cys39-Cys87 (*yellow*), Cys40-Cys55 (*green*), and Cys54-Cys78 (*magenta*). CDRs are shown in *white* and FRs in *gray*. The first four noncanonical disulfide linkages were identified in this study. CDR, complementarity-determining region; FR, framework region; Ig, immunoglobulin; V_H_H, variable heavy chain of camelid heavy chain–only antibody.

In summary, we found that Ig V domain FRs were unexpectedly permissive to noncanonical disulfide linkages at multiple positions, absent in the human repertoire, whose presence was compatible with antigen recognition and improved domain thermostability, and in some cases, pepsin resistance. The results of this study more than double the previously known spectrum of noncanonical disulfide linkages discovered in Ig V domains, the majority of which were identified in naturally occurring antibodies (Fig. 6). Because individual Ig V domains may differ in their tolerance of specific noncanonical disulfide linkages, this expanding panel of linkages provides a useful toolbox for stability engineering of antibodies. Clearly, the strong conservation of the canonical Cys23-Cys104 linkage does not preclude formation of other noncanonical linkages in Ig V domain FRs. Our work also demonstrates the power of molecular engineering and library approaches to surpass the limitations of natural antibody repertoires.

## Experimental procedures

### Cloning of V_H_ genes into fd-tetGIIID

V_H_ coding regions were cloned into the fd-tetGIIID vector (34) using standard molecular biology techniques. DNA encoding Cys23-Cys104 null mutants of V_H_s was synthesized and cloned into pSJF2H by GenScript. Briefly, DNA encoding VHB82, VH413, VH428, and VHM81 and their Cys23-Cys104 null derivatives was PCR-amplified from the pSJF2H vector using primers that introduced 5′ *Apa*LI and 3′ *Not*I restriction sites (Table S7). The amplicons and fd-tetGIIID were double-digested with *Apa*LI and *Not*I (New England Biolabs) and purified from 1.2% (w/v) agarose gels using a QIAquick gel extraction kit (QIAGEN). Each insert (500 ng) was ligated with 100 ng of dephosphorylated fd-tetGIIID in 20-μl reactions containing 1 U T4 DNA ligase (Life Technologies) at 4 °C overnight. Electrocompetent *Escherichia coli* TG1 cells were transformed with the ligation mixtures by electroporation and plated on 2×YT agar plates containing 12.5 μg/ml tetracycline (Sigma). Following overnight incubation at 37 °C, single colonies harboring fd-tetGIIID vectors bearing V_H_ inserts were identified by colony PCR and DNA sequencing.

### Preparation of dUTP-containing fd-tetGIIID-VH413^C23-C104 null^ ssDNA

dUTP-containing fd-tetGIIID-VH413^C23-C104 null^ ssDNA (dU-ssdDNA) was prepared as previously described with modifications (35, 36). A single *E. coli* CJ236 (*dut*^*−*^*ung*^*−*^) colony was picked from a 2×YT agar plate containing 15 μg/ml chloramphenicol (Sigma) and used to inoculate a 5 ml 2×YT culture. The culture was grown at 37 °C with 240 rpm shaking until an absorbance at 600 nm (*A*_600_) of 0.5 was reached, then cells (0.1 ml) were infected with fd-tetGIIID-VH413^C23-C104 null^ phage (10^12^ colony-forming units/ml, 10 μl) for 30 min at 37 °C without shaking and plated on 2×YT agar plates containing 12.5 μg/ml tetracycline. Following overnight incubation at 37 °C, a 10-ml 2×YT culture containing tetracycline (12.5 μg/ml) and chloramphenicol (15 μg/ml) was inoculated with a single colony and grown at 37 °C with 240 rpm shaking until an *A*_600_ of 1 was reached. Two 0.5-l 2×YT cultures containing the same antibiotics and 0.25 μg/ml uridine (Sigma) were inoculated with the starter culture and grown at 37 °C with 240 rpm shaking. The next day, the cells were pelleted by centrifugation at 3000*g* at 4 °C for 15 min and phage were purified by PEG precipitation (37). Successful incorporation of uridine into phage ssDNA was verified by comparative titering of the phage on *E. coli* TG1 and CJ236 cells. Finally, dU-ssDNA was extracted using the QIAprep Spin M13 Kit (QIAGEN) and resuspended in water. DNA was quantitated *via* absorbance at 260 nm using a ND-2000 spectrophotometer (Thermo Fisher Scientific).

### Generation of fd-tetGIIID-VH413^C23-C104 null^ Cys mutants

fd-tetGIIID-VH413^C23-C104 null^ was engineered to bear noncanonical disulfide linkages formed between Cys54-Cys78 and Cys40-Cys55 using Kunkel mutagenesis as previously described with modifications (36, 37). In addition, a negative control Cys pair was introduced at locations tolerant to Cys residue introduction but that would not be expected to permit disulfide linkage formation (Cys54-Cys87). Briefly, 0.1 μg (∼10 pmol) of each mutagenic oligonucleotide (Table S8) was phosphorylated for 2 h at 37 °C in a 10-μl reaction containing 50 mM Tris–HCl, pH 7.5, 10 mM MgCl_2_, 5 mM DTT, 0.5 mM ATP, and 2.5 U T4 polynucleotide kinase (New England Biolabs). Subsequently, 20 μl of each phosphorylated oligonucleotide pair (10 μl of each oligonucleotide) was annealed with 330 ng of fd-tetGIIID-VH413^C23-C104 null^ dU-ssDNA in a 50-μl reaction whose temperature was cycled as follows: 90 °C, 3 min; 50 °C, 3 min; and 20 °C, 5 min. The annealed oligonucleotides were extended by adding 0.5 μl of 10 mM ATP, 1 μl of 10 mM deoxynucleotide triphosphates, 1 μl of 100 mM DTT, 2.5 U of T7 DNA polymerase (New England Biolabs), and 1.25 U of T4 DNA ligase (New England Biolabs) and letting covalently closed circular (CCC) heteroduplex DNA form at room temperature overnight. Formation of CCC heteroduplex DNA was verified by agarose gel electrophoresis. The reactions were purified using the QIAquick PCR purification kit (QIAGEN), and DNA was quantitated *via* absorbance at 260 nm using a ND-2000 spectrophotometer. Electrocompetent *E. coli* TG1 cells were transformed with the CCC heteroduplex DNA by electroporation and plated on 2×YT agar plates containing 12.5 μg/ml tetracycline. Following overnight incubation at 37 °C, single colonies harboring fd-tetGIIID vectors encoding V_H_s with the desired mutations were identified by DNA sequencing.

### Generation of Cys pair scan V_H_ libraries

The 17 Cys pair scan libraries of fd-tetGIIID-VH413^C23-C104 null^ were generated by Kunkel mutagenesis using oligonucleotide pairs targeting specific β-strands essentially as described above. The overall strategy is shown in Figure 2*A* and the sequences of all mutagenic oligonucleotides are listed in Table S9. For each library, the number of mutagenesis reactions was *a* × *b*, where *a* and *b* are the respective numbers of residues in the two β-strands being targeted. Mutagenic oligonucleotides (0.1 μg) were phosphorylated as described above, then phosphorylated oligonucleotides (either pairs of oligonucleotides each mutating one β-strand or a single oligonucleotide mutating two β-strands; 10 μl each) were annealed with 330 ng of fd-tetGIIID-VH413^C23-C104 null^ dU-ssDNA in 50-μl reactions prepared in 96-well plates (Fig. S4). T7 DNA polymerase and T4 DNA ligase were added to the reactions and the annealed oligonucleotides were extended overnight at room temperature to form CCC heteroduplex DNA. The individual CCC heteroduplex DNAs were pooled for each library and 25% of the total volume was purified using the QIAquick PCR purification kit and quantitated *via* absorbance at 260 nm using a ND-2000 spectrophotometer. Electrocompetent *E. coli* TG1 cells were transformed with the CCC heteroduplex DNA by electroporation (five transformations per library) and plated on 2×YT agar plates containing 12.5 μg/ml tetracycline. Following overnight incubation at 37 °C, 40 to 50 single colonies per library harboring fd-tetGIIID vectors encoding V_H_s were sequenced to determine the proportion correctly mutagenized and the functional library size.

### Panning of phage-displayed V_H_s and V_H_ libraries against protein A

To prepare fd phage displaying V_H_s and their Cys-engineered variants as well as Cys pair scan libraries of VH413^C23-C104 null^, *E. coli* TG1 cells transformed with the heteroduplex CCC DNAs described above were transferred to 100 ml 2×YT cultures containing tetracycline (12.5 μg/ml) and grown overnight at 37 °C with 240 rpm shaking. The next day, phage were purified by PEG precipitation and resuspended in 2 ml of PBS, pH 7.4. Phage concentrations were estimated spectrophotometrically using the formula: $\frac{virions}{mL}=\frac{\left(A_{269}-A_{320}\right)\times 10^{16}}{No.\;of\;bases\;in\;genome}$. Phage were aliquoted and stored at −20 °C. For panning, wells of NUNC MaxiSorp microtiter plates (Thermo Fisher Scientific) were coated overnight at 4 °C with 5 μg of protein A (Cat. No. 21184, Thermo Fisher Scientific) in 100 μl of PBS. The next day, wells were blocked with 300 μl of PBS containing 2% (w/v) skim milk at 37 °C for 2.5 h. Single V_H_–displaying phage clones or Cys pair scan libraries of phage-displayed V_H_s (10^12^ colony-forming units/ml, 100 μl in PBS containing 2% skim milk) were added to wells and incubated for 1.5 h. Pooled intra-β-sheet Cys pair scan libraries were panned in duplicate using either (i) phage prepared as above or (ii) phage heated at 50 °C for 30 min, allowed to cool for 20 min at room temperature, then centrifuged at 16,000*g* for 5 min prior to each round of selection. Pooled inter-β-sheet Cys pair scan libraries were panned in duplicate using phage subjected to heating and centrifugation as described. Wells were washed 15 times with PBS containing 0.05% (v/v) Tween-20 (Sigma) and five times with PBS. Bound phage were eluted with 150 μl of 100 mM triethylamine (Sigma) for 10 min with occasional mixing and neutralized with an equivalent volume of 1 M Tris–HCl, pH 7.5. Phage titers were determined by infection of mid-log phase *E. coli* TG1 cells (*A*_600_ = 0.5) and plating on 2×YT agar plates containing 12.5 μg/ml tetracycline. Phage were amplified by overnight growth of the infected cells in 100 ml 2×YT cultures containing tetracycline (12.5 μg/ml) at 37 °C with 240 rpm shaking and purified from culture supernatants the next day for subsequent panning rounds.

### Expression of V_H_H, V_H_, and V_L_ domains

The coding sequences of sdAbs were synthesized and directionally inserted between the *Eco*RI and *Bam*HI restriction sites of the pSJF2H expression vector by GenScript. Following transformation of *E. coli* TG1 cells, single colonies were grown in 10 ml 2×YT media containing 1% (w/v) glucose and 100 μg/ml ampicillin for 4 h at 37 °C with 250 rpm shaking. The precultures were used to inoculate overnight cultures (37 °C, 250 ml 2×YT medium, 250 rpm shaking) or 5-day cultures (25 °C, 1 l M9 minimal medium, 180 rpm shaking). Expression was induced with 0.5 mM IPTG when the *A*_600_ of 250 ml overnight cultures reached 0.4 to 0.5 and with 100 ml of 10× induction medium/0.1 mM IPTG after 30 h for 1 l M9 cultures (38). Periplasmic proteins were extracted by sucrose shock and His_6_-tagged sdAbs were purified by immobilized metal ion affinity chromatography on a HisTrap HP affinity column (Cytiva Life Sciences) connected to an ÄKTA fast protein liquid chromatography system (Cytiva Life Sciences). Protein yield and purity was assessed by spectrophotometry (absorbance at 280 nm, *A*_280_) and SDS-PAGE.

### Circular dichroism

The night before CD experiments, sdAb proteins (0.1 mg/ml) were dialyzed into 0.1 M sodium phosphate buffer, pH 7.2. CD spectra and temperature-dependent ellipticity measurements were collected using a Jasco J-815 spectropolarimeter (Jasco) equipped with a Peltier thermoelectric–type temperature control system. Samples (0.2 ml) were placed into 1-mm path length cuvettes. Spectral scans over wavelengths from 190 to 250 nm were performed at 25 °C with three accumulations, a scan speed of 200 nm/min, a digital integration time of 1 s, and a bandwidth of 1 nm. Ellipticity was measured at wavelengths of 205 to 210 nm over a temperature range of 25 to 106 °C with intervals of 0.5 °C, a ramp rate of 2 °C/min, a digital integration time of 2 s, and a bandwidth of 1 nm. The data were normalized to a percent scale and fitted to a Boltzmann distribution for calculation of *T*_m_. To determine refolding efficiencies, the scanned samples were cooled to 25 °C and used to obtain second sigmoidal melting curves as described. Refolding efficiencies were calculated as the ratios of the normalized upper plateau ellipticity values for the second melting curves to those of the first melting curves.

### Differential scanning fluorimetry

Differential scanning fluorimetry was performed using SYPRO Orange (Life Technologies) essentially as described previously (18). SYPRO Orange (5 μl) was added to sdAbs (45 μl; 1 mg/ml in PBS) in wells of 96-well thin-wall optical plates. A temperature ramp rate of 1 °C/min was applied using an iQ 5 real-time PCR system (Bio-Rad), and thermal unfolding was monitored by measuring fluorescence (excitation and emission 490 and 575 nm, respectively) at 0.5 °C intervals. *T*_m_s were defined as the temperature at which the maximum rate of change in fluorescent signal [d(relative fluorescence unit)/d*t*] was observed.

### Size-exclusion chromatography–multiangle light scattering

Ultra performance liquid chromatography size-exclusion chromatography–multiangle light scattering (SEC-MALS) was performed using an Acquity BEH-125 column (Waters) connected to an Acquity ultra performance liquid chromatography H-Class Bio system (Waters) with miniDAWN MALS detector and Optilab UT-rEX refractometer (Wyatt Technology). The sdAbs (10–20 μg) were injected at 30 °C in a PBS mobile phase at a flow rate of 0.4 ml/min. Weighted average molecular mass (*M*_MALS_) was calculated using a protein concentration determined from *A*_280_ measurements and extinction coefficients calculated from amino acid sequences. Data were processed using ASTRA 6.1 software (Wyatt Technology; https://www.wyatt.com/products/software/astra.html).

### Surface plasmon resonance

Prior to surface plasmon resonance experiments, all sdAbs were purified by SEC using a Superdex 75 Increase 10/30 GL column (Cytiva Life Sciences) connected to an ÄKTA fast protein liquid chromatography system. The mobile phase for SEC consisted of HBS-EP (10 mM Hepes, pH 7.4, containing 150 mM NaCl, 3 mM ethylenediaminetetraacetic acid and 0.005% (v/v) surfactant P20; Cytiva Life Sciences). EGFR (Cat. No. Z03194-50, GenScript; 263 response units [RUs]), IGF1R (Cat. No. 391-GR-050, R&D Systems; 364 RUs), *C. difficile* toxin A (Cedarlane; 4778 RUs), and *C. difficile* toxin B fragment (aa 1751–2366; a generous gift from Kenneth Ng, University of Windsor; 1246 RUs) were immobilized on CM5 sensor chips by amine coupling in 10 mM sodium acetate, pH 3.5 to 4.5. An ethanolamine-blocked flow cell served as a reference. Six to eight different concentrations of sdAbs (25 pM–7.4 μM, depending on *K*_D_) were injected over the surfaces at 25 °C on a Biacore 3000 instrument (Cytiva Life Sciences) at a flow rate of 20 to 40 μl/min with contact times of 120 to 300 s and dissociation times of 160 to 300 s. The EGFR and toxin A surfaces were regenerated with running buffer, while the IGF1R and toxin B surfaces were regenerated using 10 mM glycine, pH 1.5. Affinities were calculated by fitting the data to a 1:1 binding model or a steady-state binding model using BIAevaluation Software version 4.1.1 (Cytiva Life Sciences; www.cytivalifesciences.com).

For analyses of protein A and protein L binding, protein A (1387 RUs) and protein L (Cat. No. PI21189, Thermo Fisher Scientific; 637 RUs) were immobilized on CM5 sensor chips by amine coupling in 10 mM sodium acetate, pH 4.5. All sdAbs were SEC-purified in HBS-EP (for Biacore 3000 experiments) or HBS-EP+ (for Biacore T200 experiments; identical to HBS-EP but containing 0.05% surfactant P20). An ethanolamine- or ovalbumin-blocked flow cell served as a reference. Various concentrations of V_H_Hs and V_H_s (2.5–10 μM) or V_L_s (500 nM–3 μM) were injected over the surfaces on Biacore 3000 or T200 instruments at a flow rate of 20 μl/min with a contact time of 120 s and a dissociation time of 300 s. The surfaces were regenerated using 10 mM glycine, pH 1.5. The data were fitted to a steady-state binding model using BIAevaluation Software version 3.0 (Biacore T200) or 4.1.1 (Biacore 3000).

### Protease digestion assays

Digestions with pepsin, trypsin, and chymotrypsin were performed essentially as described previously (26). Briefly, titrated working concentrations of enzymes were prepared in 1 mM HCl (pepsin) or PBS containing 10 mM CaCl_2_ (trypsin and chymotrypsin). Five micrograms of each sdAb was digested at 37 °C for 1 h in 20-μl reactions with a final pH of 2 for pepsin digestions. Pepsin digestions were neutralized with 1 M NaOH and trypsin/chymotrypsin digestions were neutralized with a protease inhibitor cocktail (Sigma). The digested sdAbs were separated by SDS-PAGE and band densitometry was conducted using ImageJ v.1.53 (https://imagej.net/ij/). Three independent digestions were conducted for each V_H_H.

### Mass spectrometry

Analyses of free sulfhydryl abundance using LC-MS were performed essentially as previously described (39). Briefly, WT or Cys-engineered sdAbs (60 μg) were buffer exchanged into 100 mM sodium acetate, pH 5.5, containing 6 M GdnHCl, and 20 μg was set aside as the unlabeled control. Another 20 μg was reduced with 5 mM TCEP for 15 min at 37 °C. Both the reduced and unreduced samples were then labeled with EZ-Link maleimide-PEG2-biotin (Thermo Fisher Scientific) for 1 h at room temperature using a 150:1 (mol label:mol protein) ratio for the unreduced samples and a 150:1 (mol label:mol Cys) ratio for the reduced samples. Samples were buffer exchanged again to remove excess label. LC-MS analysis of intact proteins was performed using a Dionex UltiMate 3000 HPLC instrument (Thermo Fisher Scientific) equipped with a POROS R2 (10 μm, 2.1 × 30 mm) column (Thermo Fisher Scientific) coupled to an LTQ-Orbitrap XL mass spectrometer equipped with an electrospray ionization source (Thermo Fisher Scientific). Approximately, 5 μg of protein was injected at a flow rate of 3 ml/min with a column temperature of 80 °C. The mobile phases were 0.1% formic acid in ddH_2_O (A) and acetonitrile (B). Proteins were eluted with a linear gradient of 10% to 75% mobile phase B over 3 min and split at 100 μl/min to the LTQ-Orbitrap XL. MS analysis was conducted in positive electrospray ionization mode using appropriate tune files for analysis of small intact proteins. Data were deconvoluted using the MaxEnt1 algorithm available through MassLynx software (Waters; https://www.waters.com/waters/en_US/MassLynx-MS-Software/nav.htm?cid=513662&locale=en_US).

### Illumina MiSeq sequencing

Sequencing of human expressed V_H_ repertoires was conducted essentially as previously described (40). The study was approved by the Simon Fraser University Research Ethics Board (#2012s0182) and abided by the principles laid out in the Declaration of Helsinki. Peripheral blood mononuclear cells were isolated from the blood of healthy and HIV^+^ individuals by density gradient centrifugation, and resting memory B cells (CD21^+^IgG^+^CD19^+^CD27^+^CD10^−^CD20^+^) were obtained by fluorescence-activated cell sorting as described (41). Total cellular RNA was extracted and reverse transcribed into complementary DNA, and then expressed V_H_ genes were amplified in two rounds of universal tag PCR. The resulting amplicons were pooled and purified by gel extraction, followed by solid-phase reversible immobilization with AMPure XP beads (Beckman-Coulter). The pooled amplicons were quantitated using a Qubit 2.0 fluorometer (Life Technologies) and sequenced using a 500-cycle Reagent Kit V2 and a MiSeq instrument (Illumina) with a 5% PhiX genomic DNA spike. Forward and reverse reads were merged using FLASH (42) with default parameters and quality-filtered using the FASTX toolkit (43) with a stringency of Q30 over ≥95% of each read. The filtered data were processed using IMGT/High-VQUEST (https://www.imgt.org/IMGTindex/IMGTHighV-QUEST.php) (44) and analyzed using custom R scripts, available from the corresponding author by request. Circos analyses were performed using Circos Table Viewer (45).

### Statistical analyses

Differences in proportions of Cys-engineered sdAbs bearing noncanonical disulfide linkages expressing in *E. coli* cells with adequate yields for subsequent experiments compared with WT sdAbs were assessed using Fisher’s exact test. Differences in *T*_m_, α-value, monomer percentage, *K*_D_, and pepsin resistance of Cys-engineered sdAbs bearing noncanonical disulfide linkages compared with WT sdAbs were assessed using the Wilcoxon matched–pairs signed-rank test (two-tailed). *p*-values less than 0.05 were considered statistically significant.

## Data availability

All data associated with this study are available within the article itself and its supporting information file. Raw data are available upon request from the corresponding author (J. T.).

## Supporting information

This article contains supporting information (7).

## Conflict of interest

The authors declare that they have no conflicts of interest with the contents of this article.
