# Supplementary material for: Sequence tolerance of immunoglobulin variable domain framework regions to noncanonical intradomain disulfide linkages
Source: J Biol Chem. 2023 Sep 22;299(11):105278. doi: 10.1016/j.jbc.2023.105278 (PMC10641266; doi:10.1016/j.jbc.2023.105278)
Supplement: Supporting information [file mmc1.docx]

**Supplementary Information**

**Sequence tolerance of immunoglobulin variable domain**

**framework regions to noncanonical intradomain disulfide linkages**

Dae Young Kim^1†^, Hiba Kandalaft^1^, Michael J. Lowden^1^, Qingling Yang^1^, Martin A. Rossotti^1^, Anna Robotham^1^, John F. Kelly^1^, Greg Hussack^1^, Joseph D. Schrag^2^, Kevin A. Henry^1,3^ and Jamshid Tanha^1,3*^

^1^Human Health Therapeutics Research Centre, Life Sciences Division, National Research Council Canada, 100 Sussex Drive, Ottawa, ON, Canada, K1A 0R6

^2^Human Health Therapeutics Research Centre, Life Sciences Division, National Research Council Canada, 6100 Royalmount Avenue, Montréal, QC, Canada, H4P 2R2

^3^Department of Biochemistry, Microbiology and Immunology, Faculty of Medicine, University of Ottawa, 75 Laurier Avenue East, Ottawa, ON, Canada, K1H 8M5

^†^Present address: PnP Biopharm, #1304, 11, Digital-ro 33-gil, Guro-gu, Seoul, Korea, 08380

^*^Corresponding author: Jamshid Tanha, Ph.D.

Email: [jamshid.tanha@nrc-cnrc.gc.ca](mailto:jamshid.tanha@nrc-cnrc.gc.ca)

**Table of Contents**

Supplementary Figures S-3

Supplementary Tables S-10


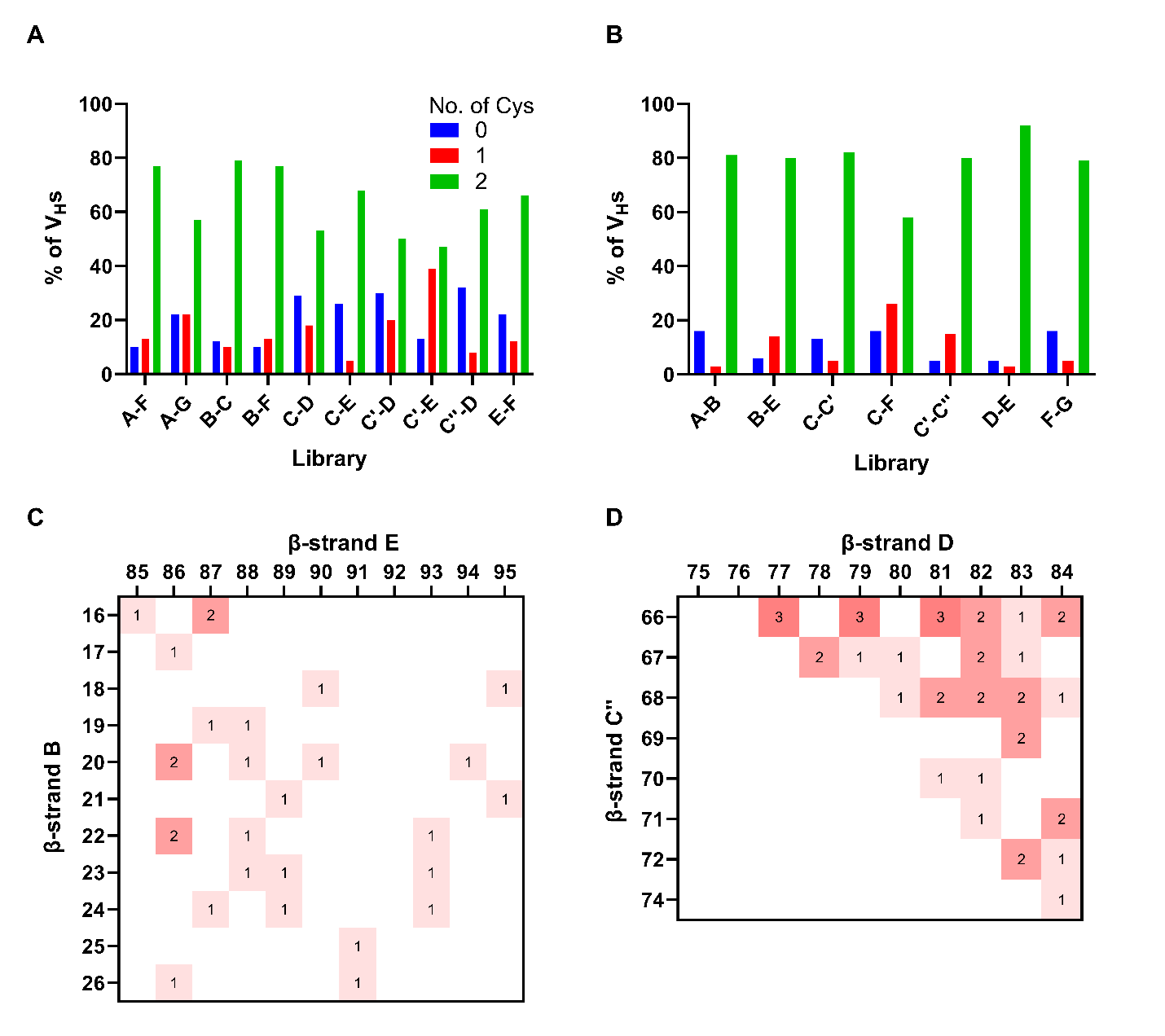


**Figure S1**. **DNA sequencing of the phage-displayed VH413^C23-C104 null^ inter-β-sheet (A) and intra-β-sheet (B) Cys pair scan libraries.** Representative analysis of V_H_ Cys pair diversity is shown for libraries B–E (**C**) and C’’-D (**D**). Each box represents the number of times the indicated Cys pair was observed when a total of 28 and 40 clones were successfully sequenced from each library, respectively, using Sanger sequencing.


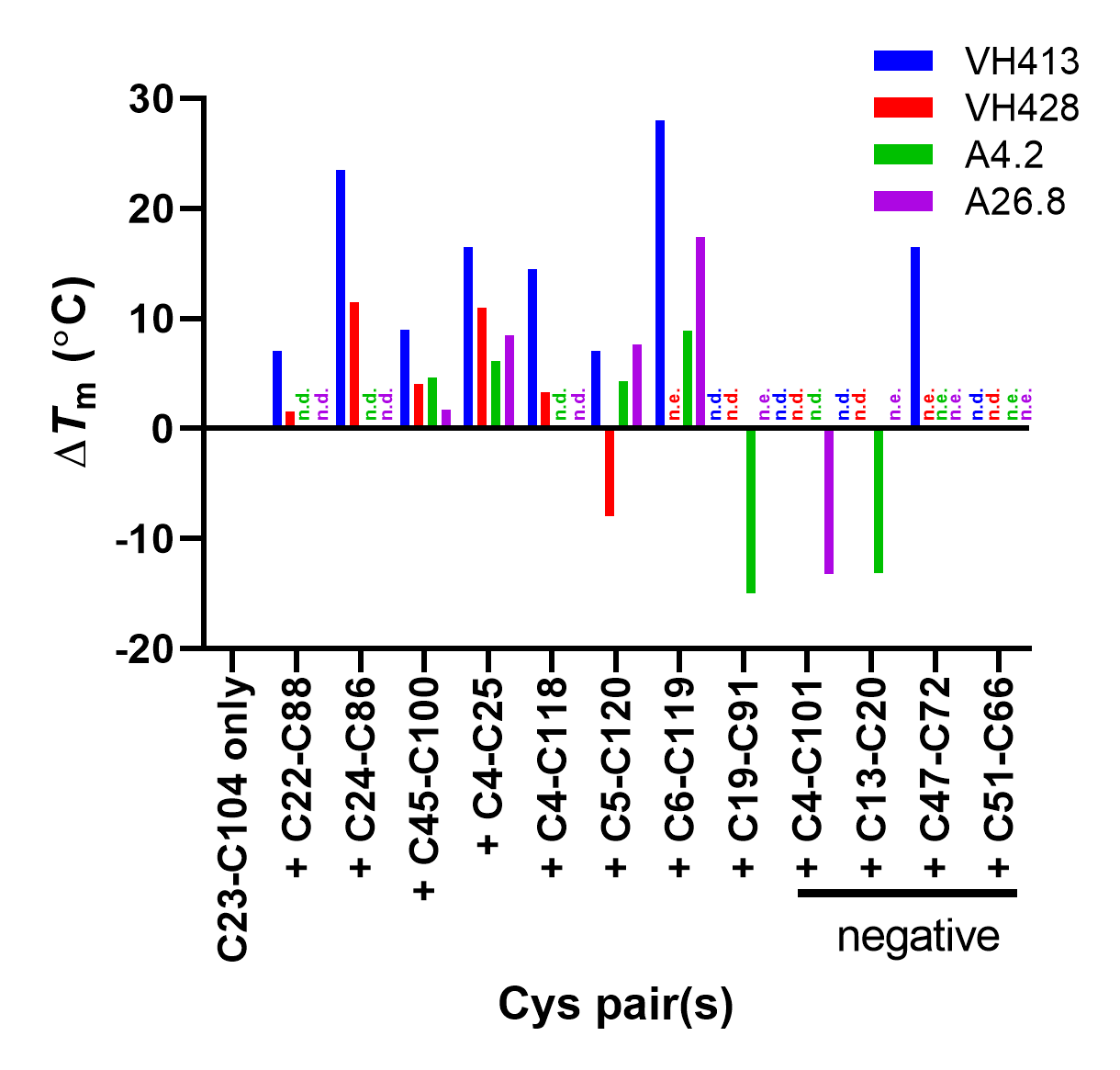


**Figure S2**. **Effect of introducing eight Cys pairs forming putative non-canonical FR disulfide linkages on the *T*_m_s of two V_H_s (VH413 and VH428) and two V_H_Hs (A4.2 and A26.8).** *T*_m_ was measured using differential scanning fluorimetry. The effects of four negative control Cys pairs predicted not to form disulfide linkages on *T_m_* were also assessed. n.d., not determined; n.e., no expression.

**
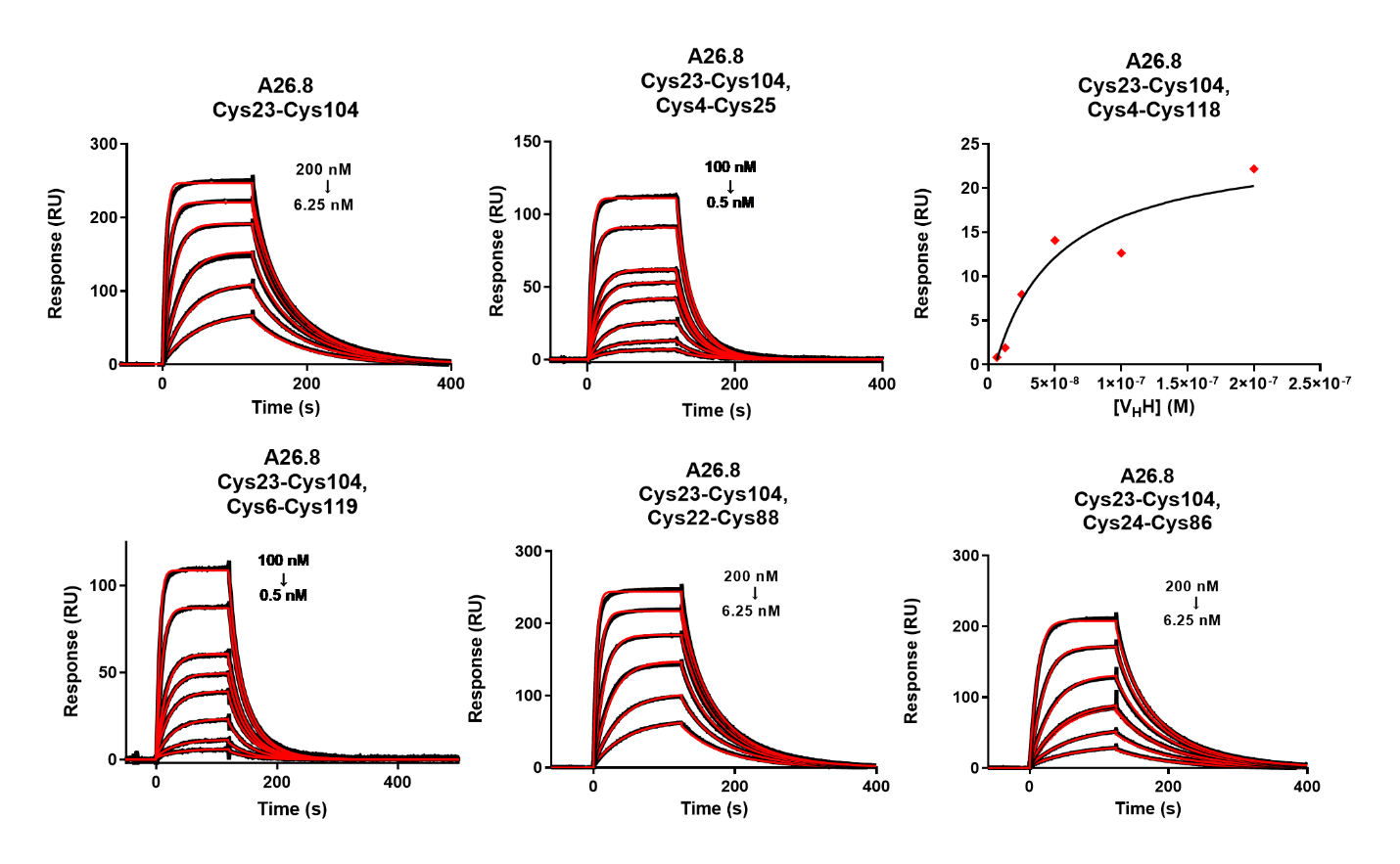
**

**Figure S3**. **Representative SPR sensorgrams for wild-type A26.8 V_H_H and Cys-engineered mutants bearing non-canonical disulfide linkages binding to immobilized *C. difficile* toxin A.** The indicated concentration ranges of V_H_Hs were flowed over the toxin A surface and either multi-cycle kinetic analysis or steady-state analysis (for the Cys4-Cys118 engineered variant) was conducted. Black lines in sensorgrams show data and red lines show fits to a 1:1 binding model.

| **Inter-β-sheet libraries**  **1. Library A-F** | | | | | | | | | | | | | |  |  |  |  |  |  |  |  |  |  |  |  |  |  |  |
| --- | --- | --- | --- | --- | --- | --- | --- | --- | --- | --- | --- | --- | --- | --- | --- | --- | --- | --- | --- | --- | --- | --- | --- | --- | --- | --- | --- | --- |
| A1+F1 | A2+F1 | A3+F1 | | A4+F1 | A5+F1 | A6+F1 | A7+F1 | A8+F1 | A9+F1 | | A10+F1 | A11+F1 | A12+F1 | | | | A13+F1 | | | | | A14+F1 | | | | |  |  |
| A1+F2 | A2+F2 | A3+F2 | | A4+F2 | A5+F2 | A6+F2 | A7+F2 | A8+F2 | A9+F2 | | A10+F2 | A11+F2 | A12+F2 | | | | A13+F2 | | | | | A14+F2 | | | | |  |  |
| A1+F3 | A2+F3 | A3+F3 | | A4+F3 | A5+F3 | A6+F3 | A7+F3 | A8+F3 | A9+F3 | | A10+F3 | A11+F3 | A12+F3 | | | | A13+F3 | | | | | A14+F3 | | | | |  |  |
| A1+F4 | A2+F4 | A3+F4 | | A4+F4 | A5+F4 | A6+F4 | A7+F4 | A8+F4 | A9+F4 | | A10+F4 | A11+F4 | A12+F4 | | | | A13+F4 | | | | | A14+F4 | | | | |  |  |
| A1+F5 | A2+F5 | A3+F5 | | A4+F5 | A5+F5 | A6+F5 | A7+F5 | A8+F5 | A9+F5 | | A10+F5 | A11+F5 | A12+F5 | | | | A13+F5 | | | | | A14+F5 | | | | |  |  |
| A1+F6 | A2+F6 | A3+F6 | | A4+F6 | A5+F6 | A6+F6 | A7+F6 | A8+F6 | A9+F6 | | A10+F6 | A11+F6 | A12+F6 | | | | A13+F6 | | | | | A14+F6 | | | | |  |  |
| A1+F7 | A2+F7 | A3+F7 | | A4+F7 | A5+F7 | A6+F7 | A7+F7 | A8+F7 | A9+F7 | | A10+F7 | A11+F7 | A12+F7 | | | | A13+F7 | | | | | A14+F7 | | | | |  |  |
| A1+F8 | A2+F8 | A3+F8 | | A4+F8 | A5+F8 | A6+F8 | A7+F8 | A8+F8 | A9+F8 | | A10+F8 | A11+F8 | A12+F8 | | | | A13+F8 | | | | | A14+F8 | | | | |  |  |
| **2. Library A-G** | | | | | | | | | | | | | |  |  |  |  |  |  |  |  |  |  |  |  |  |  |  |
| A1+G1 | A1+G2 | A1+G3 | | A1+G4 | A1+G5 | A1+G6 | A1+G7 | A1+G8 | A1+G9 | | A1+G10 | A1+G11 |  | | | | |  | | | | |  | | | | |  |
| A2+G1 | A2+G2 | A2+G3 | | A2+G4 | A2+G5 | A2+G6 | A2+G7 | A2+G8 | A2+G9 | | A2+G10 | A2+G11 |  | | | | |  | | | | |  | | | | |  |
| A3+G1 | A3+G2 | A3+G3 | | A3+G4 | A3+G5 | A3+G6 | A3+G7 | A3+G8 | A3+G9 | | A3+G10 | A3+G11 |  | | | | |  | | | | |  | | | | |  |
| A4+G1 | A4+G2 | A4+G3 | | A4+G4 | A4+G5 | A4+G6 | A4+G7 | A4+G8 | A4+G9 | | A4+G10 | A4+G11 |  | | | | |  | | | | |  | | | | |  |
| A5+G1 | A5+G2 | A5+G3 | | A5+G4 | A5+G5 | A5+G6 | A5+G7 | A5+G8 | A5+G9 | | A5+G10 | A5+G11 |  | | | | |  | | | | |  | | | | |  |
| A6+G1 | A6+G2 | A6+G3 | | A6+G4 | A6+G5 | A6+G6 | A6+G7 | A6+G8 | A6+G9 | | A6+G10 | A6+G11 |  | | | | |  | | | | |  | | | | |  |
| A7+G1 | A7+G2 | A7+G3 | | A7+G4 | A7+G5 | A7+G6 | A7+G7 | A7+G8 | A7+G9 | | A7+G10 | A7+G11 |  | | | | |  | | | | |  | | | | |  |
| A8+G1 | A8+G2 | A8+G3 | | A8+G4 | A8+G5 | A8+G6 | A8+G7 | A8+G8 | A8+G9 | | A8+G10 | A8+G11 |  | | | | |  | | | | |  | | | | |  |
| A9+G1 | A9+G2 | A9+G3 | | A9+G4 | A9+G5 | A9+G6 | A9+G7 | A9+G8 | A9+G9 | | A9+G10 | A9+G11 |  | | | | |  | | | | |  | | | | |  |
| A10+G1 | A10+G2 | A10+G3 | | A10+G4 | A10+G5 | A10+G6 | A10+G7 | A10+G8 | A10+G9 | | A10+G10 | A10+G11 |  | | | | |  | | | | |  | | | | |  |
| A11+G1 | A11+G2 | A11+G3 | | A11+G4 | A11+G5 | A11+G6 | A11+G7 | A11+G8 | A11+G9 | | A11+G10 | A11+G11 |  | | | | |  | | | | |  | | | | |  |
| A12+G1 | A12+G2 | A12+G3 | | A12+G4 | A12+G5 | A12+G6 | A12+G7 | A12+G8 | A12+G9 | | A12+G10 | A12+G11 |  | | | | |  | | | | |  | | | | |  |
| A13+G1 | A13+G2 | A13+G3 | | A13+G4 | A13+G5 | A13+G6 | A13+G7 | A13+G8 | A13+G9 | | A13+G10 | A13+G11 |  | | | | |  | | | | |  | | | | |  |
| A14+G1 | A14+G2 | A14+G3 | | A14+G4 | A14+G5 | A14+G6 | A14+G7 | A14+G8 | A14+G9 | | A14+G10 | A14+G11 |  | | | | |  | | | | |  | | | | |  |
| **3. Library B-C** | | | | | | | | | | | | | |  |  |  |  |  |  |  |  |  |  |  |  |  |  |  |
| B1+C1 | B2+C1 | B3+C1 | | B4+C1 | B5+C1 | B6+C1 | B7+C1 | B8+C1 | B9+C1 | | B10+C1 | B11-C1 |  | | |  | | | |  | | | | |  |  |  |  |
| B1+C2 | B2+C2 | B3+C2 | | B4+C2 | B5+C2 | B6+C2 | B7+C2 | B8+C2 | B9+C2 | | B10+C2 | B11+C2 |  | | |  | | | |  | | | | |  |  |  |  |
| B1+C3 | B2+C3 | B3+C3 | | B4+C3 | B5+C3 | B6+C3 | B7+C3 | B8+C3 | B9+C3 | | B10+C3 | B11+C3 |  | | |  | | | |  | | | | |  |  |  |  |
| B1+C4 | B2+C4 | B3+C4 | | B4+C4 | B5+C4 | B6+C4 | B7+C4 | B8+C4 | B9+C4 | | B10+C4 | B11+C4 |  | | |  | | | |  | | | | |  |  |  |  |
| B1+C5 | B2+C5 | B3+C5 | | B4+C5 | B5+C5 | B6+C5 | B7+C5 | B8+C5 | B9+C5 | | B10+C5 | B11+C5 |  | | |  | | | |  | | | | |  |  |  |  |
| B1+C6 | B2+C6 | B3+C6 | | B4+C6 | B5+C6 | B6+C6 | B7+C6 | B8+C6 | B9+C6 | | B10+C6 | B11+C6 |  | | |  | | | |  | | | | |  |  |  |  |
| B1+C7 | B2+C7 | B3+C7 | | B4+C7 | B5+C7 | B6+C7 | B7+C7 | B8+C7 | B9+C7 | | B10+C7 | B11+C7 |  | | |  | | | |  | | | | |  |  |  |  |
| B1+C8 | B2+C8 | B3+C8 | | B4+C8 | B5+C8 | B6+C8 | B7+C8 | B8+C8 | B9+C8 | | B10+C8 | B11+C8 |  | | |  | | | |  | | | | |  |  |  |  |
| **4. Library B-F** | | | | | | | | | | | | | |  |  |  |  |  |  |  |  |  |  |  |  |  |  |  |
| B1+F1 | B2+F1 | B3+F1 | | B4+F1 | B5+F1 | B6+F1 | B7+F1 | B8+F1 | B9+F1 | | B10+F1 | B11+F1 |  | | |  | | | |  | | | | |  |  |  |  |
| B1+F2 | B2+F2 | B3+F2 | | B4+F2 | B5+F2 | B6+F2 | B7+F2 | B8+F2 | B9+F2 | | B10+F2 | B11+F2 |  | | |  | | | |  | | | | |  |  |  |  |
| B1+F3 | B2+F3 | B3+F3 | | B4+F3 | B5+F3 | B6+F3 | B7+F3 | B8+F3 | B9+F3 | | B10+F3 | B11+F3 |  | | |  | | | |  | | | | |  |  |  |  |
| B1+F4 | B2+F4 | B3+F4 | | B4+F4 | B5+F4 | B6+F4 | B7+F4 | B8+F4 | B9+F4 | | B10+F4 | B11+F4 |  | | |  | | | |  | | | | |  |  |  |  |
| B1+F5 | B2+F5 | B3+F5 | | B4+F5 | B5+F5 | B6+F5 | B7+F5 | B8+F5 | B9+F5 | | B10+F5 | B11+F5 |  | | |  | | | |  | | | | |  |  |  |  |
| B1+F6 | B2+F6 | B3+F6 | | B4+F6 | B5+F6 | B6+F6 | B7+F6 | B8+F6 | B9+F6 | | B10+F6 | B11+F6 |  | | |  | | | |  | | | | |  |  |  |  |
| B1+F7 | B2+F7 | B3+F7 | | B4+F7 | B5+F7 | B6+F7 | B7+F7 | B8+F7 | B9+F7 | | B10+F7 | B11+F7 |  | | |  | | | |  | | | | |  |  |  |  |
| B1+F8 | B2+F8 | B3+F8 | | B4+F8 | B5+F8 | B6+F8 | B7+F8 | B8+F8 | B9+F8 | | B10+F8 | B11+F8 |  | | |  | | | |  | | | | |  |  |  |  |
| **5. Library C-D** | | | | | | | | | | | | | |  |  |  |  |  |  |  |  |  |  |  |  |  |  |  |
| C1+D1 | C1+D2 | C1+D3 | | C1+D4 | C1+D5 | C1+D6 | C1+D7 | C1+D8 | C1+D9 | | C1+D10 |  | | |  | | | |  | | | | |  | | | | |
| C2+D1 | C2+D2 | C2+D3 | | C2+D4 | C2+D5 | C2+D6 | C2+D7 | C2+D8 | C2+D9 | | C2+D10 |  | | |  | | | |  | | | | |  | | | | |
| C3+D1 | C3+D2 | C3+D3 | | C3+D4 | C3+D5 | C3+D6 | C3+D7 | C3+D8 | C3+D9 | | C3+D10 |  | | |  | | | |  | | | | |  | | | | |
| C4+D1 | C4+D2 | C4+D3 | | C4+D4 | C4+D5 | C4+D6 | C4+D7 | C4+D8 | C4+D9 | | C4+D10 |  | | |  | | | |  | | | | |  | | | | |
| C5+D1 | C5+D2 | C5+D3 | | C5+D4 | C5+D5 | C5+D6 | C5+D7 | C5+D8 | C5+D9 | | C5+D10 |  | | |  | | | |  | | | | |  | | | | |
| C6+D1 | C6+D2 | C6+D3 | | C6+D4 | C6+D5 | C6+D6 | C6+D7 | C6+D8 | C6+D9 | | C6+D10 |  | | |  | | | |  | | | | |  | | | | |
| C7+D1 | C7+D2 | C7+D3 | | C7+D4 | C7+D5 | C7+D6 | C7+D7 | C7+D8 | C7+D9 | | C7+D10 |  | | |  | | | |  | | | | |  | | | | |
| C8+D1 | C8+D2 | C8+D3 | | C8+D4 | C8+D5 | C8+D6 | C8+D7 | C8+D8 | C8+D9 | | C8+D10 |  | | |  | | | |  | | | | |  | | | | |
| **6. Library C-E** | | | | | | | | | |  |  |  |  |  |  |  |  |  |  |  |  |  |  |  |  |  |  |  |
| C1+E1 | C1+E2 | C1+E3 | | C1+E4 | C1+E5 | C1+E6 | C1+E7 | C1+E8 | C1+E9 | | C1+E10 | C1+E11 | C1+E12 | | | |  |  |  |  |  |  |  |  |  |  |  |  |
| C2+E1 | C2+E2 | C2+E3 | | C2+E4 | C2+E5 | C2+E6 | C2+E7 | C2+E8 | C2+E9 | | C2+E10 | C2+E11 | C2+E12 | | | |  |  |  |  |  |  |  |  |  |  |  |  |
| C3+E1 | C3+E2 | C3+E3 | | C3+E4 | C3+E5 | C3+E6 | C3+E7 | C3+E8 | C3+E9 | | C3+E10 | C3+E11 | C3+E12 | | | |  |  |  |  |  |  |  |  |  |  |  |  |
| C4+E1 | C4+E2 | C4+E3 | | C4+E4 | C4+E5 | C4+E6 | C4+E7 | C4+E8 | C4+E9 | | C4+E10 | C4+E11 | C4+E12 | | | |  |  |  |  |  |  |  |  |  |  |  |  |
| C5+E1 | C5+E2 | C5+E3 | | C5+E4 | C5+E5 | C5+E6 | C5+E7 | C5+E8 | C5+E9 | | C5+E10 | C5+E11 | C5+E12 | | | |  |  |  |  |  |  |  |  |  |  |  |  |
| C6+E1 | C6+E2 | C6+E3 | | C6+E4 | C6+E5 | C6+E6 | C6+E7 | C6+E8 | C6+E9 | | C6+E10 | C6+E11 | C6+E12 | | | |  |  |  |  |  |  |  |  |  |  |  |  |
| C7+E1 | C7+E2 | C7+E3 | | C7+E4 | C7+E5 | C7+E6 | C7+E7 | C7+E8 | C7+E9 | | C7+E10 | C7+E11 | C7+E12 | | | |  |  |  |  |  |  |  |  |  |  |  |  |
| C8+E1 | C8+E2 | C8+E3 | | C8+E4 | C8+E5 | C8+E6 | C8+E7 | C8+E8 | C8+E9 | | C8+E10 | C8+E11 | C8+E12 | | | |  |  |  |  |  |  |  |  |  |  |  |  |
| **7. Library C'-D** | | | | | | | | | | | | | |  |  |  |  |  |  |  |  |  |  |  |  |  |  |  |
| C’1+D1 | C’1+D2 | C’1+D3 | | C’1+D4 | C’1+D5 | C’1+D6 | C’1+D7 | C’1+D8 | C’1+D9 | | C’1+D10 |  | | |  | | | |  | | | | |  | | | | |
| C’2+D1 | C’2+D2 | C’2+D3 | | C’2+D4 | C’2+D5 | C’2+D6 | C’2+D7 | C’2+D8 | C’2+D9 | | C’2+D10 |  | | |  | | | |  | | | | |  | | | | |
| C’3+D1 | C’3+D2 | C’3+D3 | | C’3+D4 | C’3+D5 | C’3+D6 | C’3+D7 | C’3+D8 | C’3+D9 | | C’3+D10 |  | | |  | | | |  | | | | |  | | | | |
| C’4+D1 | C’4+D2 | C’4+D3 | | C’4+D4 | C’4+D5 | C’4+D6 | C’4+D7 | C’4+D8 | C’4+D9 | | C’4+D10 |  | | |  | | | |  | | | | |  | | | | |
| C’5+D1 | C’5+D2 | C’5+D3 | | C’5+D4 | C’5+D5 | C’5+D6 | C’5+D7 | C’5+D8 | C’5+D9 | | C’5+D10 |  | | |  | | | |  | | | | |  | | | | |
| C’6+D1 | C’6+D2 | C’6+D3 | | C’6+D4 | C’6+D5 | C’6+D6 | C’6+D7 | C’6+D8 | C’6+D9 | | C’6+D10 |  | | |  | | | |  | | | | |  | | | | |
| C’7+D1 | C’7+D2 | C’7+D3 | | C’7+D4 | C’7+D5 | C’7+D6 | C’7+D7 | C’7+D8 | C’7+D9 | | C’7+D10 |  | | |  | | | |  | | | | |  | | | | |
| C’8+D1 | C’8+D2 | C’8+D3 | | C’8+D4 | C’8+D5 | C’8+D6 | C’8+D7 | C’8+D8 | C’8+D9 | | C’8+D10 |  | | |  | | | |  | | | | |  | | | | |
| C’9+D1 | C’9+D2 | C’9+D3 | | C’9+D4 | C’9+D5 | C’9+D6 | C’9+D7 | C’9+D8 | C’9+D9 | | C’9+D10 |  | | |  | | | |  | | | | |  |  |  |  |  |
| **8. Library C'-E** | | | | | | | | | |  |  |  |  |  |  |  |  |  |  |  |  |  |  |  |  |  |  |  |
| C’1+E1 | C’1+E2 | C’1+E3 | | C’1+E4 | C’1+E5 | C’1+E6 | C’1+E7 | C’1+E8 | C’1+E9 | | C’1+E10 | C’1+E11 | C’1+E12 | | | |  |  |  |  |  |  |  |  |  |  |  |  |
| C’2+E1 | C’2+E2 | C’2+E3 | | C’2+E4 | C’2+E5 | C’2+E6 | C’2+E7 | C’2+E8 | C’2+E9 | | C’2+E10 | C’2+E11 | C’2+E12 | | | |  |  |  |  |  |  |  |  |  |  |  |  |
| C’3+E1 | C’3+E2 | C’3+E3 | | C’3+E4 | C’3+E5 | C’3+E6 | C’3+E7 | C’3+E8 | C’3+E9 | | C’3+E10 | C’3+E11 | C’3+E12 | | | |  |  |  |  |  |  |  |  |  |  |  |  |
| C’4+E1 | C’4+E2 | C’4+E3 | | C’4+E4 | C’4+E5 | C’4+E6 | C’4+E7 | C’4+E8 | C’4+E9 | | C’4+E10 | C’4+E11 | C’4+E12 | | | |  |  |  |  |  |  |  |  |  |  |  |  |
| C’5+E1 | C’5+E2 | C’5+E3 | | C’5+E4 | C’5+E5 | C’5+E6 | C’5+E7 | C’5+E8 | C’5+E9 | | C’5+E10 | C’5+E11 | C’5+E12 | | | |  |  |  |  |  |  |  |  |  |  |  |  |
| C’6+E1 | C’6+E2 | C’6+E3 | | C’6+E4 | C’6+E5 | C’6+E6 | C’6+E7 | C’6+E8 | C’6+E9 | | C’6+E10 | C’6+E11 | C’6+E12 | | | |  |  |  |  |  |  |  |  |  |  |  |  |
| C’7+E1 | C’7+E2 | C’7+E3 | | C’7+E4 | C’7+E5 | C’7+E6 | C’7+E7 | C’7+E8 | C’7+E9 | | C’7+E10 | C’7+E11 | C’7+E12 | | | |  |  |  |  |  |  |  |  |  |  |  |  |
| C’8+E1 | C’8+E2 | C’8+E3 | | C’8+E4 | C’8+E5 | C’8+E6 | C’8+E7 | C’8+E8 | C’8+E9 | | C’8+E10 | C’8+E11 | C’8+E12 | | | |  |  |  |  |  |  |  |  |  |  |  |  |
| C’9+E1 | C’9+E2 | C’9+E3 | | C’9+E4 | C’9+E5 | C’9+E6 | C’9+E7 | C’9+E8 | C’9+E9 | | C’9+E10 | C’9+E11 | C’9+E12 | | | |  |  |  |  |  |  |  |  |  |  |  |  |
| **9. Library C”-D** | | |  |  |  |  |  |  |  |  |  |  |  |  |  |  |  |  |  |  |  |  |  |  |  |  |  |  |
| C”1-D1 | C”1-D2 | C”1+D3 | | C”1+D4 | C”1+D5 | C”1+D6 | C”1+D7 | C”1+D8 | C”1+D9 | | C”1+D10 |  | | |  | | | |  | | | | |  | | | | |
| C”2-D1 | C”2-D2 | C”2-D3 | | C”2+D4 | C”2+D5 | C”2+D6 | C”2+D7 | C”2+D8 | C”2+D9 | | C”2+D10 |  | | |  | | | |  | | | | |  | | | | |
| C”3-D1 | C”3-D2 | C”3-D3 | | C”3-D4 | C”3+D5 | C”3+D6 | C”3+D7 | C”3+D8 | C”3+D9 | | C”3+D10 |  | | |  | | | |  | | | | |  | | | | |
| C”4-D1 | C”4-D2 | C”4-D3 | | C”4-D4 | C”4-D5 | C”4+D6 | C”4+D7 | C”4+D8 | C”4+D9 | | C”4+D10 |  | | |  | | | |  | | | | |  | | | | |
| C”5-D1 | C”5-D2 | C”5-D3 | | C”5-D4 | C”5-D5 | C”5-D6 | C”5+D7 | C”5+D8 | C”5+D9 | | C”5+D10 |  | | |  | | | |  | | | | |  | | | | |
| C”6-D1 | C”6-D2 | C”6-D3 | | C”6-D4 | C”6-D5 | C”6-D6 | C”6-D7 | C”6+D8 | C”6+D9 | | C”6+D10 |  | | |  | | | |  | | | | |  | | | | |
| C”7-D1 | C”7-D2 | C”7-D3 | | C”7-D4 | C“7-D5 | C”7-D6 | C”7-D7 | C”7-D8 | C”7+D9 | | C”7+D10 |  | | |  | | | |  | | | | |  | | | | |
| C”8-D1 | C”8-D2 | C”8-D3 | | C”8-D4 | C”8-D5 | C”8-D6 | C”8-D7 | C”8-D8 | C”8-D9 | | C”8+D10 |  | | |  | | | |  | | | | |  | | | | |
| **10. Library E-F** | | | | | | | | | | | | | |  |  |  |  |  |  |  |  |  |  |  |  |  |  |  |
| E1+F1 | E2+F1 | E3+F1 | | E4-F1 | E5-F1 | E6-F1 | E7-F1 | E8-F1 | E9-F1 | | E10-F1 | E11-F1 | E12-F1 | | | |  | | | |  | | | | |  |  |  |
| E1+F2 | E2+F2 | E3+F2 | | E4+F2 | E5-F2 | E6-F2 | E7-F2 | E8-F2 | E9-F2 | | E10-F2 | E11-F2 | E12-F2 | | | |  | | | |  | | | | |  |  |  |
| E1+F3 | E2+F3 | E3+F3 | | E4+F3 | E5+F3 | E6-F3 | E7-F3 | E8-F3 | E9-F3 | | E10-F3 | E11-F3 | E12-F3 | | | |  | | | |  | | | | |  |  |  |
| E1+F4 | E2+F4 | E3+F4 | | E4+F4 | E5+F4 | E6+F4 | E7-F4 | E8-F4 | E9-F4 | | E10-F4 | E11-F4 | E12-F4 | | | |  | | | |  | | | | |  |  |  |
| E1+F5 | E2+F5 | E3+F5 | | E4+F5 | E5+F5 | E6+F5 | E7+F5 | E8-F5 | E9-F5 | | E10-F5 | E11-F5 | E12-F5 | | | |  | | | |  | | | | |  |  |  |
| E1+F6 | E2+F6 | E3+F6 | | E4+F6 | E5+F6 | E6+F6 | E7+F6 | E8+F6 | E9-F6 | | E10-F6 | E11-F6 | E12-F6 | | | |  | | | |  | | | | |  |  |  |
| E1+F7 | E2+F7 | E3+F7 | | E4+F7 | E5+F7 | E6+F7 | E7+F7 | E8+F7 | E9+F7 | | E10-F7 | E11-F7 | E12-F7 | | | |  | | | |  | | | | |  |  |  |
| E1+F8 | E2+F8 | E3+F8 | | E4+F8 | E5+F8 | E6+F8 | E7+F8 | E8+F8 | E9+F8 | | E10+F8 | E11-F8 | E12-F8 | | | |  | | | |  | | | | |  |  |  |

**Intra-β-sheet libraries**

**11. Library A-B**

| A1+B1 | A1+B2 | A1+B3 | A1+B4 | A1+B5 | A1+B6 | A1+B7 | A1+B8 | A1+B9 | A1+B10 | A1+B11 |
| --- | --- | --- | --- | --- | --- | --- | --- | --- | --- | --- |
| A2+B1 | A2+B2 | A2+B3 | A2+B4 | A2+B5 | A2+B6 | A2+B7 | A2+B8 | A2+B9 | A2+B10 | A2+B11 |
| A3+B1 | A3+B2 | A3+B3 | A3+B4 | A3+B5 | A3+B6 | A3+B7 | A3+B8 | A3+B9 | A3+B10 | A3+B11 |
| A4+B1 | A4+B2 | A4+B3 | A4+B4 | A4+B5 | A4+B6 | A4+B7 | A4+B8 | A4+B9 | A4+B10 | A4+B11 |
| A5+B1 | A5+B2 | A5+B3 | A5+B4 | A5+B5 | A5+B6 | A5+B7 | A5+B8 | A5+B9 | A5+B10 | A5+B11 |
| A6-B1 | A6+B2 | A6+B3 | A6+B4 | A6+B5 | A6+B6 | A6+B7 | A6+B8 | A6+B9 | A6+B10 | A6+B11 |
| A7-B1 | A7-B2 | A7+B3 | A7+B4 | A7+B5 | A7+B6 | A7+B7 | A7+B8 | A7+B9 | A7+B10 | A7+B11 |
| A8-B1 | A8-B2 | A8-B3 | A8+B4 | A8+B5 | A8+B6 | A8+B7 | A8+B8 | A8+B9 | A8+B10 | A8+B11 |
| A9-B1 | A9-B2 | A9-B3 | A9-B4 | A9+B5 | A9+B6 | A9+B7 | A9+B8 | A9+B9 | A9+B10 | A9+B11 |
| A10-B1 | A10-B2 | A10-B3 | A10-B4 | A10-B5 | A10+B6 | A10+B7 | A10+B8 | A10+B9 | A10+B10 | A10+B11 |
| A11-B1 | A11-B2 | A11-B3 | A11-B4 | A11-B5 | A11-B6 | A11+B7 | A11+B8 | A11+B9 | A11+B10 | A11+B11 |
| A12-B1 | A12-B2 | A12-B3 | A12-B4 | A12-B5 | A12-B6 | A12-B7 | A12+B8 | A12+B9 | A12+B10 | A12+B11 |
| A13-B1 | A13-B2 | A13-B3 | A13-B4 | A13-B5 | A13-B6 | A13-B7 | A13-B8 | A13+B9 | A13+B10 | A13+B11 |
| A14-B1 | A14-B2 | A14-B3 | A14-B4 | A14-B5 | A14-B6 | A14-B7 | A14-B8 | A14-B9 | A14+B10 | A14+B11 |

**12. Library B-E**

| B1+E1 | B2+E1 | B3+E1 | B4+E1 | B5+E1 | B6+E1 | B7+E1 | B8+E1 | B9+E1 | B10+E1 | B11+E1 |
| --- | --- | --- | --- | --- | --- | --- | --- | --- | --- | --- |
| B1+E2 | B2+E2 | B3+E2 | B4+E2 | B5+E2 | B6+E2 | B7+E2 | B8+E2 | B9+E2 | B10+E2 | B11+E2 |
| B1+E3 | B2+E3 | B3+E3 | B4+E3 | B5+E3 | B6+E3 | B7+E3 | B8+E3 | B9+E3 | B10+E3 | B11+E3 |
| B1+E4 | B2+E4 | B3+E4 | B4+E4 | B5+E4 | B6+E4 | B7+E4 | B8+E4 | B9+E4 | B10+E4 | B11+E4 |
| B1+E5 | B2+E5 | B3+E5 | B4+E5 | B5+E5 | B6+E5 | B7+E5 | B8+E5 | B9+E5 | B10+E5 | B11+E5 |
| B1+E6 | B2+E6 | B3+E6 | B4+E6 | B5+E6 | B6+E6 | B7+E6 | B8+E6 | B9+E6 | B10+E6 | B11+E6 |
| B1+E7 | B2+E7 | B3+E7 | B4+E7 | B5+E7 | B6+E7 | B7+E7 | B8+E7 | B9+E7 | B10+E7 | B11+E7 |
| B1+E8 | B2+E8 | B3+E8 | B4+E8 | B5+E8 | B6+E8 | B7+E8 | B8+E8 | B9+E8 | B10+E8 | B11+E8 |
| B1+E9 | B2+E9 | B3+E9 | B4+E9 | B5+E9 | B6+E9 | B7+E9 | B8+E9 | B9+E9 | B10+E9 | B11+E9 |
| B1+E10 | B2+E10 | B3+E10 | B4+E10 | B5+E10 | B6+E10 | B7+E10 | B8+E10 | B9+E10 | B10+E10 | B11+E10 |
| B1+E11 | B2+E11 | B3+E11 | B4+E11 | B5+E11 | B6+E11 | B7+E11 | B8+E11 | B9+E11 | B10+E11 | B11+E11 |
| B1+E12 | B2+E12 | B3+E12 | B4+E12 | B5+E12 | B6+E12 | B7+E12 | B8+E12 | B9+E12 | B10+E12 | B11+E12 |

**13. Library C-C’**

| C1-C’1 | C2-C’1 | C3-C’1 | C4-C’1 | C5-C’1 | C6-C’1 | C7-C’1 | C8-C’1 |
| --- | --- | --- | --- | --- | --- | --- | --- |
| C1-C’2 | C2-C’2 | C3-C’2 | C4-C’2 | C5-C’2 | C6-C’2 | C7-C’2 | C8-C’2 |
| C1-C’3 | C2-C’3 | C3-C’3 | C4-C’3 | C5-C’3 | C6-C’3 | C7-C’3 | C8-C’3 |
| C1+C’4 | C2-C’4 | C3-C’4 | C4-C’4 | C5-C’4 | C6-C’4 | C7-C’4 | C8-C’4 |
| C1+C’5 | C2+C’5 | C3-C’5 | C4-C’5 | C5-C’5 | C6-C’5 | C7-C’5 | C8-C’5 |
| C1+C’6 | C2+C’6 | C3+C’6 | C4-C’6 | C5-C’6 | C6-C’6 | C7-C’6 | C8-C’6 |
| C1+C’7 | C2+C’7 | C3+C’7 | C4+C’7 | C5-C’7 | C6-C’7 | C7-C’7 | C8-C’7 |
| C1+C’8 | C2+C’8 | C3+C’8 | C4+C’8 | C5+C’8 | C6-C’8 | C7-C’8 | C8-C’8 |
| C1+C’9 | C2+C’9 | C3+C’9 | C4+C’9 | C5+C’9 | C6+C’9 | C7-C’9 | C8-C’9 |

**14. Library C-F**

| C1+F1 | C2+F1 | C3+F1 | C4+F1 | C5+F1 | C6+F1 | C7+F1 | C8+F1 |
| --- | --- | --- | --- | --- | --- | --- | --- |
| C1+F2 | C2+F2 | C3+F2 | C4+F2 | C5+F2 | C6+F2 | C7+F2 | C8+F2 |
| C1+F3 | C2+F3 | C3+F3 | C4+F3 | C5+F3 | C6+F3 | C7+F3 | C8+F3 |
| C1+F4 | C2+F4 | C3+F4 | C4+F4 | C5+F4 | C6+F4 | C7+F4 | C8+F4 |
| C1+F5 | C2+F5 | C3+F5 | C4+F5 | C5+F5 | C6+F5 | C7+F5 | C8+F5 |
| C1+F6 | C2+F6 | C3+F6 | C4+F6 | C5+F6 | C6+F6 | C7+F6 | C8+F6 |
| C1+F7 | C2+F7 | C3+F7 | C4+F7 | C5+F7 | C6+F7 | C7+F7 | C8+F7 |
| C1+F8 | C2+F8 | C3+F8 | C4+F8 | C5+F8 | C6+F8 | C7+F8 | C8+F8 |

**15. Library C’-C”**

| C’1+C”1 | C’2+C”1 | C’3+C”1 | C’4+C”1 | C’5+C”1 | C’6+C”1 | C’7+C”1 | C’8-C”1 | C’9-C”1 |
| --- | --- | --- | --- | --- | --- | --- | --- | --- |
| C’1+C”2 | C’2+C”2 | C’3+C”2 | C’4+C”2 | C’5+C”2 | C’6+C”2 | C’7+C”2 | C’8+C”2 | C’9-C”2 |
| C’1+C”3 | C’2+C”3 | C’3+C”3 | C’4+C”3 | C’5+C”3 | C’6+C”3 | C’7+C”3 | C’8+C”3 | C’9+C”3 |
| C’1+C”4 | C’2+C”4 | C’3+C”4 | C’4+C”4 | C’5+C”4 | C’6+C”4 | C’7+C”4 | C’8+C”4 | C’9+C”4 |
| C’1+C”5 | C’2+C”5 | C’3+C”5 | C’4+C”5 | C’5+C”5 | C’6+C”5 | C’7+C”5 | C’8+C”5 | C’9+C”5 |
| C’1+C”6 | C’2+C”6 | C’3+C”6 | C’4+C”6 | C’5+C”6 | C’6+C”6 | C’7+C”6 | C’8+C”6 | C’9+C”6 |
| C’1+C”7 | C’2+C”7 | C’3+C”7 | C’4+C”7 | C’5+C”7 | C’6+C”7 | C’7+C”7 | C’8+C”7 | C’9+C”7 |
| C’1+C”8 | C’2+C”8 | C’3+C”8 | C’4+C”8 | C’5+C”8 | C’6+C”8 | C’7+C”8 | C’8+C”8 | C’9+C”8 |

**16. Library D-E**

| D1+E1 | D2-E1 | D3-E1 | D4-E1 | D5-E1 | D6-E1 | D7-E1 | D8-E1 | D9-E1 | D10-E1 |
| --- | --- | --- | --- | --- | --- | --- | --- | --- | --- |
| D1+E2 | D2+E2 | D3-E2 | D4-E2 | D5-E2 | D6-E2 | D7-E2 | D8-E2 | D9-E2 | D10-E2 |
| D1+E3 | D2+E3 | D3+E3 | D4-E3 | D5-E3 | D6-E3 | D7-E3 | D8-E3 | D9-E3 | D10-E3 |
| D1+E4 | D2+E4 | D3+E4 | D4+E4 | D5-E4 | D6-E4 | D7-E4 | D8-E4 | D9-E4 | D10-E4 |
| D1+E5 | D2+E5 | D3+E5 | D4+E5 | D5+E5 | D6-E5 | D7-E5 | D8-E5 | D9-E5 | D10-E5 |
| D1+E6 | D2+E6 | D3+E6 | D4+E6 | D5+E6 | D6+E6 | D7-E6 | D8-E6 | D9-E6 | D10-E6 |
| D1+E7 | D2+E7 | D3+E7 | D4+E7 | D5+E7 | D6+E7 | D7+E7 | D8-E7 | D9-E7 | D10-E7 |
| D1+E8 | D2+E8 | D3+E8 | D4+E8 | D5+E8 | D6+E8 | D7+E8 | D8+E8 | D9-E8 | D10-E8 |
| D1+E9 | D2+E9 | D3+E9 | D4+E9 | D5+E9 | D6+E9 | D7+E9 | D8+E9 | D9+E9 | D10-E9 |
| D1+E10 | D2+E10 | D3+E10 | D4+E10 | D5+E10 | D6+E10 | D7+E10 | D8+E10 | D9+E10 | D10+E10 |
| D1+E11 | D2+E11 | D3+E11 | D4+E11 | D5+E11 | D6+E11 | D7+E11 | D8+E11 | D9+E11 | D10+E11 |
| D1+E12 | D2+E12 | D3+E12 | D4+E12 | D5+E12 | D6+E12 | D7+E12 | D8+E12 | D9+E12 | D10+E12 |

**17. Library F-G**

| F1+G1 | F1+G2 | F1+G3 | F1+G4 | F1+G5 | F1+G6 | F1+G7 | F1+G8 | F1+G9 | F1+G10 | F1+G11 |
| --- | --- | --- | --- | --- | --- | --- | --- | --- | --- | --- |
| F2+G1 | F2+G2 | F2+G3 | F2+G4 | F2+G5 | F2+G6 | F2+G7 | F2+G8 | F2+G9 | F2+G10 | F2+G11 |
| F3+G1 | F3+G2 | F3+G3 | F3+G4 | F3+G5 | F3+G6 | F3+G7 | F3+G8 | F3+G9 | F3+G10 | F3+G11 |
| F4+G1 | F4+G2 | F4+G3 | F4+G4 | F4+G5 | F4+G6 | F4+G7 | F4+G8 | F4+G9 | F4+G10 | F4+G11 |
| F5+G1 | F5+G2 | F5+G3 | F5+G4 | F5+G5 | F5+G6 | F5+G7 | F5+G8 | F5+G9 | F5+G10 | F5+G11 |
| F6+G1 | F6+G2 | F6+G3 | F6+G4 | F6+G5 | F6+G6 | F6+G7 | F6+G8 | F6+G9 | F6+G10 | F6+G11 |
| F7+G1 | F7+G2 | F7+G3 | F7+G4 | F7+G5 | F7+G6 | F7+G7 | F7+G8 | F7+G9 | F7+G10 | F7+G11 |
| F8+G1 | F8+G2 | F8+G3 | F8+G4 | F8+G5 | F8+G6 | F8+G7 | F8+G8 | F8+G9 | F8+G10 | F8+G11 |

**Figure S4**. **Annealing of mutagenic oligonucleotides in 96-well plates for construction of the 17 VH413^C23-C104 null^ Cys pair scan libraries.** Sequences of mutagenic oligonucleotides are listed in Table S9. Note that “+” refers to a mutagenesis reaction using a combination of two oligonucleotides while “-” refers to a mutagenesis reaction using a single oligonucleotide spanning two contiguous β-strands in the primary amino acid sequence (also shown in red font). In each reaction, two amino acid positions in two different β-strands are targeted for substitutions with Cys.

**Table S1**. **Cys pairs identified by panning the six intra-β-sheet VH413^C23-C104 null^ Cys pair scan libraries for four rounds with or without heat treatment.**

| **Cys pair** | **Library** | **Round 2 (%)** | | **Round 3 (%)** | | **Round 4 (%)** | |
| --- | --- | --- | --- | --- | --- | --- | --- |
|  |  | **No heat** | **Heat (50°C)** | **No heat** | **Heat (50°C)** | **No heat** | **Heat (50°C)** |
| **4-25** | A–B | 2.5 | - | - | - | - | - |
| 13-20^a^ | A–B | - | 2.6 | - | - | - | - |
| 16-87^a^ | B–E | - | 2.6 | - | - | - | - |
| **19-91** | B–E | - | 2.6 | - | - | - | - |
| **22-88** | B–E | - | 34.2 | - | 23.7 | - | 100 |
| **24-86** | B–E | 97.5 | 50.0 | 100 | 71.1 | 100 | - |
| **45-100** | C–F | - | 2.6 | - | - | - | - |
| 47-72^a^ | C’–C’’ | - | - | - | 5.2 | - | - |
| 51-66^a^ | C’–C’’ | - | 2.6 | - | - | - | - |
| 83-91^a^ | D–E | - | 2.6 | - | - | - | - |

^a^Disulfide linkage formation not structurally plausible.

Cys pairs shown in bold were deemed structurally plausible and were introduced into sdAbs and the resulting proteins characterized. “-” means these Cys pairs were not observed following sequencing of 40 to 50 clones per panning round.

**Table S2**. **Cys pairs identified by panning the eight inter-β-sheet VH413^C23-C104 null^ Cys pair scan libraries for four rounds with heat treatment.**

| **Cys pair** | **Library** | **Round 2 (%)** | | **Round 3 (%)** | | **Round 4 (%)** | |
| --- | --- | --- | --- | --- | --- | --- | --- |
|  |  | **No heat** | **Heat (50°C)** | **No heat** | **Heat (50°C)** | **No heat** | **Heat (50°C)** |
| 4-101^a^ | A–F | n.d. | - | n.d. | - | n.d. | 2.8 |
| **4-118** | A–G | n.d. | 25 | n.d. | 61.8 | n.d. | 25 |
| **5-120** | A–G | n.d. | 5 | n.d. | - | n.d. | 2.8 |
| **6-119** | A–G | n.d. | 5 | n.d. | 29.4 | n.d. | 69.4 |
| **12-127^b^** | A–G | n.d. | 2.5 | n.d. | - | n.d. | - |
| 13-100^a^ | A–F | n.d. | 2.5 | n.d. | - | n.d. | - |
| 16-46^a^ | B–C | n.d. | 5 | n.d. | - | n.d. | - |
| 18-46^a^ | B–C | n.d. | 7.5 | n.d. | - | n.d. | - |
| 24-40^a^ | B–C | n.d. | 2.5 | n.d. | - | n.d. | - |
| 26-45^a^ | B–C | n.d. | 2.5 | n.d. | - | n.d. | - |
| 26-46^a^ | B–C | n.d. | 2.5 | n.d. | - | n.d. | - |
| 37-79^a^ | C–D | n.d. | 2.5 | n.d. | - | n.d. | - |
| 39-87^c^ | C–E | n.d. | - | n.d. | 8.8 | n.d. | - |
| 38-76^a^ | C–D | n.d. | 2.5 | n.d. | - | n.d. | - |
| 41-80^a^ | C–D | n.d. | 2.5 | n.d. | - | n.d. | - |
| **43-75^b^** | C–D | n.d. | 2.5 | n.d. | - | n.d. | - |
| 43-77^a^ | C–D | n.d. | 2.5 | n.d. | - | n.d. | - |
| 45-84^a^ | C–D | n.d. | 2.5 | n.d. | - | n.d. | - |
| 46-76^a^ | C–D | n.d. | 2.5 | n.d. | - | n.d. | - |
| 46-79^a^ | C–D | n.d. | 2.5 | n.d. | - | n.d. | - |
| 46-84^a^ | C–D | n.d. | 2.5 | n.d. | - | n.d. | - |
| 46-90^a^ | C-E | n.d. | 2.5 | n.d. | - | n.d. | - |
| 46-92^a^ | C–E | n.d. | 10 | n.d. | - | n.d. | - |
| 47-91^a^ | C’–E | n.d. | 2.5 | n.d. | - | n.d. | - |
| 47-95^a^ | C’–E | n.d. | 2.5 | n.d. | - | n.d. | - |

^a^Disulfide linkage formation not structurally plausible.

^b^While disulfide linkage formation may be possible at these positions, they were not tested further due to resource limitations.

^c^Previously known Cys39-Cys87 stabilizing disulfide linkage (Saerens *et al.*, 2008, *J. Mol. Biol.* **377**:478-488).

Cys pairs shown in bold were deemed structurally plausible and were introduced into sdAbs and the resulting proteins characterized, except for those noted with footnote b. “-” means these Cys pairs were not observed following sequencing of 40 to 50 clones per panning round.

n.d., not determined.

**Table S3**. **Physicochemical properties of wild-type and sdAbs bearing putative non-canonical disulfide linkages.**

| **sdAb type** | **sdAb** | **Disulfide linkage(s)** | ***M*_for_ (Da)^a^** | ***M*_MALS_ (Da)^b^** | **Monomer (%)^c^** | ***T*_m_ (°C)^d^** | **α-value^d^** | ***k*_a_ (1/Ms)^e^** | ***k*_d_ (1/s)^e^** | ***K*_D_ (nM)^e^** |
| --- | --- | --- | --- | --- | --- | --- | --- | --- | --- | --- |
| V_H_H | A4.2 | 23–104 | 15,727 | 15,023 | 98.0 | 87.2 ± 0.1 | 0.97 ± 0.01 | 1.74×10^6^ | 8.67×10^-3^ | 5.0 |
|  |  | 23–104, 4–25 | 15,749 | n.d. | 96.2 | 93.7 ± 0.2 | 0.66 ± 0.01 | 2.31×10^6^ | 2.00×10^-2^ | 8.7 |
|  |  | 23–104, 4–118 | 15,674 | 15,740 | 88.3 | 87.2 ± 0.1 | 0.64 ± 0.02 | 2.09×10^6^ | 1.33×10^-1^ | 63.7 |
|  |  | 23–104, 5–120 | 15,676 | n.d. | n.d. | 91.5 ± 0.1 | 0.77 ± 0.02 | n.d. | n.d. | n.d. |
|  |  | 23–104, 6–119 | 15,747 | n.d. | 90.7 | 96.5 ± 0.2 | 0.36 ± 0.02 | 8.11×10^6^ | 5.99×10^-2^ | 7.4 |
|  |  | 23–104, 22–88 | 15,683 | 16,250 | 88.7 | 89.7 ± 0.2 | 0.48 ± 0.01 | n.a. | n.a. | 61.6 |
|  |  | 23–104, 24–86 | 15,761 | 15,675 | 96.9 | 97.4 ± 0.1 | 0.65 ± 0.01 | n.a. | n.a. | 92.0 |
|  |  | 23–104, 45–100 | 15,791 | n.d. | n.d. | 91.8 ± 0.1 | 0.55 ± 0.02 | n.d. | n.d. | n.d. |
|  | A5.1 | 23–104 | 15,802 | 12,903 | 99.8 | 77.1 ± 0.1 | 0.82 ± 0.01 | 1.53×10^6^ | 7.84×10^-3^ | 5.1 |
|  |  | 23–104, 4–25 | 15,824 | n.d. | 96.4 | 86.6 ± 0.1 | 0.56 ± 0.01 | 4.15×10^6^ | 1.07×10^-2^ | 2.6 |
|  |  | 23–104, 4–118 | 15,709 | 15,313 | 88.8 | 74.9 ± 0.3 | 0.60 ± 0.01 | n.a. | n.a. | 214 |
|  |  | 23–104, 5–120 | 15,751 | n.d. | n.d. | 80.0 ± 0.4 | 0.42 ± 0.01 | n.d. | n.d. | n.d. |
|  |  | 23–104, 6–119 | 15,823 | n.d. | 98.4 | 92.0 ± 0.2 | 0.62 ± 0.01 | 4.16×10^6^ | 6.48×10^-3^ | 1.6 |
|  |  | 23–104, 22–88 | 15,758 | n.d. | 97.0 | 80.1 ± 0.2 | 0.60 ± 0.01 | 1.71×10^6^ | 8.15×10^-3^ | 4.8 |
|  |  | 23–104, 24–86 | 15,836 | 15,495 | 97.7 | 90.0 ± 0.6 | 0.48 ± 0.03 | 1.48×10^6^ | 7.74×10^-3^ | 5.2 |
|  |  | 23–104, 45–100 | 15,867 | n.d. | n.d. | 79.6 ± 0.4 | 0.15 ± 0.01 | n.d. | n.d. | n.d. |
|  | A20.1 | 23–104 | 15,714 | 16,810 | 96.2 | 73.5 ± 0.1 | 0.86 ± 0.02 | 8.52×10^5^ | 1.42×10^-3^ | 1.7 |
|  |  | 23–104, 4–25 | 15,736 | n.d. | 98.6 | 82.5 ± 0.2 | 0.58 ± 0.02 | 2.30×10^6^ | 3.53×10^-3^ | 1.5 |
|  |  | 23–104, 4–118 | 15,577 | 14,732 | 75.1 | 69.3 ± 0.4 | 0.78 ± 0.02 | n.d. | n.d. | n.d. |
|  |  | 23–104, 5–120 | 15,693 | n.d. | n.d. | 74.9 ± 0.1 | 0.47 ± 0.01 | n.d. | n.d. | n.d. |
|  |  | 23–104, 6–119 | 15,734 | 14,480 | 85.3 | 89.1 ± 0.1 | 0.32 ± 0.01 | 2.55×10^6^ | 1.60×10^-2^ | 6.3 |
|  |  | 23–104, 22–88 | 15,626 | n.d. | n.d. | n.d. | n.d. | n.d. | n.d. | n.d. |
|  |  | 23–104, 24–86 | 15,704 | 14,865 | 98.9 | 88.2 ± 0.2 | 0.44 ± 0.05 | 1.25×10^6^ | 2.10×10^-3^ | 1.7 |
|  |  | 23–104, 45–100 | 15,752 | n.d. | n.d. | 77.5 ± 0.1 | 0.35 ± 0.03 | n.d. | n.d. | n.d. |
|  | A24.1 | 23–104 | 15,767 | 16,233 | 95.7 | 76.3 ± 0.1 | 0.85 ± 0.03 | 2.98×10^4^ | 4.51×10^-3^ | 151 |
|  |  | 23–104, 4–25 | 15,789 | 14,480 | 97.9 | 87.0 ± 0.1 | 0.31 ± 0.02 | 4.68×10^3^ | 4.98×10^-3^ | 1060 |
|  |  | 23–104, 4–118 | 15,674 | 15,740 | 82.1 | 79.2 ± 0.1 | 0.73 ± 0.01 | 1.88×10^3^ | 4.02×10^-3^ | 2140 |
|  |  | 23–104, 5–120 | 15,746 | n.d. | n.d. | 89.4 ± 0.1 | 0.48 ± 0.01 | n.d. | n.d. | n.d. |
|  |  | 23–104, 6–119 | 15,787 | n.d. | 58.8 | 76.2 ± 0.4 | 0.51 ± 0.02 | 3.76×10^4^ | 1.55×10^-2^ | 413 |
|  |  | 23–104, 22–88 | 15,723 | 16,030 | 81.8 | 79.0 ± 0.2 | 0.69 ± 0.01 | 4.28×10^4^ | 2.77×10^-2^ | 647 |
|  |  | 23–104, 24–86 | 15,801 | 15,430 | 89.5 | 85.7 ± 0.1 | 0.48 ± 0.01 | 7.42×10^3^ | 2.63×10^-3^ | 355 |
|  |  | 23–104, 45–100 | 15,805 | n.d. | n.d. | 78.6 ± 0.1 | 0.35 ± 0.04 | n.d. | n.d. | n.d. |
|  | A26.8 | 23–104 | 16,016 | 16,413 | 99.2 | 83.5 ± 0.3 | n.d. | 1.57×10^6^ | 2.59×10^-2^ | 16.5 |
|  |  | 23–104, 4–25 | 16,039 | n.d. | 97.1 | 91.7 ± 0.1 | 0.71 ± 0.01 | 4.93×10^6^ | 6.20×10^-2^ | 12.6 |
|  |  | 23–104, 4–118 | 15,924 | 14,725 | 97.3 | 92.7 ± 0.2 | 0.81 ± 0.01 | n.a. | n.a. | 43.4 |
|  |  | 23–104, 5–120 | 15,966 | n.d. | n.d. | 91.1 | n.d. | n.d. | n.d. | n.d. |
|  |  | 23–104, 6–119 | 16,037 | n.d. | n.d. | 101.1 ± 0.1 | n.d. | 5.50×10^6^ | 6.91×10^-2^ | 12.6 |
|  |  | 23–104, 22–88 | 15,973 | 19,270 | 75.1 | 86.4 ± 0.2 | 0.70 ± 0.01 | 1.50×10^6^ | 2.80×10^-2^ | 18.7 |
|  |  | 23–104, 24–86 | 16,051 | 16,205 | 85.1 | 95.1 ± 0.2 | n.d. | 6.21×10^5^ | 2.71×10^-2^ | 43.6 |
|  |  | 23–104, 45–100 | 16,081 | n.d. | n.d. | 85.0 ± 0.1 | 0.65 ± 0.01 | n.d. | n.d. | n.d. |
|  | B39 | 23–104 | 15,608 | 12,785 | 96.9 | 83.3 ± 0.7 | 0.76 ± 0.01 | 3.44×10^7^ | 2.56×10^-3^ | 0.074 |
|  |  | 23–104, 4–25 | 15,630 | n.d. | n.d. | 91.3 ± 0.1 | 0.70 ± 0.01 | 1.14×10^7^ | 2.60×10^-4^ | 0.023 |
|  |  | 23–104, 4–118 | 15,515 | 15,470 | 98.5 | 80.2 ± 0.6 | 0.63 ± 0.01 | n.d. | n.d. | n.d. |
|  |  | 23–104, 5–120 | 15,658 | n.d. | n.d. | 82.0 ± 0.1 | 0.44 ± 0.02 | n.d. | n.d. | n.d. |
|  |  | 23–104, 6–119 | 15,628 | n.d. | n.d. | 92.8 ± 1.0 | 0.57 ± 0.02 | 1.56×10^7^ | 4.29×10^-4^ | 0.028 |
|  |  | 23–104, 22–88 | 15,664 | n.d. | 97.0 | 91.1 ± 0.1 | 0.71 ± 0.01 | n.d. | n.d. | n.d. |
|  |  | 23–104, 24–86 | 15,642 | 12,950 | 96.5 | 97.5 | 0.77 | n.d. | n.d. | n.d. |
|  |  | 23–104, 45–100 | 15,672 | n.d. | n.d. | 84.5 ± 0.1 | 0.16 ± 0.05 | n.d. | n.d. | n.d. |
|  | IGF1R3 | 23–104 | 16,215 | 15,275 | 99.4 | 76.7 ± 0.6 | n.d. | 2.84×10^5^ | 2.60×10^-4^ | 0.9 |
|  |  | 23–104, 4–25 | 16,237 | n.d. | 94.6 | 85.5 ± 0.1 | n.d. | 3.47×10^5^ | 7.98×10^-4^ | 2.3 |
|  |  | 23–104, 4–118 | 16,122 | 15,060 | 95.3 | 79.9 | 0.52 | n.d. | n.d. | n.d. |
|  |  | 23–104, 5–120 | 16,164 | n.d. | n.d. | 76.4 ± 0.1 | 0.42 ± 0.01 | n.d. | n.d. | n.d. |
|  |  | 23–104, 6–119 | 16,235 | n.d. | 76.0 | 88.9 ± 0.5 | 0.43 ± 0.01 | 5.18×10^5^ | 7.01×10^-4^ | 1.4 |
|  |  | 23–104, 22–88 | 16,219 | 19,140 | 96.1 | 85.5 ± 0.6 | 0.63 ± 0.01 | 2.85×10^5^ | 2.30×10^-4^ | 0.8 |
|  |  | 23–104, 24–86 | 16,221 | 15,373 | 98.1 | 88.5 ± 0.1 | 0.67 ± 0.01 | 3.53×10^5^ | 1.85×10^-3^ | 5.3 |
|  |  | 23–104, 45–100 | 16,279 | n.d. | n.d. | 76.0 ± 0.2 | n.d. | n.d. | n.d. | n.d. |
|  | IGF1R4 | 23–104 | 15,733 | 14,530 | 99.2 | 80.1 ± 0.1 | 0.79 ± 0.01 | 1.15×10^6^ | 3.26×10^-4^ | 0.3 |
|  |  | 23–104, 4–25 | 15,727 | n.d. | 94.6 | 87.4 ± 0.1 | 0.39 ± 0.01 | 2.07×10^6^ | 1.04×10^-3^ | 0.5 |
|  |  | 23–104, 4–118 | 15,640 | 16,548 | 75.6 | n.d. | n.d. | n.d. | n.d. | n.d. |
|  |  | 23–104, 5–120 | 15,682 | n.d. | n.d. | 82.3 ± 0.1 | 0.51 ± 0.03 | n.d. | n.d. | n.d. |
|  |  | 23–104, 6–119 | 15,754 | n.d. | 97.9 | 91.7 ± 0.4 | 0.73 ± 0.01 | 2.40×10^6^ | 6.20×10^-4^ | 0.3 |
|  |  | 23–104, 22–88 | 15,689 | 14,338 | 98.0 | 85.1 ± 0.8 | 0.70 ± 0.02 | 1.02×10^6^ | 5.40×10^-4^ | 0.5 |
|  |  | 23–104, 24–86 | 15,709 | 15,795 | 97.6 | 95.6 ± 0.1 | 0.69 ± 0.01 | 9.55×10^5^ | 1.02×10^-3^ | 1.1 |
|  |  | 23–104, 45–100 | 15,798 | n.d. | n.d. | 83.4 ± 0.1 | 0.44 ± 0.01 | n.d. | n.d. | n.d. |
|  | IGF1R5 | 23–104 | 15,747 | 13,603 | 99.6 | 61.9 ± 0.7 | 0.64 ± 0.02 | 4.66×10^5^ | 4.91×10^-4^ | 1.1 |
|  |  | 23–104, 4–25 | 15,769 | n.d. | 98.8 | 73.3 ± 0.1 | 0.48 ± 0.02 | 9.89×10^5^ | 7.47×10^-4^ | 0.8 |
|  |  | 23–104, 4–118 | 15,654 | 14,025 | 99.3 | 66.3 ± 0.1 | 0.47 ± 0.01 | 4.27×10^5^ | 1.99×10^-3^ | 4.7 |
|  |  | 23–104, 5–120 | 15,696 | n.d. | n.d. | 69.4 ± 0.1 | 0.41 ± 0.01 | n.d. | n.d. | n.d. |
|  |  | 23–104, 6–119 | 15,767 | n.d. | 98.2 | 78.5 ± 1.0 | 0.41 ± 0.01 | 8.96×10^5^ | 3.36×10^-4^ | 0.5 |
|  |  | 23–104, 22–88 | 15,703 | 14,360 | 99.1 | 67.1 ± 0.1 | 0.38 ± 0.01 | 4.11×10^5^ | 5.58×10^-4^ | 1.4 |
|  |  | 23–104, 24–86 | 15,781 | n.d. | n.d. | n.d. | n.d. | n.d. | n.d. | n.d. |
|  |  | 23–104, 45–100 | 15,811 | n.d. | n.d. | 65.8 ± 0.2 | 0.46 ± 0.01 | n.d. | n.d. | n.d. |
|  | EG2 | 23–104 | 16,097 | 15,545 | 94.9 | 78.9 ± 0.9 | 0.89 ± 0.03 | 1.21×10^6^ | 1.77×10^-2^ | 14.6 |
|  |  | 23–104, 4–25 | 16,119 | 14,335 | 90.4 | 88.4 ± 0.1 | 0.34 ± 0.01 | 1.91×10^6^ | 2.03×10^-2^ | 10.6 |
|  |  | 23–104, 4–118 | 16,004 | 15,797 | 88.1 | 83.8 ± 0.7 | 0.67 ± 0.07 | n.d. | n.d. | n.d. |
|  |  | 23–104, 5–120 | 16,046 | n.d. | n.d. | 80.1 ± 0.3 | 0.49 ± 0.01 | n.d. | n.d. | n.d. |
|  |  | 23–104, 6–119 | 16,117 | n.d. | 98.2 | 82.7 ± 0.5 | 0.69 ± 0.02 | 3.46×10^6^ | 8.88×10^-2^ | 25.7 |
|  |  | 23–104, 22–88 | 16,053 | 14,695 | 99.0 | 89.5 ± 0.7 | 0.72 ± 0.05 | 5.96×10^5^ | 2.82×10^-2^ | 47.2 |
|  |  | 23–104, 24–86 | 16,101 | 14,073 | 95.2 | 91.6 ± 0.5 | 0.54 ± 0.04 | 5.70×10^5^ | 3.56×10^-2^ | 62.5 |
|  |  | 23–104, 45–100 | 16,161 | n.d. | n.d. | 82.4 ± 0.2 | 0.50 ± 0.02 | n.d. | n.d. | n.d. |
|  | FC5 | 23–104 | 15,514 | 16,990 | 95.6 | 66.1 ± 0.6 | 0.60 ± 0.01 | n.d | n.d | n.d |
|  |  | 23–104, 4–25 | 15,536 | n.d. | 97.8 | 77.8 ± 0.1 | 0.51 ± 0.01 | n.d. | n.d. | n.d. |
|  |  | 23–104, 4–118 | 15,421 | n.d. | n.d. | n.d. | n.d. | n.d. | n.d. | n.d. |
|  |  | 23–104, 5–120 | 15,464 | n.d. | n.d. | 73.6 ± 0.1 | 0.62 ± 0.01 | n.d. | n.d. | n.d. |
|  |  | 23–104, 6–119 | 15,592 | n.d. | 98.4 | 77.4 ± 0.1 | 0.51 ± 0.01 | n.d. | n.d. | n.d. |
|  |  | 23–104, 22–88 | 15,470 | 16,720 | 99.0 | 70.1 ± 0.6 | 0.58 ± 0.02 | n.d. | n.d. | n.d. |
|  |  | 23–104, 24–86 | 15,548 | 13,540 | 95.7 | 81.6 ± 0.4 | n.d. | n.d | n.d | n.d |
|  |  | 23–104, 45–100 | 15,578 | n.d. | n.d. | 69.6 ± 0.1 | 0.30 ± 0.02 | n.d. | n.d. | n.d. |
| V_H_ | VH413 | 23–104 | 15,040 | n.d. | n.d. | 54.2 | n.d. | n.d. | n.d. | n.d. |
|  |  | 23–104, 4–25 | 15,062 | n.d. | 80.7 | 73.5 ± 0.2 | n.d. | n.d | n.d | n.d |
|  |  | 23–104, 4–118 | 15,084 | n.d. | n.d | n.d. | n.d. | n.d. | n.d. | n.d. |
|  |  | 23–104, 5–120 | 15,156 | n.d. | n.d | n.d. | n.d. | n.d. | n.d. | n.d. |
|  |  | 23–104, 6–119 | 15,198 | n.d. | 90.1 | 86.2 ± 0.3 | n.d. | n.d. | n.d. | n.d. |
|  |  | 23–104, 22–88 | 15,133 | n.d. | n.d. | 72.7 | n.d. | n.d. | n.d. | n.d. |
|  |  | 23–104, 24–86 | 15,074 | n.d. | n.d. | 79.1 | n.d. | n.d. | n.d. | n.d. |
|  |  | 23–104, 45–100 | 15,241 | n.d. | n.d | n.d. | n.d. | n.d. | n.d. | n.d. |
|  | VH414 | 23–104 | 14,968 | n.d. | 79.5 | 58.7 ± 0.1 | n.d. | n.d. | n.d. | n.d. |
|  |  | 23–104, 4–25 | 15,127 | n.d. | 48.7 | 72.9 ± 0.1 | n.d. | n.d | n.d | n.d |
|  |  | 23–104, 4–118 | 15,012 | n.d. | n.d | n.d. | n.d. | n.d. | n.d. | n.d. |
|  |  | 23–104, 5–120 | 15,084 | n.d. | n.d | n.d. | n.d. | n.d. | n.d. | n.d. |
|  |  | 23–104, 6–119 | 15,126 | n.d. | 79.2 | 79.1 ± 0.6 | n.d. | n.d. | n.d. | n.d. |
|  |  | 23–104, 22–88 | 15,061 | n.d. | n.d. | n.d. | n.d. | n.d. | n.d. | n.d. |
|  |  | 23–104, 24–86 | 14,972 | 12,945 | 97.4 | 69.6 ± 0.1 | n.d. | n.d. | n.d. | n.d. |
|  |  | 23–104, 45–100 | 15,169 | n.d. | n.d | n.d. | n.d. | n.d. | n.d. | n.d. |
|  | VH419 | 23–104 | 14,947 | n.d. | n.d. | 58.9 ± 0.2 | 0.35 ± 0.03 | n.d. | n.d. | n.d. |
|  |  | 23–104, 4–25 | 15,106 | n.d. | 93.3 | 73.2 ± 0.3 | n.d. | n.d | n.d | n.d |
|  |  | 23–104, 4–118 | 14,991 | n.d. | n.d | n.d. | n.d. | n.d. | n.d. | n.d. |
|  |  | 23–104, 5–120 | 15,034 | n.d. | n.d | n.d. | n.d. | n.d. | n.d. | n.d. |
|  |  | 23–104, 6–119 | 15,105 | 13,180 | 84.8 | 73.5 ± 0.6 | n.d. | n.d. | n.d. | n.d. |
|  |  | 23–104, 22–88 | 15,040 | n.d. | n.d. | n.d. | n.d. | n.d. | n.d. | n.d. |
|  |  | 23–104, 24–86 | 14,995 | 13,515 | 98.6 | 70.8 ± 0.1 | 0.30 ± 0.05 | n.d. | n.d. | n.d. |
|  |  | 23–104, 45–100 | 15,149 | n.d. | n.d | n.d. | n.d. | n.d. | n.d. | n.d. |
|  | VH420 | 23–104 | 14,932 | n.d. | 78.5 | 58.1 ± 0.1 | n.d. | n.d. | n.d. | n.d. |
|  |  | 23–104, 4–25 | 15,091 | n.d. | 86.0 | 73.8 ± 0.1 | n.d. | n.d | n.d | n.d |
|  |  | 23–104, 4–118 | 14,976 | n.d. | n.d | n.d. | n.d. | n.d. | n.d. | n.d. |
|  |  | 23–104, 5–120 | 15,048 | n.d. | n.d | n.d. | n.d. | n.d. | n.d. | n.d. |
|  |  | 23–104, 6–119 | 15,089 | 15,665 | 70.9 | 77.4 ± 0.2 | n.d. | n.d. | n.d. | n.d. |
|  |  | 23–104, 22–88 | 14,881 | n.d. | n.d. | n.d. | n.d. | n.d. | n.d. | n.d. |
|  |  | 23–104, 24–86 | 14,966 | 12,820 | 96.3 | 69.3 ± 0.2 | n.d. | n.d. | n.d. | n.d. |
|  |  | 23–104, 45–100 | 15,133 | n.d. | n.d | n.d. | n.d. | n.d. | n.d. | n.d. |
|  | VH421 | 23–104 | 15,392 | n.d. | 83.9 | 58.8 ± 0.1 | n.d. | n.d. | n.d. | n.d. |
|  |  | 23–104, 4–25 | 15,414 | n.d. | 85.7 | 74.5 ± 0.4 | n.d. | n.d | n.d | n.d |
|  |  | 23–104, 4–118 | 15,299 | n.d. | n.d | n.d. | n.d. | n.d. | n.d. | n.d. |
|  |  | 23–104, 5–120 | 15,342 | n.d. | n.d | n.d. | n.d. | n.d. | n.d. | n.d. |
|  |  | 23–104, 6–119 | 15,412 | n.d. | 76.0 | 78.3 | n.d. | n.d. | n.d. | n.d. |
|  |  | 23–104, 22–88 | 15,348 | n.d. | 93.6 | n.d. | n.d. | n.d. | n.d. | n.d. |
|  |  | 23–104, 24–86 | 15,289 | 15,580 | 94.6 | 69.4 ± 0.1 | n.d. | n.d. | n.d. | n.d. |
|  |  | 23–104, 45–100 | 15,456 | n.d. | n.d | n.d. | n.d. | n.d. | n.d. | n.d. |
|  | VH423 | 23–104 | 14,966 | n.d. | n.d. | 57.0 ± 1.0 | n.d. | n.d. | n.d. | n.d. |
|  |  | 23–104, 4–25 | 15,125 | n.d. | 60.4 | 66.5 ± 0.7 | n.d. | n.d | n.d | n.d |
|  |  | 23–104, 4–118 | 15,010 | n.d. | n.d | n.d. | n.d. | n.d. | n.d. | n.d. |
|  |  | 23–104, 5–120 | 15,082 | n.d. | n.d | n.d. | n.d. | n.d. | n.d. | n.d. |
|  |  | 23–104, 6–119 | 15,124 | n.d. | 93.5 | 82.2 ± 0.1 | n.d. | n.d. | n.d. | n.d. |
|  |  | 23–104, 22–88 | 15,059 | n.d. | n.d. | n.d. | n.d. | n.d. | n.d. | n.d. |
|  |  | 23–104, 24–86 | 15,000 | 14,075 | 94.3 | 76.0 ± 0.1 | n.d. | n.d. | n.d. | n.d. |
|  |  | 23–104, 45–100 | 15,167 | n.d. | n.d | n.d. | n.d. | n.d. | n.d. | n.d. |
|  | VH428 | 23–104 | 15,675 | n.d. | 94.4 | 62.3 | n.d. | n.d. | n.d. | n.d. |
|  |  | 23–104, 4–25 | 15,697 | n.d. | 67.7 | 76.5 ± 0.2 | n.d. | n.d | n.d | n.d |
|  |  | 23–104, 4–118 | 15,582 | n.d. | n.d | n.d. | n.d. | n.d. | n.d. | n.d. |
|  |  | 23–104, 5–120 | 15,625 | n.d. | n.d | n.d. | n.d. | n.d. | n.d. | n.d. |
|  |  | 23–104, 6–119 | 15,695 | 15,430 | 84.7 | 83.2 ± 0.2 | n.d. | n.d. | n.d. | n.d. |
|  |  | 23–104, 22–88 | 15,631 | n.d. | 86.8 | 64.6 | n.d. | n.d. | n.d. | n.d. |
|  |  | 23–104, 24–86 | 15,560 | n.d. | n.d. | 77.4 | n.d. | n.d. | n.d. | n.d. |
|  |  | 23–104, 45–100 | 15,739 | n.d. | n.d | n.d. | n.d. | n.d. | n.d. | n.d. |
|  | VH429 | 23–104 | 14,959 | n.d. | 88.0 | 59.9 ± 0.1 | 0.48 ± 0.01 | n.d. | n.d. | n.d. |
|  |  | 23–104, 4–25 | 15,119 | n.d. | 83.7 | 70.1 ± 0.1 | n.d. | n.d | n.d | n.d |
|  |  | 23–104, 4–118 | 15,004 | n.d. | n.d | n.d. | n.d. | n.d. | n.d. | n.d. |
|  |  | 23–104, 5–120 | 15,076 | n.d. | n.d | n.d. | n.d. | n.d. | n.d. | n.d. |
|  |  | 23–104, 6–119 | 15,117 | n.d. | n.d. | n.d. | n.d. | n.d. | n.d. | n.d. |
|  |  | 23–104, 22–88 | 15,053 | n.d. | n.d. | n.d. | n.d. | n.d. | n.d. | n.d. |
|  |  | 23–104, 24–86 | 14,994 | 13,570 | 96.1 | 65.2 ± 0.1 | 0.26 ± 0.01 | n.d. | n.d. | n.d. |
|  |  | 23–104, 45–100 | 15,161 | n.d. | n.d | n.d. | n.d. | n.d. | n.d. | n.d. |
|  | VHM41 | 23–104 | 15,333 | n.d. | 47.5 | 52.8 ± 2.2 | n.d. | n.d. | n.d. | n.d. |
|  |  | 23–104, 4–25 | 15,492 | n.d. | 29.8 | 77.4 ± 0.1 | n.d. | n.d | n.d | n.d |
|  |  | 23–104, 4–118 | 15,377 | n.d. | n.d | n.d. | n.d. | n.d. | n.d. | n.d. |
|  |  | 23–104, 5–120 | 15,449 | n.d. | n.d | n.d. | n.d. | n.d. | n.d. | n.d. |
|  |  | 23–104, 6–119 | 15,491 | n.d. | 70.0 | 79.5 ± 0.1 | n.d. | n.d. | n.d. | n.d. |
|  |  | 23–104, 22–88 | 15,426 | n.d. | n.d. | n.d. | n.d. | n.d. | n.d. | n.d. |
|  |  | 23–104, 24–86 | 15,367 | 15,220 | 76.4 | 75.0 ± 0.3 | n.d. | n.d. | n.d. | n.d. |
|  |  | 23–104, 45–100 | 15,534 | n.d. | n.d | n.d. | n.d. | n.d. | n.d. | n.d. |
|  | VH44 | 23–104 | 15,274 | n.d. | 92.8 | 67.9 ± 0.1 | 0.51 ± 0.01 | n.d. | n.d. | n.d. |
|  |  | 23–104, 4–25 | 15,433 | n.d. | 75.7 | 80.9 ± 0.1 | n.d. | n.d | n.d | n.d |
|  |  | 23–104, 4–118 | 15,318 | n.d. | n.d | n.d. | n.d. | n.d. | n.d. | n.d. |
|  |  | 23–104, 5–120 | 15,361 | n.d. | n.d | n.d. | n.d. | n.d. | n.d. | n.d. |
|  |  | 23–104, 6–119 | 15,431 | n.d. | 63.5 | 79.7 ± 0.1 | n.d. | n.d. | n.d. | n.d. |
|  |  | 23–104, 22–88 | 15,367 | n.d. | 72.1 | n.d. | n.d. | n.d. | n.d. | n.d. |
|  |  | 23–104, 24–86 | 15,308 | 13,910 | 94.1 | 76.3 ± 0.2 | 0.40 ± 0.02 | n.d. | n.d. | n.d. |
|  |  | 23–104, 45–100 | 15,475 | n.d. | n.d | n.d. | n.d. | n.d. | n.d. | n.d. |
|  | VHM81 | 23–104 | 15,058 | n.d. | 94.7 | 69.6 ± 0.4 | n.d. | n.d. | n.d. | n.d. |
|  |  | 23–104, 4–25 | 15,217 | n.d. | 77.4 | 81.0 ± 0.1 | n.d. | n.d | n.d | n.d |
|  |  | 23–104, 4–118 | 15,102 | n.d. | n.d | n.d. | n.d. | n.d. | n.d. | n.d. |
|  |  | 23–104, 5–120 | 15,174 | n.d. | n.d | n.d. | n.d. | n.d. | n.d. | n.d. |
|  |  | 23–104, 6–119 | 15,216 | n.d. | 78.7 | 84.4 ± 0.1 | n.d. | n.d. | n.d. | n.d. |
|  |  | 23–104, 22–88 | 15,151 | 16,910 | 85.2 | n.d. | n.d. | n.d. | n.d. | n.d. |
|  |  | 23–104, 24–86 | 15,092 | 15,330 | 97.2 | 80.2 ± 0.1 | n.d. | n.d. | n.d. | n.d. |
|  |  | 23–104, 45–100 | 15,259 | n.d. | n.d | n.d. | n.d. | n.d. | n.d. | n.d. |
|  | VHB82 | 23–104 | 14,141 | n.d. | 53.2 | 60.7 ± 0.1 | 0.37 ± 0.01 | n.d. | n.d. | n.d. |
|  |  | 23–104, 4–25 | 14,230 | 12,375 | 89.4 | 83.5 ± 0.1 | n.d. | n.d | n.d | n.d |
|  |  | 23–104, 4–118 | 14,185 | n.d. | n.d | n.d. | n.d. | n.d. | n.d. | n.d. |
|  |  | 23–104, 5–120 | 14,228 | n.d. | n.d | n.d. | n.d. | n.d. | n.d. | n.d. |
|  |  | 23–104, 6–119 | 14,298 | n.d. | 97.0 | 75.7 ± 0.2 | n.d. | n.d. | n.d. | n.d. |
|  |  | 23–104, 22–88 | 14,234 | n.d. | n.d. | n.d. | n.d. | n.d. | n.d. | n.d. |
|  |  | 23–104, 24–86 | 14,175 | 14,190 | 82.7 | 75.6 ± 0.1 | 0.36 ± 0.01 | n.d. | n.d. | n.d. |
|  |  | 23–104, 45–100 | 14,342 | n.d. | n.d | n.d. | n.d. | n.d. | n.d. | n.d. |
| V_L_ | VL324 | 23–104 | 13,832 | 13,180 | 68.6 | 66.1 | n.d. | n.d. | n.d. | n.d. |
|  |  | 23–104, 4–25 | 13,836 | n.d. | 93.1 | 81.7 ± 0.4 | n.d. | n.d | n.d | n.d |
|  |  | 23–104, 4–118 | 13,760 | n.d. | n.d | n.d. | n.d. | n.d. | n.d. | n.d. |
|  |  | 23–104, 5–120 | 13,800 | n.d. | n.d | n.d. | n.d. | n.d. | n.d. | n.d. |
|  |  | 23–104, 6–119 | 13,853 | n.d. | 79.9 | 81.1 | n.d. | n.d. | n.d. | n.d. |
|  |  | 23–104, 22–88 | 13,836 | n.d. | 100.0 | n.d. | n.d. | n.d. | n.d. | n.d. |
|  |  | 23–104, 24–86 | 13,767 | 12,510 | 98.0 | n.d. | n.d. | n.d. | n.d. | n.d. |
|  |  | 23–104, 45–100 | 13,839 | n.d. | n.d | n.d. | n.d. | n.d. | n.d. | n.d. |
|  | VL325 | 23–104 | 13,608 | 13,205 | 80.9 | 70.8 | 0.86 ± 0.01 | n.d. | n.d. | n.d. |
|  |  | 23–104, 4–25 | 13,630 | 13,475 | 97.2 | 79.4 ± 0.6 | n.d. | n.d | n.d | n.d |
|  |  | 23–104, 4–118 | 13,554 | n.d. | n.d | n.d. | n.d. | n.d. | n.d. | n.d. |
|  |  | 23–104, 5–120 | 13,656 | n.d. | n.d | n.d. | n.d. | n.d. | n.d. | n.d. |
|  |  | 23–104, 6–119 | 13,629 | n.d. | n.d. | n.d. | n.d. | n.d. | n.d. | n.d. |
|  |  | 23–104, 22–88 | 13,626 | n.d. | n.d. | n.d. | n.d. | n.d. | n.d. | n.d. |
|  |  | 23–104, 24–86 | 13,545 | 14,230 | 100.0 | 76.8 ± 0.1 | 0.79 ± 0.01 | n.d. | n.d. | n.d. |
|  |  | 23–104, 45–100 | 13,587 | n.d. | n.d | n.d. | n.d. | n.d. | n.d. | n.d. |
|  | VL330 | 23–104 | 13,572 | 13,825 | 81.6 | 63.8 | 0.85 ± 0.01 | n.d. | n.d. | n.d. |
|  |  | 23–104, 4–25 | 13,576 | n.d. | 87.8 | 80.6 ± 1.1 | n.d. | n.d | n.d | n.d |
|  |  | 23–104, 4–118 | 13,500 | n.d. | n.d | n.d. | n.d. | n.d. | n.d. | n.d. |
|  |  | 23–104, 5–120 | 13,540 | n.d. | n.d | n.d. | n.d. | n.d. | n.d. | n.d. |
|  |  | 23–104, 6–119 | 13,593 | n.d. | 91.5 | 79.4 ± 0.1 | n.d. | n.d. | n.d. | n.d. |
|  |  | 23–104, 22–88 | 13,576 | n.d. | n.d. | n.d. | n.d. | n.d. | n.d. | n.d. |
|  |  | 23–104, 24–86 | 13,509 | 10,525 | 93.2 | 66.0 ± 0.1 | 0.90 ± 0.01 | n.d. | n.d. | n.d. |
|  |  | 23–104, 45–100 | 13,549 | n.d. | n.d | n.d. | n.d. | n.d. | n.d. | n.d. |
|  | VL335 | 23–104 | 13,904 | n.d. | n.d. | 63.5 | 0.88 ± 0.01 | n.d. | n.d. | n.d. |
|  |  | 23–104, 4–25 | 13,908 | n.d. | 94.6 | 78.8 ± 1.0 | n.d. | n.d | n.d | n.d |
|  |  | 23–104, 4–118 | 13,832 | n.d. | n.d | n.d. | n.d. | n.d. | n.d. | n.d. |
|  |  | 23–104, 5–120 | 13,881 | n.d. | n.d | n.d. | n.d. | n.d. | n.d. | n.d. |
|  |  | 23–104, 6–119 | 13,925 | n.d. | 76.1 | 76.6 ± 0.4 | n.d. | n.d. | n.d. | n.d. |
|  |  | 23–104, 22–88 | 13,922 | n.d. | 97.5 | 71.0 ± 1.2 | n.d. | n.d. | n.d. | n.d. |
|  |  | 23–104, 24–86 | 13,817 | n.d. | n.d. | 69.8 ± 0.2 | 0.70 ± 0.02 | n.d. | n.d. | n.d. |
|  |  | 23–104, 45–100 | 13,911 | n.d. | n.d | n.d. | n.d. | n.d. | n.d. | n.d. |
|  | VL342 | 23–104 | 13,950 | n.d. | n.d. | 58.4 | n.d. | n.d. | n.d. | n.d. |
|  |  | 23–104, 4–25 | 13,938 | n.d. | 94.1 | 78.9 ± 0.3 | n.d. | n.d | n.d | n.d |
|  |  | 23–104, 4–118 | 13,878 | n.d. | n.d | n.d. | n.d. | n.d. | n.d. | n.d. |
|  |  | 23–104, 5–120 | 13,942 | n.d. | n.d | n.d. | n.d. | n.d. | n.d. | n.d. |
|  |  | 23–104, 6–119 | 13,972 | n.d. | n.d. | n.d. | n.d. | n.d. | n.d. | n.d. |
|  |  | 23–104, 22–88 | 13,899 | n.d. | 76.3 | 61.0 ± 0.8 | n.d. | n.d. | n.d. | n.d. |
|  |  | 23–104, 24–86 | 13,871 | n.d. | n.d. | n.d. | n.d. | n.d. | n.d. | n.d. |
|  |  | 23–104, 45–100 | 13,929 | n.d. | n.d | n.d. | n.d. | n.d. | n.d. | n.d. |
|  | VL351 | 23–104 | 13,854 | 14,800 | 91.8 | 62.0 | n.d. | n.d. | n.d. | n.d. |
|  |  | 23–104, 4–25 | 13,858 | n.d. | 93.8 | 73.6 ± 0.4 | n.d. | n.d | n.d | n.d |
|  |  | 23–104, 4–118 | 13,782 | n.d. | n.d | n.d. | n.d. | n.d. | n.d. | n.d. |
|  |  | 23–104, 5–120 | 13,902 | n.d. | n.d | n.d. | n.d. | n.d. | n.d. | n.d. |
|  |  | 23–104, 6–119 | 13,875 | n.d. | 77.4 | 74.5 ± 0.1 | n.d. | n.d. | n.d. | n.d. |
|  |  | 23–104, 22–88 | 13,872 | n.d. | 76.2 | 64.5 ± 0.7 | n.d. | n.d. | n.d. | n.d. |
|  |  | 23–104, 24–86 | 13,789 | 12,810 | 977 | 68.9 ± 0.2 | n.d. | n.d. | n.d. | n.d. |
|  |  | 23–104, 45–100 | 13,861 | n.d. | n.d | n.d. | n.d. | n.d. | n.d. | n.d. |
|  | VL364 | 23–104 | 13,945 | 13,685 | 86.0 | 58.7 | 0.84 ± 0.01 | n.d. | n.d. | n.d. |
|  |  | 23–104, 4–25 | 13,967 | n.d. | 91.8 | 70.8 ± 0.2 | n.d. | n.d | n.d | n.d |
|  |  | 23–104, 4–118 | 13,891 | n.d. | n.d | n.d. | n.d. | n.d. | n.d. | n.d. |
|  |  | 23–104, 5–120 | 13,922 | n.d. | n.d | n.d. | n.d. | n.d. | n.d. | n.d. |
|  |  | 23–104, 6–119 | 13,993 | n.d. | 98.7 | 74.3 ± 0.1 | n.d. | n.d. | n.d. | n.d. |
|  |  | 23–104, 22–88 | 13,963 | 14,360 | 81.2 | 66.1 ± 0.4 | n.d. | n.d. | n.d. | n.d. |
|  |  | 23–104, 24–86 | 13,880 | 13,220 | 98.5 | 65.5 ± 0.1 | 0.68 ± 0.01 | n.d. | n.d. | n.d. |
|  |  | 23–104, 45–100 | 13,952 | n.d. | n.d | n.d. | n.d. | n.d. | n.d. | n.d. |
|  | VL382 | 23–104 | 13,931 | 13,905 | 84.3 | 72.6 | 0.92 ± 0.01 | n.d. | n.d. | n.d. |
|  |  | 23–104, 4–25 | 13,935 | n.d. | 60.8 | 59.1 ± 0.2 | n.d. | n.d | n.d | n.d |
|  |  | 23–104, 4–118 | 13,859 | n.d. | n.d | n.d. | n.d. | n.d. | n.d. | n.d. |
|  |  | 23–104, 5–120 | 13,908 | n.d. | n.d | n.d. | n.d. | n.d. | n.d. | n.d. |
|  |  | 23–104, 6–119 | 13,953 | n.d. | 90.6 | 85.4 ± 0.3 | n.d. | n.d. | n.d. | n.d. |
|  |  | 23–104, 22–88 | 13,950 | 14,820 | 89.3 | 74.6 ± 0.1 | n.d. | n.d. | n.d. | n.d. |
|  |  | 23–104, 24–86 | 13,866 | 12,520 | 96.6 | 79.4 ± 2.1 | 0.77 ± 0.01 | n.d. | n.d. | n.d. |
|  |  | 23–104, 45–100 | 13,938 | n.d. | n.d | n.d. | n.d. | n.d. | n.d. | n.d. |
|  | VL383 | 23–104 | 13,796 | 14,665 | 76.8 | 58.9 | 0.89 ± 0.01 | n.d. | n.d. | n.d. |
|  |  | 23–104, 4–25 | 13,818 | n.d. | 90.0 | 72.4 ± 0.1 | n.d. | n.d | n.d | n.d |
|  |  | 23–104, 4–118 | 13,742 | n.d. | n.d | n.d. | n.d. | n.d. | n.d. | n.d. |
|  |  | 23–104, 5–120 | 13,773 | n.d. | n.d | n.d. | n.d. | n.d. | n.d. | n.d. |
|  |  | 23–104, 6–119 | 13,817 | n.d. | 92.2 | 74.6 ± 0.1 | n.d. | n.d. | n.d. | n.d. |
|  |  | 23–104, 22–88 | 13,814 | n.d. | 94.0 | 65.9 ± 0.1 | n.d. | n.d. | n.d. | n.d. |
|  |  | 23–104, 24–86 | 13,866 | 12,285 | 94.1 | 67.8 ± 0.1 | 0.65 ± 0.01 | n.d. | n.d. | n.d. |
|  |  | 23–104, 45–100 | 13,803 | n.d. | n.d | n.d. | n.d. | n.d. | n.d. | n.d. |
|  | VL389 | 23–104 | 13,756 | 14,040 | 89.6 | 54.5 | 0.77 ± 0.01 | n.d. | n.d. | n.d. |
|  |  | 23–104, 4–25 | 13,806 | 13,815 | 99.0 | 66.2 ± 0.1 | n.d. | n.d | n.d | n.d |
|  |  | 23–104, 4–118 | 13,716 | n.d. | n.d | n.d. | n.d. | n.d. | n.d. | n.d. |
|  |  | 23–104, 5–120 | 13,804 | n.d. | n.d | n.d. | n.d. | n.d. | n.d. | n.d. |
|  |  | 23–104, 6–119 | 13,777 | n.d. | 96.6 | 67.6 ± 0.1 | n.d. | n.d. | n.d. | n.d. |
|  |  | 23–104, 22–88 | 13,774 | n.d. | 99.8 | 67.6 | n.d. | n.d. | n.d. | n.d. |
|  |  | 23–104, 24–86 | 13,719 | 14,095 | 100.0 | 70.6 ± 1.1 | 0.83 ± 0.01 | n.d. | n.d. | n.d. |
|  |  | 23–104, 45–100 | 13,778 | n.d. | n.d | n.d. | n.d. | n.d. | n.d. | n.d. |
|  | VL3103 | 23–104 | 13,852 | 14,030 | 89.4 | 68.7 | 0.90 ± 0.01 | n.d. | n.d. | n.d. |
|  |  | 23–104, 4–25 | 13,874 | 9,980 | 59.0 | 76.5 ± 0.2 | n.d. | n.d | n.d | n.d |
|  |  | 23–104, 4–118 | 13,798 | n.d. | n.d | n.d. | n.d. | n.d. | n.d. | n.d. |
|  |  | 23–104, 5–120 | 13,829 | n.d. | n.d | n.d. | n.d. | n.d. | n.d. | n.d. |
|  |  | 23–104, 6–119 | 13,873 | n.d. | 98.7 | 83.7 ± 0.3 | n.d. | n.d. | n.d. | n.d. |
|  |  | 23–104, 22–88 | 13,870 | n.d. | 83.2 | 72.5 | n.d. | n.d. | n.d. | n.d. |
|  |  | 23–104, 24–86 | 13,787 | 12,455 | 97.4 | 74.1 ± 0.2 | 0.67 ± 0.01 | n.d. | n.d. | n.d. |
|  |  | 23–104, 45–100 | 13,831 | n.d. | n.d | n.d. | n.d. | n.d. | n.d. | n.d. |

^a^*M*_for_, formula molecular mass calculated from the sdAb amino acid sequence including c-Myc and His_6_ tags.

^b^Calculated for the monomeric sdAb peak area from SEC-MALS data.

^c^Calculated from SEC-MALS data.

^d^Mean ± SEM of technical duplicate circular dichroism measurements except for samples with limiting amounts.

^e^Measured by SPR.

n.a., not applicable because *K*_D_s were determined by steady-state analysis.

n.d., not determined.

**Table S4**. **Percent V_H_H protein undigested following 1 h incubation with pepsin at 37°C**.

| **V_H_H** | **Disulfide linkage(s)** | **Pepsin** | | |
| --- | --- | --- | --- | --- |
|  |  | **10 µg/mL**  **(%)** | **50 µg/mL**  **(%)** | **100 µg/mL**  **(%)** |
| A4.2 | 23–104 | 0 | 0 | 0 |
|  | 23–104, 4–25 | 51 ± 13 | 1 ± 1 | 0 |
|  | 23–104, 6–119 | 81 ± 3 | 76 ± 2 | 70 ± 9 |
|  | 23–104, 22–88 | 15 ± 4 | 0 | 0 |
|  | 23–104, 24–86 | 93 ± 3 | 64 ± 5 | 36 ± 7 |
| A5.1 | 23–104 | 0 | 0 | 0 |
|  | 23–104, 4–25 | 0 | 0 | 0 |
|  | 23–104, 6–119 | 73 ± 2 | 59 ± 6 | 60 ± 5 |
|  | 23–104, 22–88 | 3 ± 2 | 0 | 0 |
|  | 23–104, 24–86 | 78 ± 2 | 32 ± 5 | 11 ± 3 |
| A20.1 | 23–104 | 0 | 0 | 0 |
|  | 23–104, 4–25 | 6 ± 6 | 0 | 0 |
|  | 23–104, 6–119 | 60 ± 2 | 35 ± 3 | 30 ± 3 |
|  | 23–104, 22–88 | n.d. | n.d. | n.d. |
|  | 23–104, 24–86 | 39 ± 2 | 7 ± 5 | 3 ± 2 |
| A24.1 | 23–104 | 0 | 0 | 0 |
|  | 23–104, 4–25 | 40 ± 20 | 22 ± 8 | 11 ± 6 |
|  | 23–104, 6–119 | 98 ± 12 | 86 ± 10 | 73 ± 9 |
|  | 23–104, 22–88 | n.d. | n.d. | n.d. |
|  | 23–104, 24–86 | n.d. | n.d. | n.d. |
| A26.8 | 23–104 | 0 | 0 | 0 |
|  | 23–104, 4–25 | 21 ± 8 | 0 | 0 |
|  | 23–104, 6–119 | n.d. | n.d. | n.d. |
|  | 23–104, 22–88 | 7 ± 2 | 0 | 0 |
|  | 23–104, 24–86 | 68 ± 10 | 50 ± 6 | 31 ± 5 |
| B39 | 23–104 | 0 | 0 | 0 |
|  | 23–104, 4–25 | 35 ± 2 | 4 ± 1 | 0 |
|  | 23–104, 6–119 | n.d. | n.d. | n.d. |
|  | 23–104, 22–88 | 27 ± 2 | 0 | 0 |
|  | 23–104, 24–86 | 85 ± 1 | 52 ± 5 | 40 ± 10 |
| IGF1R3 | 23–104 | 0 | 0 | 0 |
|  | 23–104, 4–25 | 9 ± 4 | 0 | 0 |
|  | 23–104, 6–119 | 0 | 0 | 0 |
|  | 23–104, 22–88 | 0 | 0 | 0 |
|  | 23–104, 24–86 | 0 | 0 | 0 |
| IGF1R4 | 23–104 | 2 ± 1 | 0 | 0 |
|  | 23–104, 4–25 | 4 ± 2 | 2 ± 1 | 1 ± 1 |
|  | 23–104, 6–119 | 0 | 0 | 0 |
|  | 23–104, 22–88 | 3 ± 1 | 0 | 0 |
|  | 23–104, 24–86 | 0 | 0 | 0 |
| IGF1R5 | 23–104 | 0 | 0 | 0 |
|  | 23–104, 4–25 | n.d. | n.d. | n.d. |
|  | 23–104, 6–119 | 61 ± 1 | 52 ± 2 | 43 ± 4 |
|  | 23–104, 22–88 | 0 | 0 | 0 |
|  | 23–104, 24–86 | n.d. | n.d. | n.d. |
| EG2 | 23–104 | 0 | 0 | 0 |
|  | 23–104, 4–25 | 40 ± 3 | 5 ± 1 | 0 |
|  | 23–104, 6–119 | 41 ± 2 | 33 ± 2 | 23 ± 1 |
|  | 23–104, 22–88 | n.d. | n.d. | n.d. |
|  | 23–104, 24–86 | 58 ± 4 | 8 ± 5 | 1 ± 1 |
| FC5 | 23–104 | 0 | 0 | 0 |
|  | 23–104, 4–25 | 14 ± 1 | 0 | 0 |
|  | 23–104, 6–119 | 78 ± 2 | 55 ± 1 | 49 ± 2 |
|  | 23–104, 22–88 | n.d. | n.d. | n.d. |
|  | 23–104, 24–86 | 85 ± 5 | 39 ± 8 | 18 ± 4 |

Results represent the means ± SEMs of three technical replicates.

n.d., not determined.

**Table S5.** **Intact LC-MS analysis of free sulfhydryl abundance in wild-type and Cys-engineered V_H_Hs.**

| **Ig V disulfide linkages** | **Protein** | **Treatment** | **No. of labels (%)** | | | | | | **Mol SH per mol protein** |
| --- | --- | --- | --- | --- | --- | --- | --- | --- | --- |
|  |  |  | **0** | **1** | **2** | **3** | **4** | **5** |  |
| n.a. | β-lactoglobulin | Unlabeled (GdnHCl) | 100.0 | - | - | - | - | - | 0.0 |
|  |  | Nonreduced  (GdnHCl, mPEG2-biotin) | - | 99.7 | - | 0.3 | - | - | 1.0 |
|  |  | Reduced  (GdnHCl, TCEP, mPEG2-biotin) | - | 2.4 | - | 17.5 | - | 80.1 | 4.6 |
| Cys23-Cys104 | EG2 WT | Unlabeled (GdnHCl) | 100.0 | - | - | - | - | - | 0.0 |
|  |  | Nonreduced  (GdnHCl, mPEG2-biotin) | 99.8 | - | 0.2 | - | - | - | 0.0 |
|  |  | Reduced  (GdnHCl, TCEP, mPEG2-biotin) | 8.3 | - | 91.7 | - | - | - | 1.8 |
| Cys23-Cys104, Cys22-Cys88 | EG2^C22-C88^  (Δ*T*_m_ : 10.6°C) | Unlabeled (GdnHCl) | 100.0 | - | - | - | - | - | 0.0 |
|  |  | Nonreduced  (GdnHCl, mPEG2-biotin) | 94.8 | - | 5.2 | - | - | - | 0.1 |
|  |  | Reduced  (GdnHCl, TCEP, mPEG2-biotin) | 8.8 | - | 28.3 | - | 62.9 | - | 3.1 |
|  | IGF1R3^C22-C88^  (Δ*T*_m_: 8.8°C) | Unlabeled (GdnHCl) | 100.0 | - | - | - | - | - | 0.0 |
|  |  | Nonreduced  (GdnHCl, mPEG2-biotin) | 87.3 | - | 12.7 | - | - | - | 0.3 |
|  |  | Reduced  (GdnHCl, TCEP, mPEG2-biotin) | 16.5 | 0.2 | 25.7 | 0.4 | 57.3 | - | 2.8 |
|  | FC5^C22-C88^  (Δ*T*_m_: 4.0°C) | Unlabeled (GdnHCl) | 100.0 | - | - | - | - | - | 0.0 |
|  |  | Nonreduced  (GdnHCl, mPEG2-biotin) | 98.9 | - | 1.0 | - | - | - | 0.0 |
|  |  | Reduced  (GdnHCl, TCEP, mPEG2-biotin) | 20.6 | - | 29.8 | - | 49.7 | - | 2.6 |
|  | A5.1^C22-C88^  (Δ*T*_m_: 3.0°C) | Unlabeled (GdnHCl) | 100.0 | - | - | - | - | - | 0.0 |
|  |  | Nonreduced  (GdnHCl, mPEG2-biotin) | 97.8 | - | 2.2 | - | - | - | 0.0 |
|  |  | Reduced  (GdnHCl, TCEP, mPEG2-biotin) | 5.9 | - | 27.5 | - | 66.6 | - | 3.2 |
| Cys23-Cys104, Cys24-Cys86 | EG2^C24-C86^  (Δ*T*_m_: 12.7°C) | Unlabeled (GdnHCl) | 99.9 | - | 0.1 | - | - | - | 0.0 |
|  |  | Nonreduced  (GdnHCl, mPEG2-biotin) | 95.4 | - | 4.5 | - | - | - | 0.1 |
|  |  | Reduced  (GdnHCl, TCEP, mPEG2-biotin) | 3.7 | 0.1 | 24.9 | - | 71.3 | - | 3.4 |
|  | IGF1R3^C24-C86^  (Δ*T*_m_: 11.8°C) | Unlabeled (GdnHCl) | 100.0 | - | - | - | - | - | 0.0 |
|  |  | Nonreduced  (GdnHCl, mPEG2-biotin) | 96.5 | - | 3.5 | - | - | - | 0.1 |
|  |  | Reduced  (GdnHCl, TCEP, mPEG2-biotin) | 12.2 | - | 34.0 | - | 53.8 | - | 2.8 |
| Cys23-Cys104, Cys4-Cys25 | FC5^C4-C25^  (Δ*T*_m_: 11.7°C) | Unlabeled (GdnHCl) | 100.0 | - | - | - | - | - | 0.0 |
|  |  | Nonreduced  (GdnHCl, mPEG2-biotin) | 100.0 | - | - | - | - | - | 0.0 |
|  |  | Reduced  (GdnHCl, TCEP, mPEG2-biotin) | 4.2 | - | 4.4 | - | 91.7 | - | 3.7 |
|  | IGF1R4^C4-C25^  (Δ*T*_m_: 7.3°C) | Unlabeled (GdnHCl) | 100.0 | - | - | - | - | - | 0.0 |
|  |  | Nonreduced  (GdnHCl, mPEG2-biotin) | 96.5 | - | 3.5 | - | - | - | 0.1 |
|  |  | Reduced  (GdnHCl, TCEP, mPEG2-biotin) | 11.2 | - | 4.2 | - | 84.7 | - | 3.5 |
| Cys23-Cys104, Cys6-Cys119 | FC5^C6-C119^  (Δ*T*_m_: 7.3°C) | Unlabeled (GdnHCl) | 99.9 | - | 0.1 | - | - | - | 0.0 |
|  |  | Nonreduced  (GdnHCl, mPEG2-biotin) | 99.8 | - | 0.2 | - | - | - | 0.0 |
|  |  | Reduced  (GdnHCl, TCEP, mPEG2-biotin) | 10.7 | - | 22.4 | - | 66.9 | - | 3.1 |
|  | IGF1R4^C6-C119^  (Δ*T*_m_: 11.6°C) | Unlabeled (GdnHCl) | 100.0 | - | - | - | - | - | 0.0 |
|  |  | Nonreduced  (GdnHCl, mPEG2-biotin) | 99.1 | - | 0.8 | - | - | - | 0.0 |
|  |  | Reduced  (GdnHCl, TCEP, mPEG2-biotin) | 20.7 | - | 18.4 | 0.1 | 60.8 | - | 2.8 |

Abbreviations: GdnHCl, guanidine hydrochloride; mPEG2-biotin, maleimide-PEG2-biotin; n.a., not applicable; TCEP, tris(2-carboxyethyl)phosphine; WT, wild-type.

**Table S6. Metrics for Illumina MiSeq data analyzed in this study.**

| **V_H_ repertoire** | **No. of cells** | **Raw reads** | **Merged reads** | **Quality filtered reads** |
| --- | --- | --- | --- | --- |
| 1B | 100,000 | 77,674 | 47,953 | 42,642 |
| 2B | 100,000 | 175,818 | 95,253 | 83,402 |
| 3B | 100,000 | 271,896 | 177,043 | 154,871 |
| 4B | 100,000 | 163,156 | 101,691 | 90,159 |
| 6B | 100,000 | 86,733 | 19,520 | 17,018 |
| 7B | 100,000 | 149,953 | 105,675 | 93,280 |
| 9B | 100,000 | 102,704 | 93,733 | 79,950 |
| HIV2 | 480,000 | 30,972 | 26,975 | 20,621 |
| HIV8 | 450,000 | 26,344 | 22,450 | 16,976 |
| HIV14 | 360,000 | 144,466 | 126,467 | 95,368 |
| HIV26 | 390,000 | 266,904 | 232,239 | 175,471 |
| HIV32 | 150,000 | 209,935 | 185,650 | 141,612 |
| HIV44 | 400,000 | 77,624 | 64,820 | 48,878 |
| HIV50 | 110,000 | 379,716 | 306,398 | 236,380 |
| HIV56 | 360,000 | 164,313 | 123,338 | 31,390 |
| HIV62 | 500,000 | 117,838 | 75,869 | 54,704 |
| HIV68 | 340,000 | 150,029 | 112,822 | 87,754 |

**Table S7. Oligonucleotides for subcloning of V_H_s from pSJF2H into fd-tetGIIID.**

| **Name** | **Sequence (5’-3’)** | **Purpose** |
| --- | --- | --- |
| VHM81-F | catgca**gtgcac**aggaagtgcagctggtgcag | Subcloning of VHM81 and VHM81^C23-C104 null^ |
| VHM81-R | catgca**gcggccgc**tttggaaccagagcctgaagagacggtgaccattg |  |
| VHB82-F | catgca**gtgcac**aggtgcagctgcaggag | Subcloning of VHB82 and VHB82^C23-C104 null^ |
| VHB82-R | catgca**gcggccgc**tttggaaccagagcctgaggagacggtgaccgtg |  |
| VH413-F | catgca**gtgcac**aggtccagctggtgcag | Subcloning of VH413 and VH413^C23-C104 null^ |
| VH413-R | catgca**gcggccgc**tttggaaccagagcctgaagagacggtgaccattg |  |
| cPCR-F | gtgaaaaaattattattcgcaattcct | Colony PCR |
| cPCR-R | ccctcatagttagcgtaacg | Colony PCR |

Restriction enzyme recognition sequences are shown in bold text.

**Table S8**. **Oligonucleotides for Kunkel mutagenesis of fd-tetGIIID-VH413^C23-C104 null^ phage.**

| **Name** | **Sequence (5’-3’)** | **Purpose** |
| --- | --- | --- |
| 54-Cys | accgctataaataaagcagacccactccagtcc | Engineering of Cys54-Cys78 into VH413^C23-C104 null^ phage |
| 78-Cys | ggaattgtctctggagcaggtgaatcggccc |  |
| 40-Cys | agcctggcggacccagcacatgtagtagtcactg | Engineering of Cys40-Cys55 into VH413^C23-C104 null^ phage |
| 55-Cys | accaccgctataaatgcatgagacccactccag |  |
| 54-Cys | accgctataaataaagcagacccactccagtcc | Engineering of Cys54-Cys87 into VH413^C23-C104 null^ phage |
| 87-Cys | gttcatttgaagatagcacgtgttcttggaattg |  |

**Table S9**. **Sequences of mutagenic oligonucleotides used to construct 17 Cys pair scan phage-displayed VH413^C23-C104 null^ libraries.**

| **Oligo** | **Sequence (5’-3’)** | **β-strands** |
| --- | --- | --- |
| A1 | caccagctggacgcatgcactgtgagaatag | A |
| A2 | ctgcaccagctggcactgtgcactgtg | A |
| A3 | agactgcaccaggcagacctgtgcactg | A |
| A4 | cccagactgcacgcactggacctgtgc | A |
| A5 | cccagactggcacagctggacctg | A |
| A6 | gcctcccccagagcacaccagctggac | A |
| A7 | caagcctcccccgcactgcaccagctg | A |
| A8 | gaccaagcctccgcaagactgcaccag | A |
| A9 | cttgaccaagccgcacccagactgcac | A |
| A10 | aggcttgaccaagcatcccccagactg | A |
| A11 | tccaggcttgacgcagcctcccccagac | A |
| A12 | ccctccaggcttgcacaagcctccccc | A |
| A13 | ggaccctccagggcagaccaagcctcc | A |
| A14 | cagggaccctccgcacttgaccaagcc | A |
| B1 | agtctcagggacccgcaaggcttgaccaagcc | B |
| B2 | gagagtctcagggagcatccaggcttgaccaag | B |
| B3 | gcggagagtctcaggcaccctccaggcttgacc | B |
| B4 | gctgcggagagtctgcaggaccctccaggcttg | B |
| B5 | gctgctgcggagaggcacagggaccctccaggc | B |
| B6 | gaggctgctgcggagcatctcagggaccctcc | B |
| B7 | ccagaggctgctgcgcagagtctcagggaccc | B |
| B8 | aatccagaggctgcgcaggagagtctcagggac | B |
| B9 | gtgaatccagaggcgcatgcggagagtctcagg | B |
| B10 | aaggtgaatccagagcatgctgcggagagtctc | B |
| B11 | ctgaaggtgaatccgcaggctgctgcggagagtc | B |
| C1 | gcggacccagctgcagtagtagtcactg | C |
| C2 | ctggcggacccagcacatgtagtagtc | C |
| C3 | agcctggcggacgcagctcatgtagtag | C |
| C4 | tggagcctggcggcaccagctcatgtag | C |
| C5 | ccctggagcctggcagacccagctcatg | C |
| C6 | cttccctggagcgcagcggacccagctc | C |
| C7 | tcccttccctgggcactggcggacccag | C |
| C8 | cagtcccttcccgcaagcctggcggac | C |
| C’1 | ctccagtcccttgcatggagcctggcg | C' |
| C’2 | ccactccagtccgcaccctggagcctg | C' |
| C’3 | gacccactccaggcacttccctggagc | C' |
| C’4 | tgagacccactcgcatcccttccctgg | C’ |
| C’5 | aaatgagacccagcacagtcccttccc | C’ |
| C’6 | aataaatgagacgcactccagtcccttc | C’ |
| C’7 | ataaataaatgagcaccactccagtcc | C’ |
| C’8 | gctataaataaagcagacccactccag | C’ |
| C’9 | accgctataaatgcatgagacccactc | C’ |
| C”1 | ggagtctgcgtagcatgtgctaccacc | C" |
| C”2 | cacggagtctgcgcagtatgtgctacc | C" |
| C”3 | cttcacggagtcgcagtagtatgtgctac | C" |
| C”4 | gcccttcacggagcatgcgtagtatgtg | C" |
| C”5 | tcggcccttcacgcagtctgcgtagtatg | C" |
| C”6 | gaatcggcccttgcaggagtctgcgtag | C" |
| C”7 | ggtgaatcggccgcacacggagtctgc | C" |
| C”8 | gatggtgaatcggcacttcacggagtc | C" |
| D1 | ggagatggtgaagcagcccttcacggag | D |
| D2 | tctggagatggtgcatcggcccttcac | D |
| D3 | gtctctggagatgcagaatcggcccttc | D |
| D4 | attgtctctggagcaggtgaatcggcc | D |
| D5 | ggaattgtctctgcagatggtgaatcg | D |
| D6 | cttggaattgtcgcaggagatggtgaatc | D |
| D7 | gttcttggaattgcatctggagatggtg | D |
| D8 | cgtgttcttggagcagtctctggagatg | D |
| D9 | cagcgtgttcttgcaattgtctctggag | D |
| D10 | atacagcgtgttgcaggaattgtctctg | D |
| E1 | aagatacagcgtgcacttggaattgtc | E |
| E2 | ttgaagatacaggcagttcttggaattg | E |
| E3 | catttgaagatagcacgtgttcttggaattg | E |
| E4 | gttcatttgaaggcacagcgtgttcttg | E |
| E5 | gctgttcatttggcaatacagcgtgttc | E |
| E6 | caggctgttcatgcaaagatacagcgtg | E |
| E7 | tctcaggctgttgcattgaagatacag | E |
| E8 | agctctcaggctgcacatttgaagatac | E |
| E9 | ctcagctctcaggcagttcatttgaag | E |
| E10 | gtcctcagctctgcagctgttcatttg | E |
| E11 | cgtgtcctcagcgcacaggctgttcatttg | E |
| E12 | agccgtgtcctcgcatctcaggctgttc | E |
| F1 | atacacagccgtgtcgcaagctctcaggctgttc | F |
| F2 | taatacacagccgtgcactcagctctcaggctg | F |
| F3 | tgcgtaatacacagcgcagtcctcagctctcaggc | F |
| F4 | cgctgcgtaatacacgcacgtgtcctcagctc | F |
| F5 | tctcgctgcgtaatagcaagccgtgtcctcagc | F |
| F6 | ctctctcgctgcgtagcacacagccgtgtcctc | F |
| F7 | gctctctctcgctgcgcaatacacagccgtgtc | F |
| F8 | tctgctctctctcgcgcagtaatacacagccg | F |
| G1 | tgtcccttggccgcagatatcaaaagc | G |
| G2 | cattgtcccttggcaccagatatcaaaagc | G |
| G3 | gaccattgtcccgcagccccagatatc | G |
| G4 | ggtgaccattgtgcattggccccagatatc | G |
| G5 | gacggtgaccatgcacccttggccccag | G |
| G6 | agagacggtgacgcatgtcccttggcc | G |
| G7 | tgaagagacggtgcacattgtcccttg | G |
| G8 | gcctgaagagacgcagaccattgtccc | G |
| G9 | agagcctgaagagcaggtgaccattgtc | G |
| G10 | accagagcctgagcagacggtgaccattg | G |
| G11 | ggaaccagagccgcaagagacggtgac | G |
| A6-B1 | tctcagggacccgcaaggcttgaccaagcctcccccagagcacaccagctggac | A-B |
| A7-B1 | tctcagggacccgcaaggcttgaccaagcctcccccgcactgcaccagctgg | A-B |
| A7-B2 | gagtctcagggagcatccaggcttgaccaagcctcccccgcactgcaccagctg | A-B |
| A8-B1 | tctcagggacccgcaaggcttgaccaagcctccgcaagactgcaccag | A-B |
| A8-B2 | gagtctcagggagcatccaggcttgaccaagcctccgcaagactgcaccag | A-B |
| A8-B3 | ggagagtctcaggcaccctccaggcttgaccaagcctccgcaagactgcaccag | A-B |
| A9-B1 | tctcagggacccgcaaggcttgaccaagccgcacccagactgcac | A-B |
| A9-B2 | gagtctcagggagcatccaggcttgaccaagccgcacccagactgcac | A-B |
| A9-B3 | ggagagtctcaggcaccctccaggcttgaccaagccgcacccagactgcac | A-B |
| A9-B4 | tgcggagagtctgcaggaccctccaggcttgaccaagccgcacccagactgcac | A-B |
| A10-B1 | tctcagggacccgcaaggcttgaccaagcatcccccagactg | A-B |
| A10-B2 | gagtctcagggagcatccaggcttgaccaagcatcccccagactg | A-B |
| A10-B3 | ggagagtctcaggcaccctccaggcttgaccaagcatcccccagactg | A-B |
| A10-B4 | tgcggagagtctgcaggaccctccaggcttgaccaagcatcccccagactg | A-B |
| A10-B5 | tgctgcggagaggcacagggaccctccaggcttgaccaagcatcccccagactg | A-B |
| A11-B1 | tctcagggacccgcaaggcttgacgcagcctcccccagac | A-B |
| A11-B2 | gagtctcagggagcatccaggcttgacgcagcctcccccagac | A-B |
| A11-B3 | ggagagtctcaggcaccctccaggcttgacgcagcctcccccagac | A-B |
| A11-B4 | tgcggagagtctgcaggaccctccaggcttgacgcagcctcccccagac | A-B |
| A11-B5 | tgctgcggagaggcacagggaccctccaggcttgacgcagcctcccccagac | A-B |
| A11-B6 | ggctgctgcggagcatctcagggaccctccaggcttgacgcagcctcccccagac | A-B |
| A12-B1 | tctcagggacccgcaaggcttgcacaagcctccccc | A-B |
| A12-B2 | gagtctcagggagcatccaggcttgcacaagcctccccc | A-B |
| A12-B3 | ggagagtctcaggcaccctccaggcttgcacaagcctccccc | A-B |
| A12-B4 | tgcggagagtctgcaggaccctccaggcttgcacaagcctccccc | A-B |
| A12-B5 | tgctgcggagaggcacagggaccctccaggcttgcacaagcctccccc | A-B |
| A12-B6 | ggctgctgcggagcatctcagggaccctccaggcttgcacaagcctccccc | A-B |
| A12-B7 | agaggctgctgcgcagagtctcagggaccctccaggcttgcacaagcctccccc | A-B |
| A13-B1 | gagtctcagggacccgcaagggcagaccaagcctccccc | A-B |
| A13-B2 | gagtctcagggagcatccagggcagaccaagcctcc | A-B |
| A13-B3 | cggagagtctcaggcaccctccagggcagaccaagcctcc | A-B |
| A13-B4 | tgcggagagtctgcaggaccctccagggcagaccaagcctcc | A-B |
| A13-B5 | tgctgcggagaggcacagggaccctccagggcagaccaagcctcc | A-B |
| A13-B6 | ggctgctgcggagcatctcagggaccctccagggcagaccaagcctcc | A-B |
| A13-B7 | ggctgctgcgcagagtctcagggaccctccagggcagaccaagcctcc | A-B |
| A13-B8 | tccagaggctgcgcaggagagtctcagggaccctccagggcagaccaagcctcc | A-B |
| A14-B1 | ggagagtctcagggacccgcagcacttgaccaagcctccccc | A-B |
| A14-B2 | ggagagtctcagggagcatccgcacttgaccaagcctcc | A-B |
| A14-B3 | ctgcggagagtctcaggcaccctccgcacttgaccaagcctc | A-B |
| A14-B4 | gcggagagtctgcaggaccctccgcacttgaccaagcctc | A-B |
| A14-B5 | tgctgcggagaggcacagggaccctccgcacttgaccaagcc | A-B |
| A14-B6 | ggctgctgcggagcatctcagggaccctccgcacttgaccaagcc | A-B |
| A14-B7 | agaggctgctgcgcagagtctcagggaccctccgcacttgaccaagcctc | A-B |
| A14-B8 | tccagaggctgcgcaggagagtctcagggaccctccgcacttgaccaagcctc | A-B |
| A14-B9 | gaatccagaggcgcatgcggagagtctcagggaccctccgcacttgaccaagcctc | A-B |
| B11-C1 | gcggacccagctgcagtagtagtcactgaaggtgaatccgcaggctgctgcggag | B-C |
| C1-C’1 | ctccagtcccttgcatggagcctggcggacccagctgcagtagtagtcactg | C-C' |
| C1-C’2 | ccactccagtccgcaccctggagcctggcggacccagctgcagtagtagtcactg | C-C' |
| C1-C’3 | gacccactccaggcacttccctggagcctggcggacccagctgcagtagtagtcactg | C-C' |
| C2-C’1 | ctccagtcccttgcatggagcctggcggacccagcacatgtagtagtcac | C-C' |
| C2-C’2 | ccactccagtccgcaccctggagcctggcggacccagcacatgtagtagtcac | C-C' |
| C2-C’3 | gacccactccaggcacttccctggagcctggcggacccagcacatgtagtagtcac | C-C' |
| C2-C’4 | tgagacccactcgcatcccttccctggagcctggcggacccagcacatgtagtagtcac | C-C' |
| C3-C’1 | ctccagtcccttgcatggagcctggcggacgcagctcatgtagtag | C-C' |
| C3-C’2 | ccactccagtccgcaccctggagcctggcggacgcagctcatgtagtag | C-C' |
| C3-C’3 | gacccactccaggcacttccctggagcctggcggacgcagctcatgtagtag | C-C' |
| C3-C’4 | tgagacccactcgcatcccttccctggagcctggcggacgcagctcatgtagtag | C-C' |
| C3-C’5 | aaatgagacccagcacagtcccttccctggagcctggcggacgcagctcatgtagtag | C-C' |
| C4-C’1 | ctccagtcccttgcatggagcctggcggcaccagctcatgtag | C-C' |
| C4-C’2 | ccactccagtccgcaccctggagcctggcggcaccagctcatgtag | C-C' |
| C4-C’3 | gacccactccaggcacttccctggagcctggcggcaccagctcatgtag | C-C' |
| C4-C’4 | tgagacccactcgcatcccttccctggagcctggcggcaccagctcatgtag | C-C' |
| C4-C’5 | aaatgagacccagcacagtcccttccctggagcctggcggcaccagctcatgtag | C-C' |
| C4-C’6 | aataaatgagacgcactccagtcccttccctggagcctggcggcaccagctcatgtag | C-C' |
| C5-C’1 | ctccagtcccttgcatggagcctggcagacccagctcatg | C-C' |
| C5-C’2 | ccactccagtccgcaccctggagcctggcagacccagctcatg | C-C' |
| C5-C’3 | gacccactccaggcacttccctggagcctggcagacccagctcatg | C-C' |
| C5-C’4 | tgagacccactcgcatcccttccctggagcctggcagacccagctcatg | C-C' |
| C5-C’5 | aaatgagacccagcacagtcccttccctggagcctggcagacccagctcatg | C-C' |
| C5-C’6 | aataaatgagacgcactccagtcccttccctggagcctggcagacccagctcatg | C-C' |
| C5-C’7 | ataaataaatgagcaccactccagtcccttccctggagcctggcagacccagctcatg | C-C' |
| C6-C’1 | ctccagtcccttgcatggagcgcagcggacccagctc | C-C' |
| C6-C’2 | ccactccagtccgcaccctggagcgcagcggacccagctc | C-C' |
| C6-C’3 | gacccactccaggcacttccctggagcgcagcggacccagctc | C-C' |
| C6-C’4 | tgagacccactcgcatcccttccctggagcgcagcggacccagctc | C-C' |
| C6-C’5 | aaatgagacccagcacagtcccttccctggagcgcagcggacccagctc | C-C' |
| C6-C’6 | aataaatgagacgcactccagtcccttccctggagcgcagcggacccagctc | C-C' |
| C6-C’7 | ataaataaatgagcaccactccagtcccttccctggagcgcagcggacccagctc | C-C' |
| C6-C’8 | gctataaataaagcagacccactccagtcccttccctggagcgcagcggacccagctc | C-C' |
| C7-C’1 | ctccagtcccttgcatgggcactggcggacccag | C-C' |
| C7-C’2 | ccactccagtccgcaccctgggcactggcggacccag | C-C' |
| C7-C’3 | gacccactccaggcacttccctgggcactggcggacccag | C-C' |
| C7-C’4 | tgagacccactcgcatcccttccctgggcactggcggacccag | C-C' |
| C7-C’5 | aaatgagacccagcacagtcccttccctgggcactggcggacccag | C-C' |
| C7-C’6 | aataaatgagacgcactccagtcccttccctgggcactggcggacccag | C-C' |
| C7-C’7 | ataaataaatgagcaccactccagtcccttccctgggcactggcggacccag | C-C' |
| C7-C’8 | gctataaataaagcagacccactccagtcccttccctgggcactggcggacccag | C-C' |
| C7-C’9 | accgctataaatgcatgagacccactccagtcccttccctgggcactggcggacccag | C-C' |
| C8-C’1 | cccactccagtcccttgcagcaagcctggcggacccag | C-C' |
| C8-C’2 | gacccactccagtccgcacccgcaagcctggcggacccag | C-C' |
| C8-C’3 | tgagacccactccaggcacttcccgcaagcctggcggacccag | C-C' |
| C8-C’4 | tgagacccactcgcatcccttcccgcaagcctggcggac | C-C' |
| C8-C’5 | aaatgagacccagcacagtcccttcccgcaagcctggcggac | C-C' |
| C8-C’6 | aataaatgagacgcactccagtcccttcccgcaagcctggcggac | C-C' |
| C8-C’7 | ataaataaatgagcaccactccagtcccttcccgcaagcctggcggac | C-C' |
| C8-C’8 | gctataaataaagcagacccactccagtcccttcccgcaagcctggcggac | C-C' |
| C8-C’9 | accgctataaatgcatgagacccactccagtcccttcccgcaagcctggcggac | C-C' |
| C’8-C”1 | ggagtctgcgtagcatgtgctaccaccgctataaataaagcagacccactccag | C'-C" |
| C’9-C”1 | ggagtctgcgtagcatgtgctaccaccgctataaatgcatgagacccactc | C'-C" |
| C’9-C”2 | cacggagtctgcgcagtatgtgctaccaccgctataaatgcatgagacccactc | C'-C" |
| C”1-D1 | ggtggtagcacatgctacgcagactccgtgaagggctgcttcaccatctcc | C"-D |
| C”1-D2 | ggtggtagcacatgctacgcagactccgtgaagggccgatgcaccatctccaga | C"-D |
| C”2-D1 | ggtagcacatactgcgcagactccgtgaagggctgcttcaccatctcc | C"-D |
| C”2-D2 | ggtagcacatactgcgcagactccgtgaagggccgatgcaccatctccaga | C"-D |
| C”2-D3 | ggtagcacatactgcgcagactccgtgaagggccgattctgcatctccagagac | C"-D |
| C”3-D1 | gtagcacatactactgcgactccgtgaagggctgcttcaccatctcc | C"-D |
| C”3-D2 | gtagcacatactactgcgactccgtgaagggccgatgcaccatctccaga | C"-D |
| C”3-D3 | gtagcacatactactgcgactccgtgaagggccgattctgcatctccagagac | C"-D |
| C”3-D4 | gtagcacatactactgcgactccgtgaagggccgattcacctgctccagagacaat | C"-D |
| C”4-D1 | cacatactacgcatgctccgtgaagggctgcttcaccatctcc | C"-D |
| C”4-D2 | cacatactacgcatgctccgtgaagggccgatgcaccatctccaga | C"-D |
| C”4-D3 | cacatactacgcatgctccgtgaagggccgattctgcatctccagagac | C"-D |
| C”4-D4 | cacatactacgcatgctccgtgaagggccgattcacctgctccagagacaat | C"-D |
| C”4-D5 | cacatactacgcatgctccgtgaagggccgattcaccatctgcagagacaattcc | C"-D |
| C”5-D1 | catactacgcagactgcgtgaagggctgcttcaccatctcc | C"-D |
| C”5-D2 | catactacgcagactgcgtgaagggccgatgcaccatctccaga | C"-D |
| C”5-D3 | catactacgcagactgcgtgaagggccgattctgcatctccagagac | C"-D |
| C”5-D4 | catactacgcagactgcgtgaagggccgattcacctgctccagagacaat | C"-D |
| C”5-D5 | catactacgcagactgcgtgaagggccgattcaccatctgcagagacaattcc | C"-D |
| C”5-D6 | catactacgcagactgcgtgaagggccgattcaccatctcctgcgacaattccaag | C"-D |
| C”6-D1 | ctacgcagactcctgcaagggctgcttcaccatctccaga | C"-D |
| C”6-D2 | ctacgcagactcctgcaagggccgatgcaccatctccaga | C"-D |
| C”6-D3 | ctacgcagactcctgcaagggccgattctgcatctccagagac | C"-D |
| C”6-D4 | ctacgcagactcctgcaagggccgattcacctgctccagagacaat | C"-D |
| C”6-D5 | ctacgcagactcctgcaagggccgattcaccatctgcagagacaattcc | C"-D |
| C”6-D6 | ctacgcagactcctgcaagggccgattcaccatctcctgcgacaattccaag | C"-D |
| C”6-D7 | ctacgcagactcctgcaagggccgattcaccatctccagatgcaattccaagaac | C"-D |
| C”7-D1 | ctacgcagactccgtgtgcggctgcttcaccatctccaga | C"-D |
| C”7-D2 | ctacgcagactccgtgtgcggccgatgcaccatctccagagac | C"-D |
| C”7-D3 | gcagactccgtgtgcggccgattctgcatctccagagac | C"-D |
| C”7-D4 | gcagactccgtgtgcggccgattcacctgctccagagacaat | C"-D |
| C”7-D5 | gcagactccgtgtgcggccgattcaccatctgcagagacaattcc | C"-D |
| C”7-D6 | gcagactccgtgtgcggccgattcaccatctcctgcgacaattccaag | C"-D |
| C”7-D7 | gcagactccgtgtgcggccgattcaccatctccagatgcaattccaagaac | C"-D |
| C”7-D8 | gcagactccgtgtgcggccgattcaccatctccagagactgctccaagaacacg | C"-D |
| C”8-D1 | cgcagactccgtgaagtgctgcttcaccatctccaga | C"-D |
| C”8-D2 | cgcagactccgtgaagtgccgatgcaccatctccagagac | C"-D |
| C”8-D3 | cgcagactccgtgaagtgccgattctgcatctccagagacaat | C"-D |
| C”8-D4 | gactccgtgaagtgccgattcacctgctccagagacaat | C"-D |
| C”8-D5 | gactccgtgaagtgccgattcaccatctgcagagacaattcc | C"-D |
| C”8-D6 | gactccgtgaagtgccgattcaccatctcctgcgacaattccaag | C"-D |
| C”8-D7 | gactccgtgaagtgccgattcaccatctccagatgcaattccaagaac | C"-D |
| C”8-D8 | gactccgtgaagtgccgattcaccatctccagagactgctccaagaacacg | C"-D |
| C”8-D9 | gactccgtgaagtgccgattcaccatctccagagacaattgcaagaacacgctg | C"-D |
| D2-E1 | aagatacagcgtgcacttggaattgtctctggagatggtgcatcggcccttcac | D-E |
| D3-E1 | aagatacagcgtgcacttggaattgtctctggagatgcagaatcggcccttc | D-E |
| D3-E2 | ttgaagatacaggcagttcttggaattgtctctggagatgcagaatcggcccttc | D-E |
| D4-E1 | aagatacagcgtgcacttggaattgtctctggagcaggtgaatcggcc | D-E |
| D4-E2 | ttgaagatacaggcagttcttggaattgtctctggagcaggtgaatcggcc | D-E |
| D4-E3 | catttgaagatagcacgtgttcttggaattgtctctggagcaggtgaatcggcc | D-E |
| D5-E1 | aagatacagcgtgcacttggaattgtctctgcagatggtgaatcg | D-E |
| D5-E2 | ttgaagatacaggcagttcttggaattgtctctgcagatggtgaatcg | D-E |
| D5-E3 | catttgaagatagcacgtgttcttggaattgtctctgcagatggtgaatcg | D-E |
| D5-E4 | gttcatttgaaggcacagcgtgttcttggaattgtctctgcagatggtgaatcg | D-E |
| D6-E1 | aagatacagcgtgcacttggaattgtcgcaggagatggtgaatc | D-E |
| D6-E2 | ttgaagatacaggcagttcttggaattgtcgcaggagatggtgaatc | D-E |
| D6-E3 | catttgaagatagcacgtgttcttggaattgtcgcaggagatggtgaatc | D-E |
| D6-E4 | gttcatttgaaggcacagcgtgttcttggaattgtcgcaggagatggtgaatc | D-E |
| D6-E5 | gctgttcatttggcaatacagcgtgttcttggaattgtcgcaggagatggtgaatc | D-E |
| D7-E1 | aagatacagcgtgcacttggaattgcatctggagatggtg | D-E |
| D7-E2 | ttgaagatacaggcagttcttggaattgcatctggagatggtg | D-E |
| D7-E3 | catttgaagatagcacgtgttcttggaattgcatctggagatggtg | D-E |
| D7-E4 | gttcatttgaaggcacagcgtgttcttggaattgcatctggagatggtg | D-E |
| D7-E5 | gctgttcatttggcaatacagcgtgttcttggaattgcatctggagatggtg | D-E |
| D7-E6 | caggctgttcatgcaaagatacagcgtgttcttggaattgcatctggagatggtg | D-E |
| D8-E1 | aagatacagcgtgcacttggagcagtctctggagatg | D-E |
| D8-E2 | ttgaagatacaggcagttcttggagcagtctctggagatg | D-E |
| D8-E3 | catttgaagatagcacgtgttcttggagcagtctctggagatg | D-E |
| D8-E4 | gttcatttgaaggcacagcgtgttcttggagcagtctctggagatg | D-E |
| D8-E5 | gctgttcatttggcaatacagcgtgttcttggagcagtctctggagatg | D-E |
| D8-E6 | caggctgttcatgcaaagatacagcgtgttcttggagcagtctctggagatg | D-E |
| D8-E7 | tctcaggctgttgcattgaagatacagcgtgttcttggagcagtctctggagatg | D-E |
| D9-E1 | aagatacagcgtgcacttgcaattgtctctggag | D-E |
| D9-E2 | ttgaagatacaggcagttcttgcaattgtctctggag | D-E |
| D9-E3 | catttgaagatagcacgtgttcttgcaattgtctctggag | D-E |
| D9-E4 | gttcatttgaaggcacagcgtgttcttgcaattgtctctggag | D-E |
| D9-E5 | gctgttcatttggcaatacagcgtgttcttgcaattgtctctggag | D-E |
| D9-E6 | caggctgttcatgcaaagatacagcgtgttcttgcaattgtctctggag | D-E |
| D9-E7 | tctcaggctgttgcattgaagatacagcgtgttcttgcaattgtctctggag | D-E |
| D9-E8 | agctctcaggctgcacatttgaagatacagcgtgttcttgcaattgtctctggag | D-E |
| D10-E1 | aagatacagcgtgcagcaggaattgtctctg | D-E |
| D10-E2 | ttgaagatacaggcagttgcaggaattgtctctg | D-E |
| D10-E3 | catttgaagatagcacgtgttgcaggaattgtctctg | D-E |
| D10-E4 | gttcatttgaaggcacagcgtgttgcaggaattgtctctg | D-E |
| D10-E5 | gctgttcatttggcaatacagcgtgttgcaggaattgtctctg | D-E |
| D10-E6 | caggctgttcatgcaaagatacagcgtgttgcaggaattgtctctg | D-E |
| D10-E7 | tctcaggctgttgcattgaagatacagcgtgttgcaggaattgtctctg | D-E |
| D10-E8 | agctctcaggctgcacatttgaagatacagcgtgttgcaggaattgtctctg | D-E |
| D10-E9 | ctcagctctcaggcagttcatttgaagatacagcgtgttgcaggaattgtctctg | D-E |
| E4-F1 | cacagccgtgtcgcaagctctcaggctgttcatttgaaggcacagcgtgttcttg | E-F |
| E5-F1 | cacagccgtgtcgcaagctctcaggctgttcatttggcaatacagcgtgttc | E-F |
| E5-F2 | atacacagccgtgcactcagctctcaggctgttcatttggcaatacagcgtgttc | E-F |
| E6-F1 | cacagccgtgtcgcaagctctcaggctgttcatgcaaagatacagcgtg | E-F |
| E6-F2 | atacacagccgtgcactcagctctcaggctgttcatgcaaagatacagcgtg | E-F |
| E6-F3 | gtaatacacagcgcagtcctcagctctcaggctgttcatgcaaagatacagcgtg | E-F |
| E7-F1 | cacagccgtgtcgcaagctctcaggctgttgcattgaagatacag | E-F |
| E7-F2 | atacacagccgtgcactcagctctcaggctgttgcattgaagatacag | E-F |
| E7-F3 | gtaatacacagcgcagtcctcagctctcaggctgttgcattgaagatacag | E-F |
| E7-F4 | tgcgtaatacacgcacgtgtcctcagctctcaggctgttgcattgaagatacag | E-F |
| E8-F1 | cacagccgtgtcgcaagctctcaggctgcacatttgaagatac | E-F |
| E8-F2 | atacacagccgtgcactcagctctcaggctgcacatttgaagatac | E-F |
| E8-F3 | gtaatacacagcgcagtcctcagctctcaggctgcacatttgaagatac | E-F |
| E8-F4 | tgcgtaatacacgcacgtgtcctcagctctcaggctgcacatttgaagatac | E-F |
| E8-F5 | cgctgcgtaatagcaagccgtgtcctcagctctcaggctgcacatttgaagatac | E-F |
| E9-F1 | cacagccgtgtcgcaagctctcaggcagttcatttgaag | E-F |
| E9-F2 | atacacagccgtgcactcagctctcaggcagttcatttgaag | E-F |
| E9-F3 | gtaatacacagcgcagtcctcagctctcaggcagttcatttgaag | E-F |
| E9-F4 | tgcgtaatacacgcacgtgtcctcagctctcaggcagttcatttgaag | E-F |
| E9-F5 | cgctgcgtaatagcaagccgtgtcctcagctctcaggcagttcatttgaag | E-F |
| E9-F6 | tctcgctgcgtagcacacagccgtgtcctcagctctcaggcagttcatttgaag | E-F |
| E10-F1 | atacacagccgtgtcgcaagctctgcagctgttcatttgaag | E-F |
| E10-F2 | atacacagccgtgcactcagctctgcagctgttcatttg | E-F |
| E10-F3 | gtaatacacagcgcagtcctcagctctgcagctgttcatttg | E-F |
| E10-F4 | tgcgtaatacacgcacgtgtcctcagctctgcagctgttcatttg | E-F |
| E10-F5 | cgctgcgtaatagcaagccgtgtcctcagctctgcagctgttcatttg | E-F |
| E10-F6 | tctcgctgcgtagcacacagccgtgtcctcagctctgcagctgttcatttg | E-F |
| E10-F7 | ctctctcgctgcgcaatacacagccgtgtcctcagctctgcagctgttcatttg | E-F |
| E11-F1 | atacacagccgtgtcgcaagcgcacaggctgttcatttg | E-F |
| E11-F2 | gtaatacacagccgtgcactcagcgcacaggctgttcatttg | E-F |
| E11-F3 | gtaatacacagcgcagtcctcagcgcacaggctgttcatttg | E-F |
| E11-F4 | tgcgtaatacacgcacgtgtcctcagcgcacaggctgttcatttg | E-F |
| E11-F5 | cgctgcgtaatagcaagccgtgtcctcagcgcacaggctgttcatttg | E-F |
| E11-F6 | tctcgctgcgtagcacacagccgtgtcctcagcgcacaggctgttcatttg | E-F |
| E11-F7 | ctctctcgctgcgcaatacacagccgtgtcctcagcgcacaggctgttcatttg | E-F |
| E11-F8 | gctctctctcgcgcagtaatacacagccgtgtcctcagcgcacaggctgttcatttg | E-F |
| E12-F1 | atacacagccgtgtcgcagcatctcaggctgttcatttg | E-F |
| E12-F2 | gtaatacacagccgtgcactcgcatctcaggctgttcatttg | E-F |
| E12-F3 | tgcgtaatacacagcgcagtcctcgcatctcaggctgttcatttg | E-F |
| E12-F4 | tgcgtaatacacgcacgtgtcctcgcatctcaggctgttc | E-F |
| E12-F5 | cgctgcgtaatagcaagccgtgtcctcgcatctcaggctgttc | E-F |
| E12-F6 | tctcgctgcgtagcacacagccgtgtcctcgcatctcaggctgttc | E-F |
| E12-F7 | ctctctcgctgcgcaatacacagccgtgtcctcgcatctcaggctgttc | E-F |
| E12-F8 | gctctctctcgcgcagtaatacacagccgtgtcctcgcatctcaggctgttc | E-F |

Note: names of mutagenesis oligonucleotides spanning two contiguous β-strands in the primary amino acid

sequence are shown in red font.
